# Early prediction of acute necrotizing pancreatitis by artificial intelligence: a prospective cohort-analysis of 2387 cases

**DOI:** 10.1038/s41598-022-11517-w

**Published:** 2022-05-12

**Authors:** Szabolcs Kiss, József Pintér, Roland Molontay, Marcell Nagy, Nelli Farkas, Zoltán Sipos, Péter Fehérvári, László Pecze, Mária Földi, Áron Vincze, Tamás Takács, László Czakó, Ferenc Izbéki, Adrienn Halász, Eszter Boros, József Hamvas, Márta Varga, Artautas Mickevicius, Nándor Faluhelyi, Orsolya Farkas, Szilárd Váncsa, Rita Nagy, Stefania Bunduc, Péter Jenő Hegyi, Katalin Márta, Katalin Borka, Attila Doros, Nóra Hosszúfalusi, László Zubek, Bálint Erőss, Zsolt Molnár, Andrea Párniczky, Péter Hegyi, Andrea Szentesi, Hungarian Pancreatic Study Group, Szabolcs Kiss, Nelli Farkas, Zoltán Sipos, Péter Fehérvári, László Pecze, Mária Földi, Áron Vincze, Tamás Takács, László Czakó, Ferenc Izbéki, Adrienn Halász, Eszter Boros, József Hamvas, Márta Varga, Artautas Mickevicius, Nándor Faluhelyi, Orsolya Farkas, Szilárd Váncsa, Rita Nagy, Stefania Bunduc, Péter Jenő Hegyi, Katalin Márta, Katalin Borka, Attila Doros, Nóra Hosszúfalusi, László Zubek, Bálint Erőss, Zsolt Molnár, Andrea Párniczky, Péter Hegyi, Andrea Szentesi, Judit Bajor, Szilárd Gódi, Patrícia Sarlós, József Czimmer, Imre Szabó, Gabriella Pár, Anita Illés, Roland Hágendorn, Balázs Csaba Németh, Balázs Kui, Dóra Illés, László Gajdán, Veronika Dunás-Varga, Roland Fejes, Mária Papp, Zsuzsanna Vitális, János Novák, Imola Török, Melania Macarie, Elena Ramírez-Maldonado, Ville Sallinen, Shamil Galeev, Barnabás Bod, Ali Tüzün Ince, Dániel Pécsi, Péter Varjú, Márk Félix Juhász, Klementina Ocskay, Alexandra Mikó, Zsolt Szakács

**Affiliations:** 1 Doctoral School of Clinical Medicine, Faculty of Medicine, University of Szeged, Szeged, 6720 Hungary; 2 Institute for Translational Medicine, Szentágothai Research Centre, Medical School, University of Pécs, Szigeti út 12., II. Emelet, Pécs, 7624 Hungary; 3 Heim Pál National Pediatric Institute, Üllői út 86, Budapest, 1089 Hungary; 4 Human and Social Data Science Lab, Budapest University of Technology and Economics, Műegyetem rkp. 3, Budapest, 1111 Hungary; 5 Stochastics Research Group, Hungarian Academy of Sciences, Budapest University of Technology and Economics, Egry József u. 1, Budapest, 1111 Hungary; 6 Institute of Bioanalysis, Medical School, University of Pécs, Honvéd u. 1, Pécs, 7624 Hungary; 7 Department of Biomathematics and Informatics, University of Veterinary Medicine, István u. 2, Budapest, 1078 Hungary; 8 Division of Gastroenterology, First Department of Medicine, Medical School, University of Pécs, Ifjúság út 13, Pécs, 7624 Hungary; 9 Department of Medicine, University of Szeged, Kálvária sgt. 57, Szeged, 6725 Hungary; 10 Department of Internal Medicine, Szent György Teaching Hospital of County Fejér, Seregélyesi út 3, Székesfehérvár, 8000 Hungary; 11 Bajcsy-Zsilinszky Hospital, Maglódi út 89-91, Budapest, 1106 Hungary; 12 Department of Gastroenterology, BMKK Dr Rethy Pal Hospital, Gyulai út 18, Békéscsaba, 5600 Hungary; 13 Vilnius University Hospital Santaros Clinics, Clinics of Abdominal Surgery, Nephrourology and Gastroenterology, Faculty of Medicine, Vilnius University, Santariškių g. 2, 08410 Vilnius, Lithuania; 14 Department of Medical Imaging, Medical School, University of Pécs, Ifjúság út 13, Pécs, 7624 Hungary; 15 Centre for Translational Medicine, Semmelweis University, Üllői út 26, Budapest, 1085 Hungary; 16 Doctoral School, Carol Davila University of Medicine and Pharmacy, Bulevardul Eroii Sanitari 8, 050474 Bucharest, Romania; 17 Division of Pancreatic Diseases, Heart and Vascular Center, Semmelweis University, Baross u. 23, Budapest, 1082 Hungary; 18 2nd Department of Pathology, Semmelweis University, Üllői út 93, Budapest, 1091 Hungary; 19 Department of Transplantation and Surgery, Semmelweis University, Baross u. 23, Budapest, 1082 Hungary; 20 Department of Internal Medicine and Hematology, Semmelweis University, Szentkirályi u. 46, Budapest, 1088 Hungary; 21 Department of Anaesthesiology and Intensive Therapy, Semmelweis University, Üllői út 78, Budapest, 1082 Hungary; 22 Department of Anaesthesiology and Intensive Therapy, Poznan University of Medical Sciences, ul. św. Marii Magdaleny 14, 61861 Poznan, Wielkopolska Poland; 23 Division of Gastroenterology, Department of Internal Medicine, University of Debrecen, Debrecen, Hungary; 24 Pándy Kálmán Hospital of Békés County, Gyula, Hungary; 25 County Emergency Clinical Hospital of Târgu Mures-Gastroenterology Clinic, University of Medicine, Pharmacy, Sciences and Technology “George Emil Palade”, Targu Mures, Romania; 26 General Surgery, Consorci Sanitari del Garraf, Sant Pere de Ribes, Barcelona, Spain; 27 Department of Transplantation and Liver Surgery, Helsinki University Hospital, University of Helsinki, Helsinki, Finland; 28 Saint Luke Clinical Hospital, St. Petersburg, Russia; 29 Dr. Bugyi István Hospital, Szentes, Hungary; 30 Hospital of Bezmialem Vakif University, School of Medicine, Istanbul, Turkey; 31 Department of Medical Genetics, Medical School, University of Pécs, Pécs, Hungary; 32 First Department of Medicine, Medical School, University of Pécs, Pécs, Hungary

**Keywords:** Gastroenterology, Gastrointestinal diseases, Machine learning

## Abstract

Pancreatic necrosis is a consistent prognostic factor in acute pancreatitis (AP). However, the clinical scores currently in use are either too complicated or require data that are unavailable on admission or lack sufficient predictive value. We therefore aimed to develop a tool to aid in necrosis prediction. The XGBoost machine learning algorithm processed data from 2387 patients with AP. The confidence of the model was estimated by a bootstrapping method and interpreted via the 10th and the 90th percentiles of the prediction scores. Shapley Additive exPlanations (SHAP) values were calculated to quantify the contribution of each variable provided. Finally, the model was implemented as an online application using the Streamlit Python-based framework. The XGBoost classifier provided an AUC value of 0.757. Glucose, C-reactive protein, alkaline phosphatase, gender and total white blood cell count have the most impact on prediction based on the SHAP values. The relationship between the size of the training dataset and model performance shows that prediction performance can be improved. This study combines necrosis prediction and artificial intelligence. The predictive potential of this model is comparable to the current clinical scoring systems and has several advantages over them.

## Introduction

Acute pancreatitis (AP) affects about 34 per 100,000 people per year, and it is the most frequent gastrointestinal disease requiring acute hospitalization^1,2^. The overall mortality is around 3%^3,4^; however, in about 10–20% of AP cases, acute necrotizing pancreatitis (ANP) develops, thus further increasing the risk of morbidity and mortality^5,6^. The overall mortality of ANP is approximately 15–20%, of which there is a further twofold increase in a third of ANP cases where the necrotic tissue becomes infected^7,8^.

Early appraisal of severity and prognosis is crucial in AP, particularly on clinical admission, to identify patients at risk of developing life-threatening complications. In these cases, close monitoring and early intervention may prevent organ dysfunction and a fatal outcome^9,10^.

It has long been known that necrosis is a consistent prognostic factor in AP^9^. The diagnosis of this local complication strongly relies on contrast-enhanced computer tomography (CECT) because it has a much higher sensitivity to detect ANP than ultrasonography^7^. Despite being the gold standard method for diagnosing ANP, CECT has many disadvantages: (1) ANP usually becomes apparent only 72 h after the onset of symptoms; (2) early and inappropriate CECT may prolong hospitalization; and (3) it is not accessible in every case^11^. There is therefore a need for other methods to supplement ANP assessment.

As the underlying pathophysiology of AP becomes more and more familiar by the accumulation of scientific data, several potential therapeutic targets have been identified^12,13^. Since some of these specific therapies may be available soon, prompt initiation of treatment after early identification of ANP could be even more important.

Since ANP is associated with life-threatening complications and increased mortality and it is the principal determinant of the incidence of secondary infection in AP^14^, researchers have endeavored to find an accurate clinical scoring system or biomarker that can predict ANP, the severe disease course or mortality itself. As regards ANP, these systems are either too complicated or require data that is unavailable in the initial stage of hospitalization or lack sufficient sensitivity and specificity. They are therefore rarely used in everyday clinical practice.

As artificial intelligence (AI) can overcome the limitations provided by the complexity of the data and time-dependent variables, the number of AI tools is increasing in medicine^15^. AI applications in pancreatic diseases are also evolving quickly^16^. Four AI models aimed to predict the severity of AP on clinical admission, all of which seems to outperform the conventional prediction scores^17–19^. Despite their promising preliminary results, these AI tools are limited by the overlap between the patient group used for model preparation and internal validation and the relatively low patient number.

This study has two main goals: first, to overcome these limitations and build an AI model that provides an accurate prediction for ANP development; and, second, to create an online tool from the model that could aid physicians in the early prognosis of AP.

## Methods

This study was reported following the Transparent Reporting of a multivariable prediction model for Individual Prognosis or Diagnosis (TRIPOD) Statement^20^. Ethics approval was obtained from the Hungarian Medical Research Council’s Scientific and Research Ethics Committee (22254-1/2012/EKU, 17787-8/2020/EÜIG). Written informed consent was obtained from all participants before enrolment. The study was conducted in accordance with the Helsinki Declaration.

### Data source and eligibility criteria

The analyzed dataset was collected by the Hungarian Pancreatic Study Group between 2012 and 2019. There were 2461 adult patients enrolled in the patient registry from 30 centers across 13 countries (Appendix A). All patients fulfilled two out of three AP diagnostic criteria based on the revised Atlanta classification^21^. Data were collected by physicians and trained clinical administrators on admission and each day during the whole hospital stay and were stored both on paper and electronically. Relevant clinical data underwent a four-level quality check system before analysis.

In all cases deemed eligible a CECT was performed during hospitalization to assess pancreatic necrosis formation. Exclusion criteria were as follows: (1) no pancreas imaging had been performed; and (2) the mere suspicion of necrosis formation by imaging, which was not confirmed later by CECT.

### Groups, outcomes, and predictors analyzed

Eligible participants were divided into two groups: (1) pancreatic necrosis formation was confirmed by a radiologist by CECT during hospitalization; and (2) absence of necrosis development. The dataset was analyzed and compared accordingly.

ANP was defined as lack of parenchymal enhancement or findings of peripancreatic necrosis such as an acute necrotic collection on CECT^22^. Other local (acute peripancreatic fluid collection and pseudocyst) and systemic (new-onset diabetes, heart failure, renal failure, and respiratory failure) complications and disease severity were defined based on the revised Atlanta classification^21^. Data on in-hospital mortality, length of hospital stay, and etiology of AP were also collected.

The assessed predictors of ANP were gender, age, body mass index (BMI), and laboratory parameters measured in the first 24 h of clinical admission. The following were evaluated: alanine transaminase, albumin, amylase, alkaline phosphatase (ALP), aspartate transaminase, blood urea nitrogen, calcium, C-reactive protein (CRP), creatinine, direct bilirubin, gamma-glutamyl transferase (GGT), glucose, estimated glomerular filtration rate (eGFR), glycated hemoglobin (HbA1c), hematocrit, hemoglobin, lactate dehydrogenase (LDH), lipase, potassium, procalcitonin, red blood cell count, sodium, thrombocyte, total bilirubin, total cholesterol, total protein, total white blood cell count (WBC), and triglyceride.

### Predictive modelling

**Figure 1:**
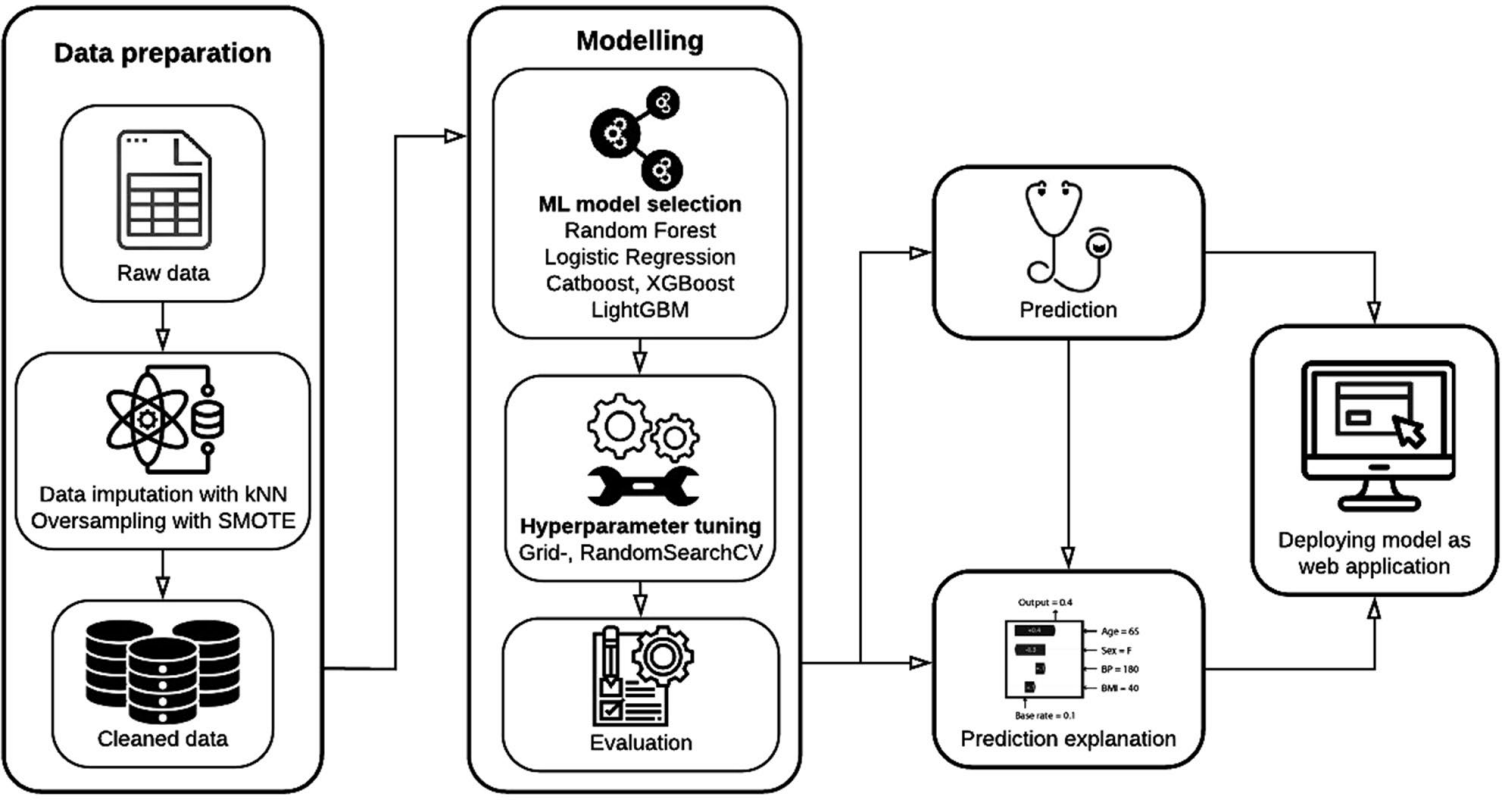
Flowchart representing the process of developing the model.

The process of predictive modelling is depicted in Fig. 1. Thirty-one variables have been used for modelling. Data quality is provided in Appendix A. Missing data were handled with a k-nearest-neighbor-based data imputer algorithm (KNNImputer)^23^. The SMOTE algorithm^24^ was used to deal with the imbalance in class distribution (number of patients with and without ANP).

Random Forest, Logistic Regression, Catboost, XGBoost, and LightGBM were tested for modelling to identify the best performing machine learning algorithm^25–28^. The catboost, xgboost, lightgbm, and scikit-learn Python packages were applied. The optimal model was chosen based on the receiver operating characteristic (ROC) curve and area under the ROC curve (AUC) value after performing four-fold cross-validation. The confidence of the best performing model was estimated with a bootstrapping method, namely by re-sampling the training dataset and training a hundred independent copies of the model on these datasets. The confidence of the model prediction was interpreted with the aid of the 10th and the 90th percentiles of the prediction scores.

Shapley Additive exPlanations (SHAP) values were calculated^29^ to locally explain the model prediction and to quantify the contribution of each variable provided. Finally, the model was deployed as an online application using the Streamlit Python-based framework.

### Other statistical analyses

The presence of sampling bias was tested by assessing the representativeness between the cohort analyzed and the whole cohort (Appendix A). The prediction parameters were also compared between patients with and without ANP with the Kolmogorov–Smirnov test and the Chi-squared test. ANP was tested as a risk factor for mortality, severe AP, and local and systemic complications by calculating risk ratios (RR) with the corresponding 95% confidence interval (CI).

### Ethics approval

Ethics approval was obtained from the Hungarian Medical Research Council’s Scientific and Research Ethics Committee (22254-1/2012/EKU, 17787-8/2020/EÜIG). The study was conducted in accordance with the Helsinki Declaration.

### Consent to participate

Written informed consent was obtained from all participants before enrolment.

### Consent for publication

The corresponding author accepts responsibility for releasing this material on behalf of all co-authors.

## Results

### Characteristics of the cohort analyzed

2387 of the 2461 patients with AP proved to be eligible for the analysis. Characteristics of this population are summarized in Table 1. In 9.76% of the cases, ANP was confirmed. There was a statistically significant difference between patients with and without ANP as regards age, gender, and BMI (Appendix B Supplementary Figs. 16–18). A detailed analysis of the results as regards other biomarkers can be found in Appendix B.

**Table 1:** Characteristics of the analyzed study population.

Variable	Value (n = 2387)
Age in years, median (IQR)	57 (44–69)
Male, n (%)	1357 (56.85%)
BMI, median (IQR)	27.14 (23.88–31.25)
**Etiology, n (%)**	
Biliary	955 (40.01%)
Alcoholic	484 (20.28%)
Hypertriglyceridaemia	81 (3.39%)
Biliary and alcoholic	39 (1.63%)
Biliary and hypertriglyceridaemia	13 (0.54%)
Alcoholic and hypertriglyceridaemia	58 (2.43%)
Post-ERCP	67 (2.81%)
Idiopathic	432 (18.10%)
Other	258 (10.81%)
**Revised Atlanta classification**	
Mild, n (%)	1714 (71.81%)
Moderate, n (%)	551 (23.08%)
Severe, n (%)	122 (5.11%)
Mortality, n (%)	66 (2.76%)
Length of stay in days, median (IQR)	8 (6–12)
Patients with local complication, n (%)	623 (26.19%)
APFC, n (%)	510 (21.37%)
Pseudocyst, n (%)	179 (7.50%)
Acute necrotic collection, n (%)	233 (9.76%)
Patients with systemic complication, n (%)	202 (8.46%)
Respiratory failure, n (%)	136 (5.70%)
Heart failure, n (%)	52 (2.18%)
Renal failure, n (%)	83 (3.48%)
New-onset diabetes, n (%)	75 (3.14%)

*APFC* acute peripancreatic fluid collection, *BMI* body mass index, *ERCP* endoscopic retrograde cholangiopancreatography, *IQR* interquartile range.

**Figure 2:**
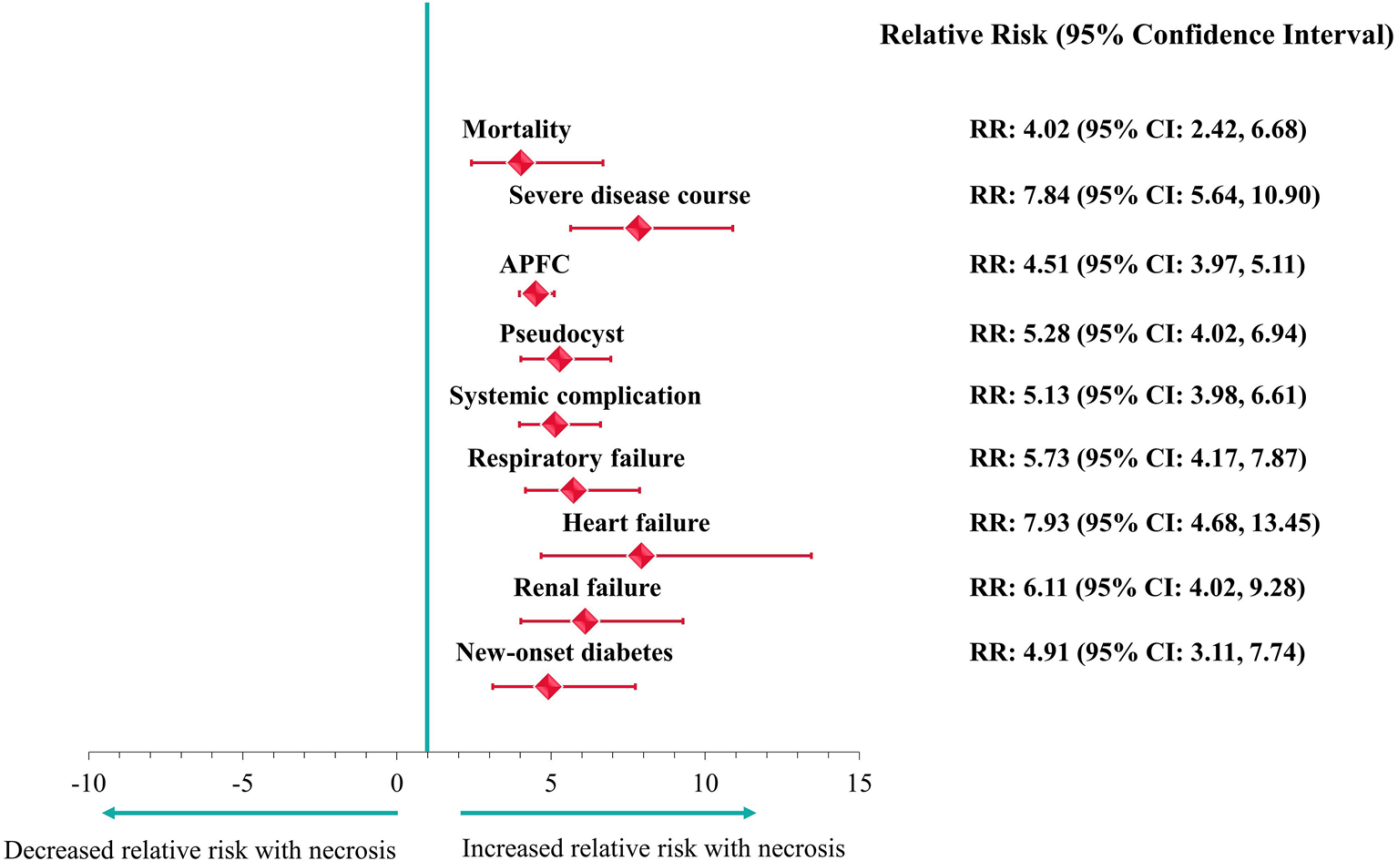
Association between necrosis development and other complications in acute pancreatitis.

ANP was associated with a significantly higher risk for mortality, severe disease course, and all the investigated local and systemic complications (Fig. 2). ANP was also associated with longer hospitalization (9.13 ± 6.21 days vs. 20.78 ± 19.70 days, p < 0.001).

### Model selection and model performance

**Figure 3:**
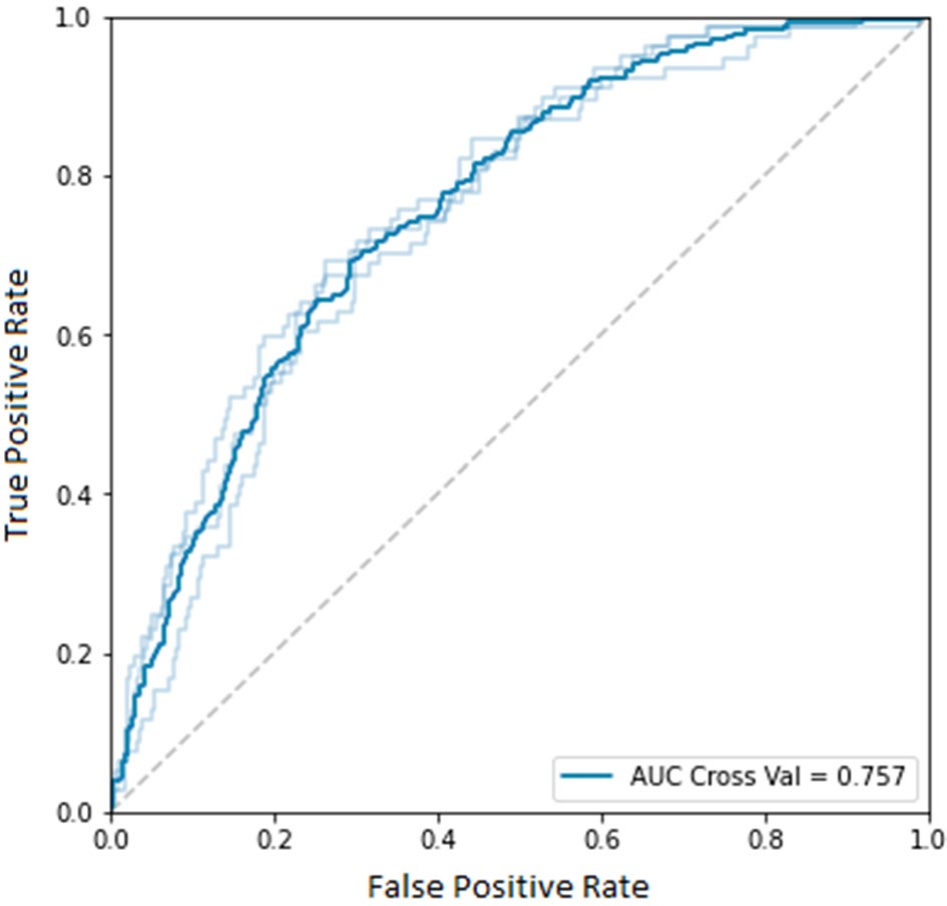
Receiver operating characteristic (ROC) curve for the XGBoost model.

**Figure 4:**
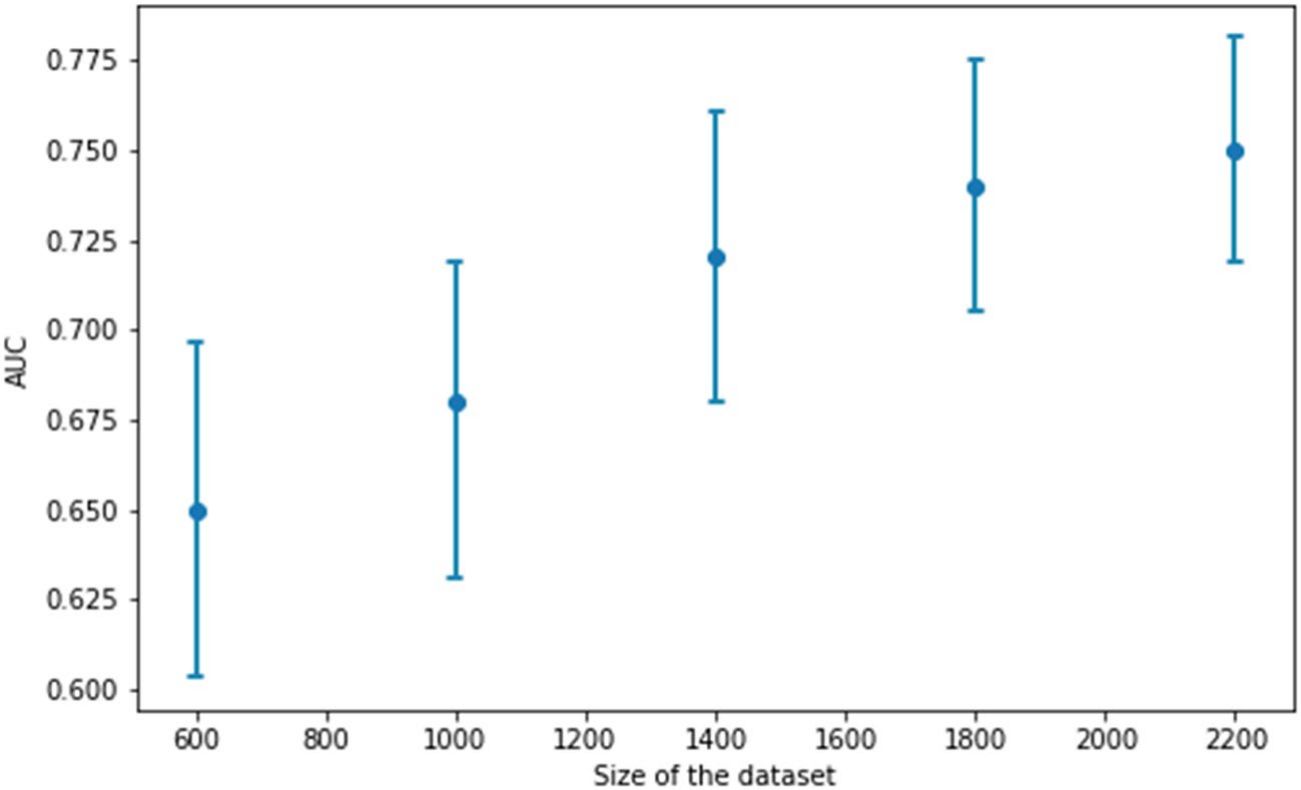
The relationship between the size of the data set and the model performance. The blue dot represents the area under the ROC curve value and the vertical lines show the corresponding confidence intervals.

**Figure 5:**
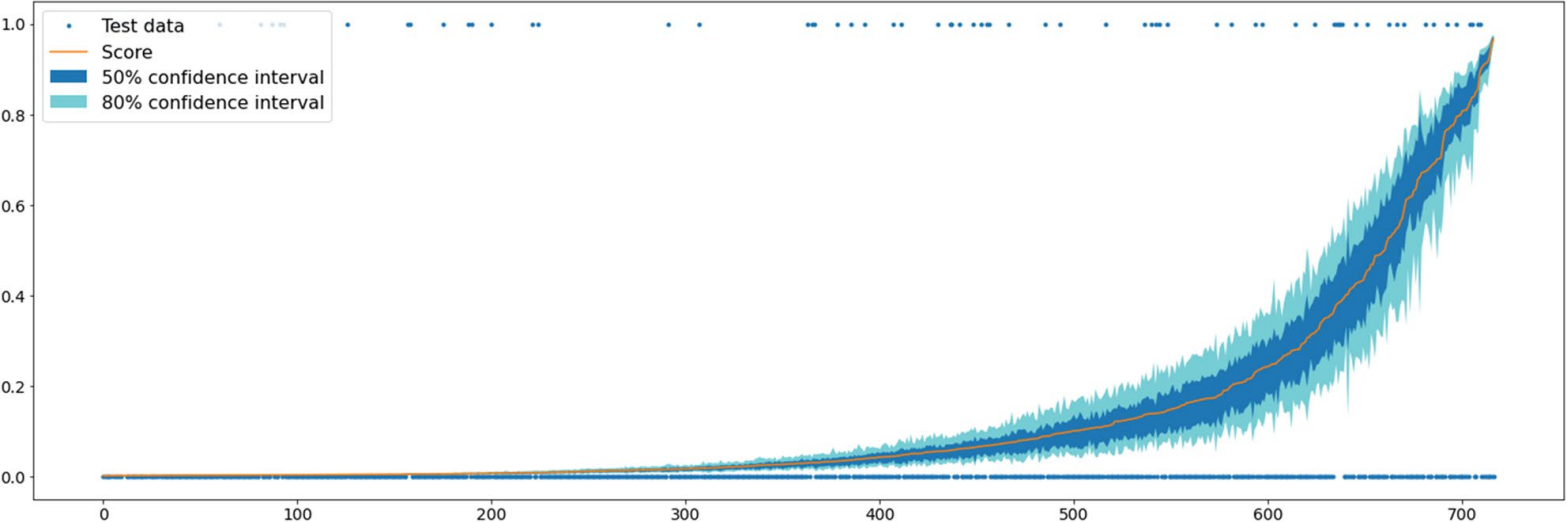
The predicted necrosis probabilities with the corresponding 50% (between the 25th and 50th percentiles) and 80% confidence (between the 10th and 90th percentiles).

After an evaluation of the machine learning algorithms, an XGBoost classifier was identified as the best performing model with an AUC value of 0.757 (standard deviation: 0.012) on cross-validation (Fig. 3). The relationship between the size of the data set and the model performance is depicted in Fig. 4. The steady increase of AUC values implies that our model has not yet reached its maximal prediction performance. Internal validation implies that our model has higher reliability near the endpoints of the prediction spectrum since the confidence intervals are narrower (Fig. 5).

**Figure 6:**
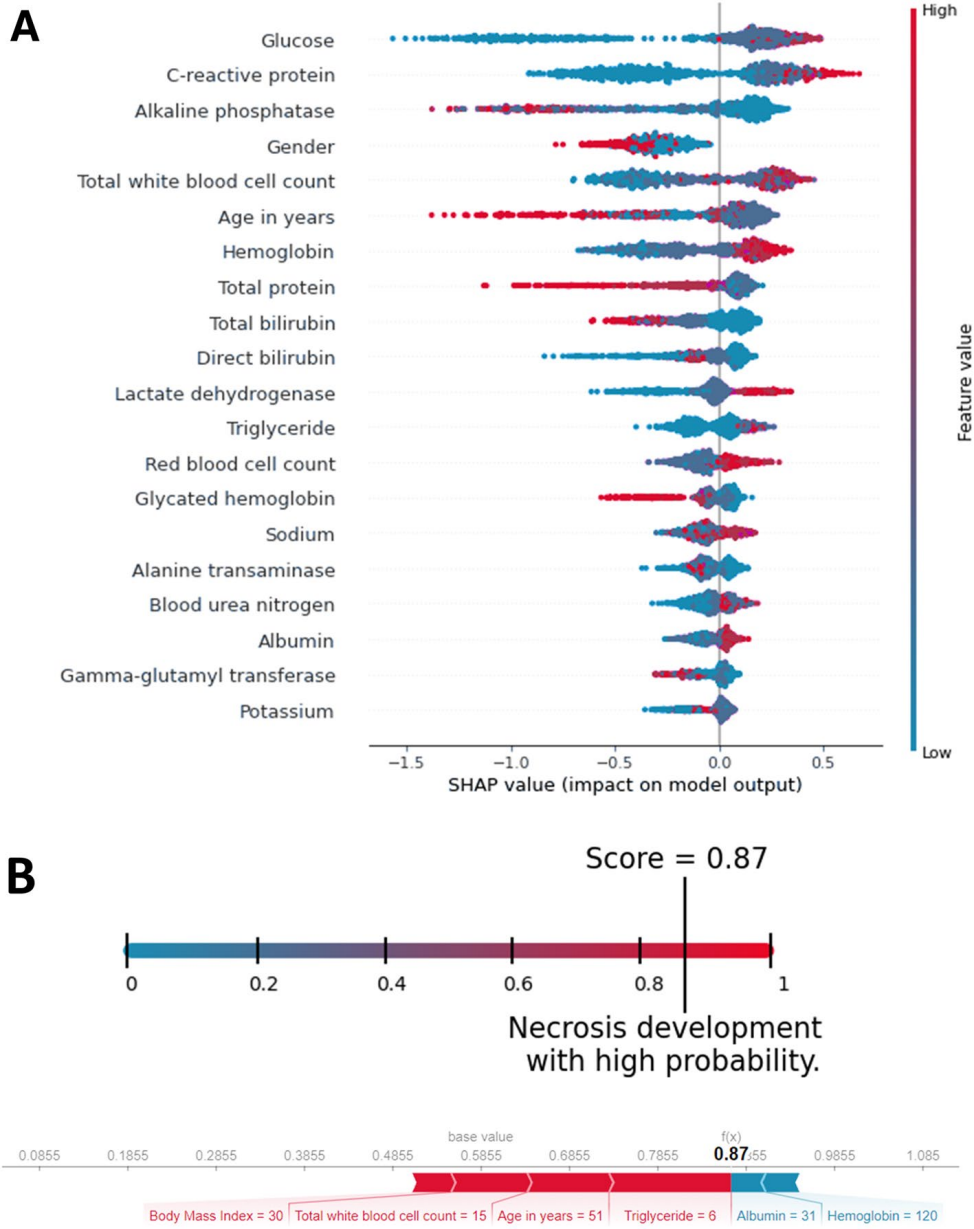
(**A**) The features with the highest impact on model output based on the SHAP values. The higher the predictor is on the list, the bigger the impact on model output. Each patient is represented by a dot. The x-axis represents the extent of the impact on prediction. The color of the dot shows the feature value (e.g. the red color implies higher values). (**B**) An example of prediction and its textual interpretation. The lower picture highlights the effect of individual predictors and the final necrosis probability provided by the model.

**Figure 7:**
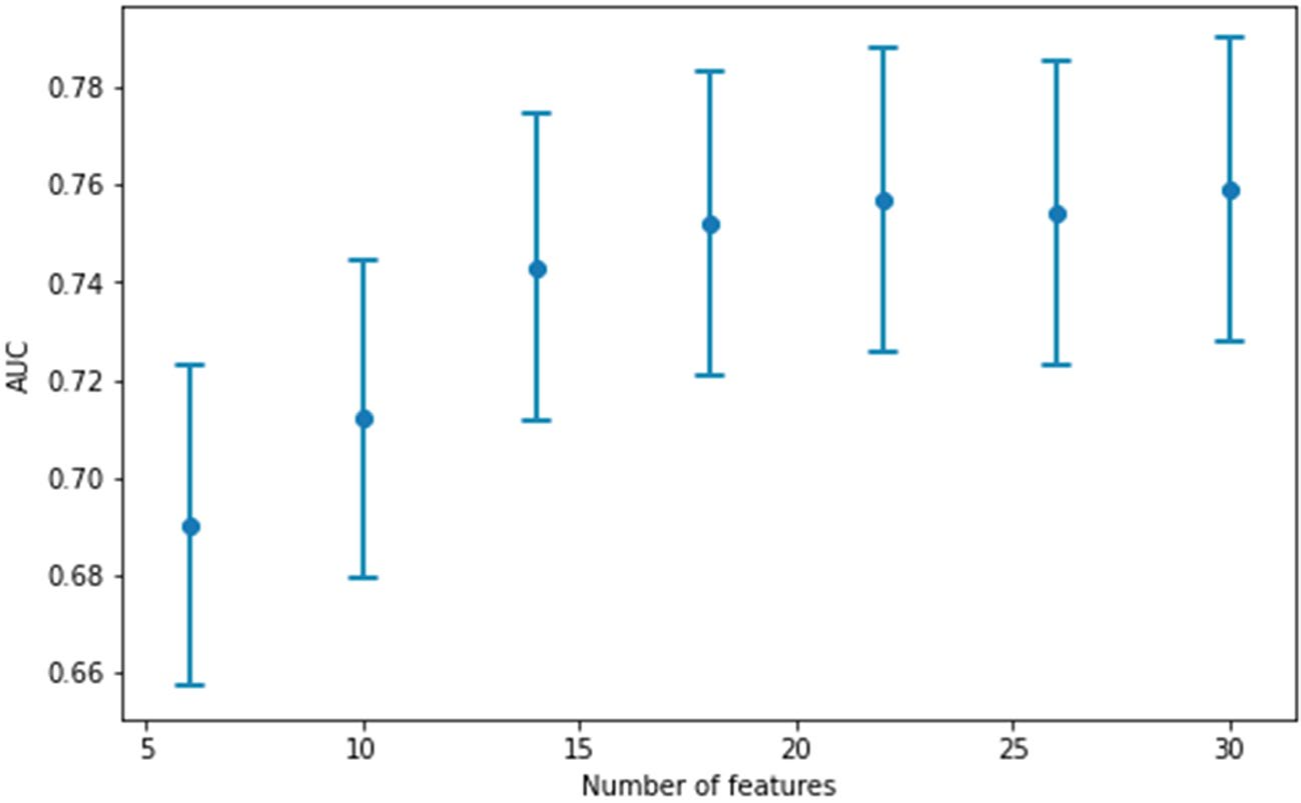
The models build on the *k* predictors with the highest SHAP value.

The assessment of the impact on the model output showed that glucose, CRP, ALP, gender, and WBC have the five highest SHAP values. The most influential predictors are shown in Fig. 6 Panel A. Our assessment showed that the predictive potential depends on the number of biomarkers provided. The models built on the top *k* most influential predictors according to their SHAP values show an increasing performance as regards the predictive potential; however, the extent of this improvement decreases with the number of variables provided (Fig. 7).

### Application

The current version of the model can be accessed at http://necro-app.org/. At least five of the available predictors must be provided to use the application. This limit was applied based on the relation between the size of the dataset and the desired accuracy^30^. The application is aided by a built-in BMI calculator and validations to filter out invalid values. The model offers a numerical probability value between 0 and 1. The higher the number, the higher the risk for ANP becomes. These numerical values are also supplied with a textual interpretation. For educational purposes, the effect of the biomarkers on prediction is also indicated (Fig. 6 Panel B). By checking an extra field, the application assigns a confidence interval in addition to the numerical value. This adds further clarification to the predicted necrosis probability; however, it takes extra time.

## Discussion

The current study describes the first AI model designed to predict ANP. In addition to creating this model, we also implemented it as an easily accessible online tool. In addition to these, ANP was extensively described in a large, prospective, multicenter, cohort study.

### Our cohort in the context of previous data

With the occurrence of ANP in around one-tenth of patients, our results are comparable with previously reported data^31,32^. The importance of ANP in determining the disease course and outcome is well-known^33,34^. Schepers et al. found that 38% of the patients with ANP developed respiratory, cardiovascular or renal system failure^35^. In our cohort, necrosis was also associated with a four to eight-fold increased risk of local and systematic complications, severe disease course, and mortality. We also confirmed their observation regarding prolonged hospitalization indicating the impact of ANP on short-term (i.e.: in-hospital) outcomes. However, the importance of pancreatic necrosis development also lies in the long-term complications.

Recent studies investigated this topic and shed light on long-term outcomes. A meta-analysis of long-term follow-up studies found that the pooled prevalence of exocrine pancreatic insufficiency (EPI) after ANP is between 41 and 58% depending on the extent of necrosis^36^. In a cohort study by Maatman et al., this ratio was only 19%^22^. The discrepancy in the frequency can be attributed to that. While the meta-analysis accounted for EPI during both the hospital stay and follow-up, the cohort assessed EPI after the resolution of AP. Furthermore, the retrospective nature of data has an inherent limitation, which can also explain this difference. In addition to the increased frequency of EPI, they found endocrine insufficiency in 35% of the patients with a median follow-up of 46 months. Despite the fact that our study covered the time of hospitalization, our results imply that necrosis formation increases the risk of new-onset diabetes.

### Currently existing clinical scores as predictors of necrosis

Since ANP is a potent prognostic factor for the short-term severity of AP and could forecast long-term consequences, it would be ideal for identifying these patients as soon as possible. The prediction of ANP was attempted by numerous scoring systems and biomarkers^37^; however, each of them has its own limitations. The Balthazar Computer Tomography Severity Index (CTSI) possesses a higher positive predictive value for necrosis than most commonly used prediction methods^38^, e.g. the Ranson score and the APACHE II score, but it is limited by the availability of CECT. It must be noted that ANP usually becomes apparent after two to three days after disease onset, and that prevents on-admission prediction in certain cases. The application of other scoring systems without mandatory CECT is restricted by their complexity. The Ranson score has eleven factors, which have to be assessed on admission and after 48 hours^39^. The APACHE II score is superior to the scores noted above in terms of flexibility and speed; however, its sensitivity and specificity are far lower^40^.

Two prospective studies compared CTSI, Ranson score, and APACHE II score in predicting necrosis development^41,42^. Despite limitation in terms of patient number and the slightly different AUC values for necrosis, they concluded that the positive predictive value decreases in the following order: CTSI, Ranson score, and APACHE II. It must be emphasized that these scoring systems are strongly limited by the conversion of continuous variables to binary ones and this topic should be investigated by more mathematical models with better accuracy^42^.

### Artificial intelligence in the prognosis of acute pancreatitis

Artificial intelligence has appeared on the scene as a very intriguing modality of data-based decision support, and these models are extensively researched in numerous areas of medicine, including pancreaticobiliary diseases^43^. In the last decade, multiple AI algorithms have been developed in AP^16^. Most of these models were designed to predict the occurrence of a specific complication or disease severity. The most commonly used score in critical care is the APACHE II score; however, three AP severity AI models have been reported to outperform this score ^17–19^. The AI model developed by Keogan et al. was compared to the CTSI and Ranson scores, both of which were found inferior in terms of predicting the severity of AP^44^. It should be noted that this study assessed the disease severity with LOH and not with the revised Atlanta classification. Despite the positive results, these prediction systems, except for the artificial neural network by Mofidi et al.^19^, are limited by the overlap between the data used for model training and the validation. Furthermore, these models need another step after validation. Despite the tremendous efforts and scientific results, much of this knowledge has not been applied in everyday clinical practice^45^. In order to bring these complex models to the bedside, they need to be implemented as easy to use and broadly accessible tools^46^.

The current study was not designed to predict severity but to assess the probability of necrosis formation on clinical admission. Although we had a different outcome, we aimed to overcome the limitations of most previous models and to find a way to use our AI model. As suggested by Shung et al., AI-assisted tools have to overcome many challenges^46^. First of all, we must have high-quality data. This issue was addressed in our study with a four-level data quality check system. The second main challenge is ongoing data maintenance. Our model was constructed such that the new data could be incorporated after validation. Since the predictive potential of the model shows an increasing trend, this could contribute to better accuracy. Algorithmic understanding is also a key factor. The help of physicians, who will eventually use the AI model, is crucial to confirm the performance of such a tool. Furthermore, practitioners could help in differentiating between valid prediction with actual signals and distorted predictions masked by confounding variables^46^. Our web-based application shows the weighted impact of the individual biomarkers in each decision. This tool thus meets these expectations. Consequently, the next step will be screening for these confounding factors while continuously incorporating new data and monitoring the feasibility of the bedside application of this model.

### Strengths and limitations

Our study has multiple strengths and some limitations. Although the predictive potential of this model is similar to that of currently available predictive scoring systems, it has multiple advantages over them. It provides risk assessment with any five of the predictors in our study, which are commonly assessed in daily practice. Therefore, this better reflects everyday clinical practice. To the best of our knowledge, this is the first AI model to strive to predict the development of ANP on clinical admission. We designed our model on a much larger population, as compared to the already existing prognostic AI models in AP, and there was no overlap between the original and validation population. Furthermore, we placed great emphasis on the interpretation of the model for physicians and its implementation by creating an online application. Nevertheless, in addition to predictive model development, ANP was extensively analyzed.

In addition to these strengths, the present study has several limitations. Firstly, as we move further from the endpoint of the prediction spectrum, the confidence of the model becomes wider, and prediction becomes less reliable. Secondly, the cross-validated AUC value of our XGBoost model is currently in the fair range^47^. Thirdly, data imputation can also introduce bias. Most of these limitations can be overcome. Based on our analyses, we could reach better predictive potential by increasing the training sample size and more data could provide more accurate imputation as well. Therefore, by using the application, making further predictions with more data, the model itself could improve.

It should be highlighted that AI models should not be considered as a substitute for human intelligence^16^. These tools, including our model, were designed to facilitate physicians’ decision-making and every prediction should be interpreted in accordance with the clinical picture.

### Implication for practice and research

Development of ANP is associated with several short- and long-term complications, e.g. endocrine insufficiency, but CECT is not performed solely and exclusively to confirm necrosis in AP. Therefore, by knowing the high risk for necrosis development, we can identify a group of patients who need closer follow-up. Nevertheless, this model can aid physicians when CECT is either contraindicated or not available. Also, as soon as new therapies emerge, early identification of ANP will become even more important. Further research is needed on other potential predictive factors, which could be incorporated in the current model to further improve predictions.

## Conclusion

This study is the first to combine prediction of necrosis development and artificial intelligence in AP. The predictive potential of this model is comparable to the already existing clinical scoring systems and the model is expected to further improve with use. The easy-to-use web application supported by the interpretation of the prediction facilitates early, on-admission prediction of necrosis and allows continuous data maintenance and algorithmic understanding.

## Supplementary Information

Supplementary Information.

Supplementary Figures.

## Data Availability

The datasets generated and/or analysed during the current study are available from the corresponding author on reasonable request.
