# Supplementary material for: Early prediction of acute necrotizing pancreatitis by artificial intelligence: a prospective cohort-analysis of 2387 cases
Source: Sci Rep. 2022 May 12;12:7827. doi: 10.1038/s41598-022-11517-w (PMC9098474; doi:10.1038/s41598-022-11517-w)

Appendix A

Early prediction of acute necrotizing pancreatitis by artificial intelligence: A prospective cohort-analysis of 2387 cases

Szabolcs Kiss[1,2,3], József Pintér[4], Roland Molontay[4,5], Marcell Nagy[4], Nelli Farkas[2,6], Zoltán Sipos[2], Péter Fehérvári[2,7], László Pecze[2], Mária Földi[1,2,3], Áron Vincze[8], Tamás Takács[9], László Czakó[9], Ferenc Izbéki[10], Adrienn Halász[1,10], Eszter Boros[10], József Hamvas[11], Márta Varga[12], Artautas Mickevicius[13], Nándor Faluhelyi[14], Orsolya Farkas[14], Szilárd Váncsa[2,15], Rita Nagy[2,3,15], Stefania Bunduc[15,16], Péter Jenő Hegyi[15,17], Katalin Márta[15,17], Katalin Borka[15,18], Attila Doros[15,19], Nóra Hosszúfalusi[15,20], László Zubek[15,21], Bálint Erőss[15,17], Zsolt Molnár[15,21,22], Andrea Párniczky[2,3], Péter Hegyi[2,15,17 #], Andrea Szentesi[1,2,15 #, *], Hungarian Pancreatic Study Group[17,§]

[1]Doctoral School of Clinical Medicine, Faculty of Medicine, University of Szeged, Szeged,
[2]Institute for Translational Medicine, Szentágothai Research Centre, Medical School, University of Pécs, Pécs, Hungary,
[3]Heim Pál National Pediatric Institute, Budapest, Hungary
[4]Human and Social Data Science Lab, Budapest University of Technology and Economics, Budapest, Hungary,
[5]Stochastics Research Group, Hungarian Academy of Sciences - Budapest University of Technology and Economics, Budapest, Hungary,
[6]Institute of Bioanalysis, Medical School, University of Pécs, Pécs, Hungary,
[7]Department of Biomathematics and Informatics, University of Veterinary Medicine, Budapest, Hungary
[8]Division of Gastroenterology, First Department of Medicine, Medical School, University of Pécs, Pécs, Hungary,
[9]Department of Medicine, University of Szeged, Szeged, Hungary,
[10]Department of Internal Medicine, Szent György Teaching Hospital of County Fejér, Székesfehérvár, Hungary,
[11]Bajcsy-Zsilinszky Hospital, Budapest, Hungary,
[12]Department of Gastroenterology, BMKK dr Rethy Pal Hospital, Békéscsaba, Hungary,
[13]Vilnius University Hospital Santaros Clinics, Clinics of Abdominal Surgery, Nephrourology and Gastroenterology, Faculty of Medicine, Vilnius University, Vilnius, Lithuania
[14]Department of Medical Imaging, Medical School, University of Pécs, Pécs, Hungary
[15]Centre for Translational Medicine, Semmelweis University, Budapest, Hungary
[16]Doctoral school, Carol Davila University of Medicine and Pharmacy, Bucharest, Romania
Address: RO-050474 Bulevardul Eroii Sanitari 8., București, Romania
[17]Division of Pancreatic Diseases, Heart and Vascular Center, Semmelweis University, Budapest, Hungary
[18]2nd Department of Pathology, Semmelweis University, Budapest, Hungary
[19]Department of Transplantation and Surgery, Semmelweis University, Budapest, Hungary
[20]Department of Internal Medicine and Hematology, Semmelweis University, Budapest, Hungary
Address: H-1088 Budapest, Szentkirályi u. 46., Hungary
[21]Department of Anaesthesiology and Intensive Therapy, Semmelweis University, Budapest, Hungary
[22]Department of Anaesthesiology and Intensive Therapy, Poznan University of Medical Sciences, Poznan, Poland

# equally contributed

*Correspondence: Andrea Szentesi, Address: Institute for Translational Medicine, Medical School, University of Pécs; 12 Ifjúság u., Pécs, 7624 Hungary; Mobile: +36 (30) 342 1481; E-mail: szentesiai@gmail.com.

[§]A list of authors and their affiliations appears at the end of the paper. A full list of members and contributors of the Hungarian Pancreatic Study Group can be found in the next section.

Hungarian Pancreatic Study Group – list of members and contributors and their affiliations

Szabolcs Kiss[1,2,3], Nelli Farkas[2,6], Zoltán Sipos[2], Péter Fehérvári[2,7], László Pecze[2], Mária Földi[1,2,3], Áron Vincze[8], Tamás Takács[9], László Czakó[9], Ferenc Izbéki[10], Adrienn Halász[1,10], Eszter Boros[10], József Hamvas[11], Márta Varga[12], Artautas Mickevicius[13], Nándor Faluhelyi[14], Orsolya Farkas[14], Szilárd Váncsa[2,15], Rita Nagy[2,3,15], Stefania Bunduc[15,16], Péter Jenő Hegyi[15,17], Katalin Márta[15,17], Katalin Borka[15,18], Attila Doros[15,19], Nóra Hosszúfalusi[15,20], László Zubek[15,21] Bálint Erőss[15,17], Zsolt Molnár[15,21,22], Andrea Párniczky[2,3], Péter Hegyi[2,15,17 #], Andrea Szentesi[1,2,15 #] Judit Bajor[8], Szilárd Gódi[8], Patrícia Sarlós[8], József Czimmer[8], Imre Szabó[8], Gabriella Pár[8], Anita Illés[8], Roland Hágendorn[8], Balázs Csaba Németh[9], Balázs Kui[9], Dóra Illés[9], László Gajdán[10], Veronika Dunás-Varga[10], Roland Fejes[10], Mária Papp[23], Zsuzsanna Vitális[23], János Novák[24], Imola Török[25], Melania Macarie[25], Elena Ramírez-Maldonado[26], Ville Sallinen[27], Shamil Galeev[28], Barnabás Bod[29], Ali Tüzün Ince[30], Dániel Pécsi[2], Péter Varjú[2], Márk Félix Juhász[2,3], Klementina Ocskay[2,15], Alexandra Mikó[2,27], and Zsolt Szakács[2,32], Zsolt Szentkereszty[33], Hunor Farkas[25], Petr Pencik[34], Goran Poropat[35], Davor Stimac[35], Imanta Ozola-Zalite[36], Andrey Litvin[37], Árpád Patai[38], Kristina Zadorozhna[39], János Sümegi[40], Árpád V. Patai[41], István Hritz[41], Csaba Góg[42], Masayasu Horibe[43], and Georgiana Robu[44]

[1]Doctoral School of Clinical Medicine, Faculty of Medicine, University of Szeged, Szeged, [2]Institute for Translational Medicine, Szentágothai Research Centre, Medical School, University of Pécs, Pécs, Hungary, [3]Heim Pál National Pediatric Institute, Budapest, Hungary, [6]Institute of Bioanalysis, Medical School, University of Pécs, Pécs, Hungary, [7]Department of Biomathematics and Informatics, University of Veterinary Medicine, Budapest, Hungary, [8]Division of Gastroenterology, First Department of Medicine, Medical School, University of Pécs, Pécs, Hungary, [9]Department of Medicine, University of Szeged, Szeged, Hungary, [10]Department of Internal Medicine, Szent György Teaching Hospital of County Fejér, Székesfehérvár, Hungary, [11]Bajcsy-Zsilinszky Hospital, Budapest, Hungary, [12]Department of Gastroenterology, BMKK dr Rethy Pal Hospital, Békéscsaba, Hungary, [13]Vilnius University Hospital Santaros Clinics, Clinics of Abdominal Surgery, Nephrourology and Gastroenterology, Faculty of Medicine, Vilnius University, Vilnius, Lithuania, [14]Department of Medical Imaging, Medical School, University of Pécs, Pécs, Hungary, [15]Centre for Translational Medicine, Semmelweis University, Budapest, Hungary, [16]Doctoral school, Carol Davila University of Medicine and Pharmacy, Bucharest, Romania, [17]Division of Pancreatic Diseases, Heart and Vascular Center, Semmelweis University, Budapest, Hungary, [18]2nd Department of Pathology, Semmelweis University, Budapest, Hungary, [19]Department of Transplantation and Surgery, Semmelweis University, Budapest, Hungary, [20]Department of Internal Medicine and Hematology, Semmelweis University, Budapest, Hungary, [21]Department of Anaesthesiology and Intensive Therapy, Semmelweis University, Budapest, Hungary, [22]Department of Anaesthesiology and Intensive Therapy, Poznan University of Medical Sciences, Poznan, Poland, [23]Division of Gastroenterology, Department of Internal Medicine, University of Debrecen, Debrecen, Hungary, [24]Pándy Kálmán Hospital of Békés County, Gyula, Hungary, [25]County Emergency Clinical Hospital of Târgu Mures - Gastroenterology Clinic and University of Medicine, Pharmacy, Sciences and Technology ”George Emil Palade”, Targu Mures, Romania, [26]General Surgery, Consorci Sanitari del Garraf, Sant Pere de Ribes, Barcelona, Spain, [27]Department of Transplantation and Liver Surgery, Helsinki University Hospital and University of Helsinki, Helsinki, Finland, [28]Saint Luke Clinical Hospital, St. Petersburg, Russia, [29]Dr. Bugyi István Hospital, Szentes, Hungary, [30]Hospital of Bezmialem Vakif University, School of Medicine, Istanbul, Turkey, [31]Department of Medical Genetics, Medical School, University of Pécs, Pécs, [32]First Department of Medicine, Medical School, University of Pécs, Pécs, Hungary, [33]Institute of Surgery, University of Debrecen, Debrecen, Hungary, [25]County Emergency Clinical Hospital of Târgu Mures - Gastroenterology Clinic and University of Medicine, Pharmacy, Sciences and Technology ”George Emil Palade”, Targu Mures, Romania, [34]Centrum péče o zažívací trakt, Vítkovická nemocnice a.s., Ostrava, Czech Republic, [35]Clinical Hospital Center Rijeka, Rijeka, Croatia, [36]Gastroenterology, Hepatology and Nutritional Centre, Pauls Stradins Clinical University Hospital, Riga, Latvia, [37]Immanuel Kant Baltic Federal University, Kaliningrad, Russia, Gomel Regional Clinical Hospital, Gomel, Belarus, [38]Markusovszky University Teaching Hospital, Szombathely, Hungary, [39]Bogomolets National Medical University, Kiev, Ukraine, [40]Borsod-Abaúj-Zemplén County Hospital and University Teaching Hospital, Miskolc, Hungary, [41]Department of Surgery, Transplantation and Gastroenterology, Semmelweis University, Budapest, Hungary [42]Healthcare Center of County Csongrád, Makó, Hungary, [43]Division of Gastroenterology and Hepatology, Department of Internal Medicine, Keio University School of Medicine, Tokyo, Japan, [44]Central Military Emergency Hospital "Dr Carol Davila", Bucharest, Romania

Supplementary Figure 1: Geographical distribution of patients in the whole cohort

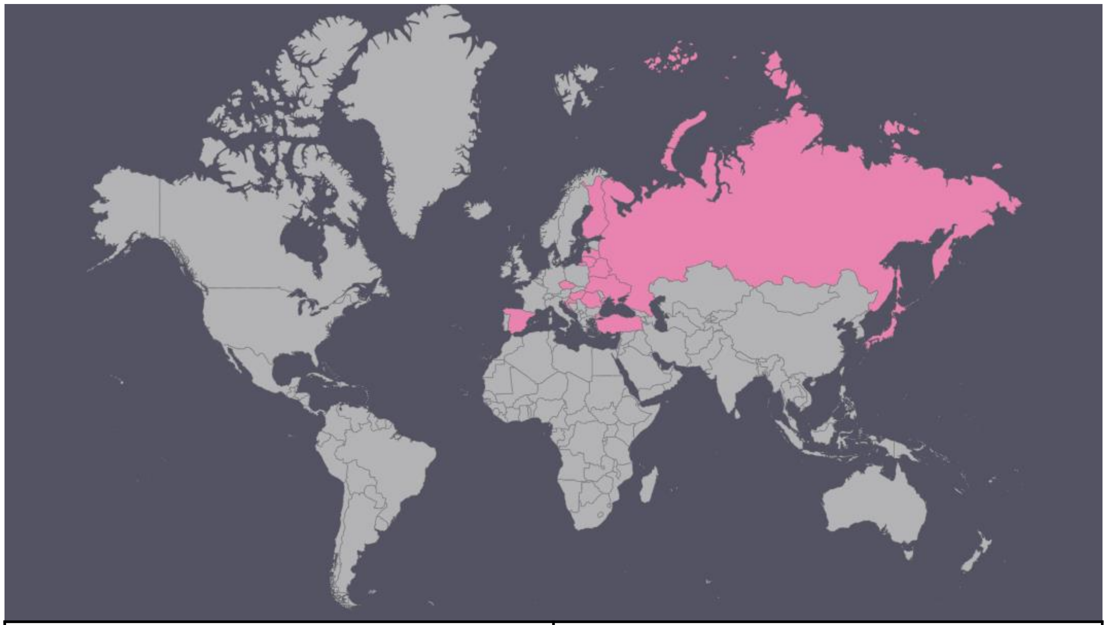

Country	Number of patients
Belarus	8
Croatia	11
Czech Republic	11
Finland	25
Hungary	2225
Japan	2
Latvia	6
Lithuania	31
Romania	58
Russia	28
Spain	28
Turkey	20
Ukraine	8

Supplementary Table 1: List of study centres

	Centre		Centre
1	Bács-Kiskun County Hospital, Kecskemét, Hungary	16	Dr. Réthy Pál Hospital, Békéscsaba, Hungary
2	Bajcsy-Zsilinszky Hospital and Clinic, Budapest, Hungary	17	First Department of Medicine, Medical School, University of Pécs, Pécs, Hungary
3	Bogomolets National Medical University, Kiev, Ukraine	18	Gastroenterology, Hepatology and Nutritional Centre, Pauls Stradins Clinical University Hospital, Riga, Latvia
4	Borsod-Abaúj-Zemplén County Hospital and University Teaching Hospital, Miskolc, Hungary	19	General Surgery, Consorci Sanitari del Garraf, Sant Pere de Ribes, Barcelona, Spain
5	Buda Hospital of the Hospitaller Order of Saint John of God, Budapest, Hungary	20	Gomel Regional Clinical Hospital, Gomel, Belarus
6	Central Military Emergency Hospital "Dr Carol Davila", Bucharest, Romania	21	Heim Pál National Pediatric Institute, Budapest, Hungary
7	Centrum péče o zažívací trakt, Vítkovická nemocnice a.s., Ostrava, Czech Republic	22	Hospital of Bezmialem Vakif University, School of Medicine, Istanbul, Turkey
8	Clinical Hospital Center Rijeka, Rijeka, Croatia	23	Keio University, Tokyo, Japan
9	County Emergency Clinical Hospital of Targu Mures Hospital, University of Medicine, Pharmacy, Sciences and Technology of Targu Mures, Romania	24	Markusovszky University Teaching Hospital, Szombathely, Hungary
10	Csongrád County Health Center, Makó, Hungary	25	Military Hospital, Budapest, Hungary
11	Department of Internal Medicine, University of Debrecen, Debrecen, Hungary	26	Pándy Kálmán Hospital of Békés County, Gyula, Hungary
12	Department of Internal Medicine, University of Szeged, Szeged, Hungary	27	Saint Luke Clinical Hospital, St. Petersburg, Russia
13	Department of Surgery, University of Debrecen	28	Second Department of Internal Medicine, Semmelweis University, Budapest, Hungary
14	Department of Transplantation and Liver Surgery, Helsinki University Hospital and University of Helsinki, Helsinki, Finland	29	Szent György University Teaching Hospital of Fejér County, Székesfehérvár, Hungary
15	Dr. Bugyi István Hospital, Szentes, Hungary	30	Vilnius University Hospital, Vilnius, Lithuania

Supplementary Table 2: Data quality in the analyzed population regarding epidemiology, etiology, and disease outcomes

**EPIDEMIOLOGY, ETIOLOGY**	**OVERALL**	**UPLOADED DATA**	**%**
Age	2387	2387	100%
Gender	2387	2387	100%
Etiology	2387	2387	100%
Total	***7161***	***7161***	***100%***

**OUTCOMES**	**OVERALL**	**UPLOADED DATA**	**%**
Local pancreatic complications	2387	2379	99.7%
Acute peripancreatic fluid collection	2387	2380	99.7%
Pancreatic pseudocyst	2387	2380	99.7%
Pancreatic necrosis	2387	2387	100%
Diabetes mellitus as complication	2387	2387	100%
Systemic complication	2387	2379	99.7%
Renal failure	2387	2379	99.7%
Heart failure	2387	2379	99.7%
Respiratory failure	2387	2378	99.6%
Length of hospitalization	2387	2387	100%
Severity (mild/moderate/severe)	2387	2387	100%
Mortality	2387	2387	100%
Total	***28644***	***28589***	***99.8%***

Supplementary Table 3: Data quality in the analyzed population regarding laboratory parameters

LABORATORY PARAMETERS ON ADMISSION	OVERALL	UPLOADED DATA	%
Amylase	2387	2332	97.7%
Lipase	2387	1916	80.3%
Triglyceride (TG)	2387	1377	57.7%
Total cholesterol	2387	1257	52.7%
C-reactive protein (CRP)	2387	2223	93.1%
Procalcitonin (PCT)	2387	889	37.2%
Total white blood cell count (WBC)	2387	2322	97.3%
Red blood cell count (RBC)	2387	1901	79.6%
Hematocrit	2387	1908	79.9%
Hemoglobin	2387	1884	78.9%
Thrombocyte	2387	1902	79.7%
Glucose	2387	2155	90.3%
Glycated hemoglobin (HbA1c)	2387	729	30.5%
Aspartate transaminase (ASAT)	2387	1730	72.5%
Alanine transaminase (ALAT)	2387	1692	70.9%
Gamma-glutamyl transferase (γGT)	2387	2106	88.2%
Alkaline phosphatase (ALP)	2387	2109	88.4%
Lactate dehydrogenase (LDH)	2387	1650	69.1%
Total bilirubin	2387	2159	90.4%
Direct bilirubin	2387	1205	50.5%
Potassium	2387	1825	76.5%
Sodium	2387	1817	76.1%
Calcium	2387	1552	65.0%
Albumin	2387	1059	44.4%
Total protein	2387	900	37.7%
Estimated glomerular filtration rate (eGFR)	2387	2190	91.7%
Creatinine	2387	2234	93.6%
Blood urea nitrogen (BUN)	2387	2181	91.4%
Total	***66836***	***49204***	***73.6%***

Supplementary Figure 2: Representativity analysis regarding age distribution showed no difference between the whole cohort and the analyzed population (t-test, p=0.738)

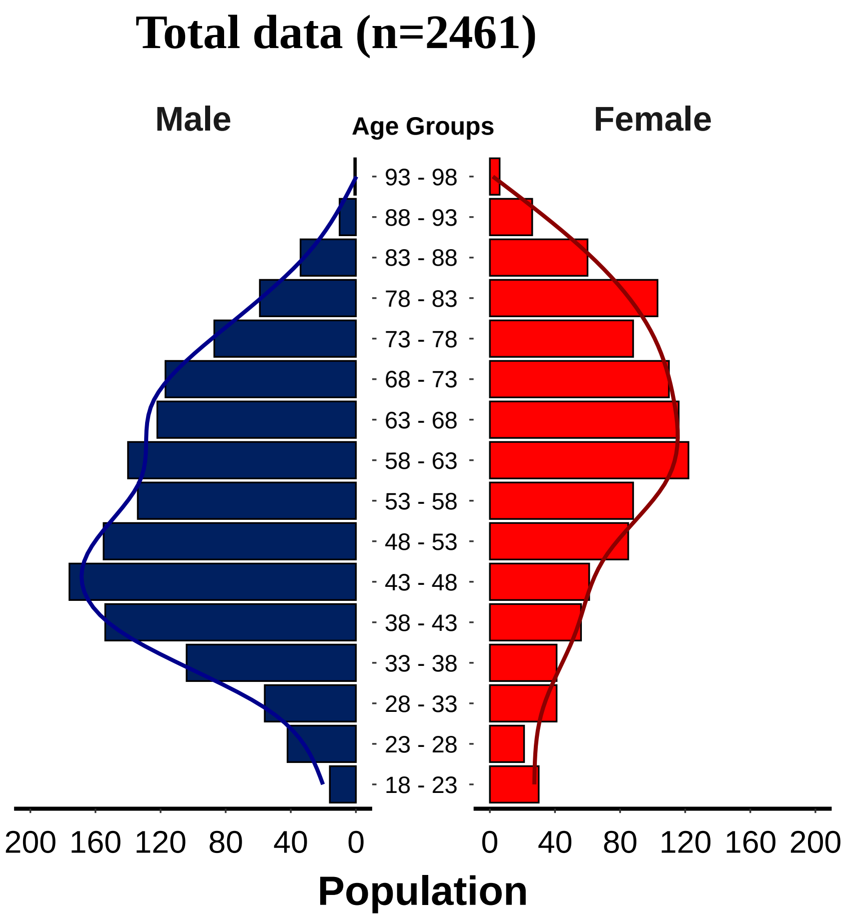

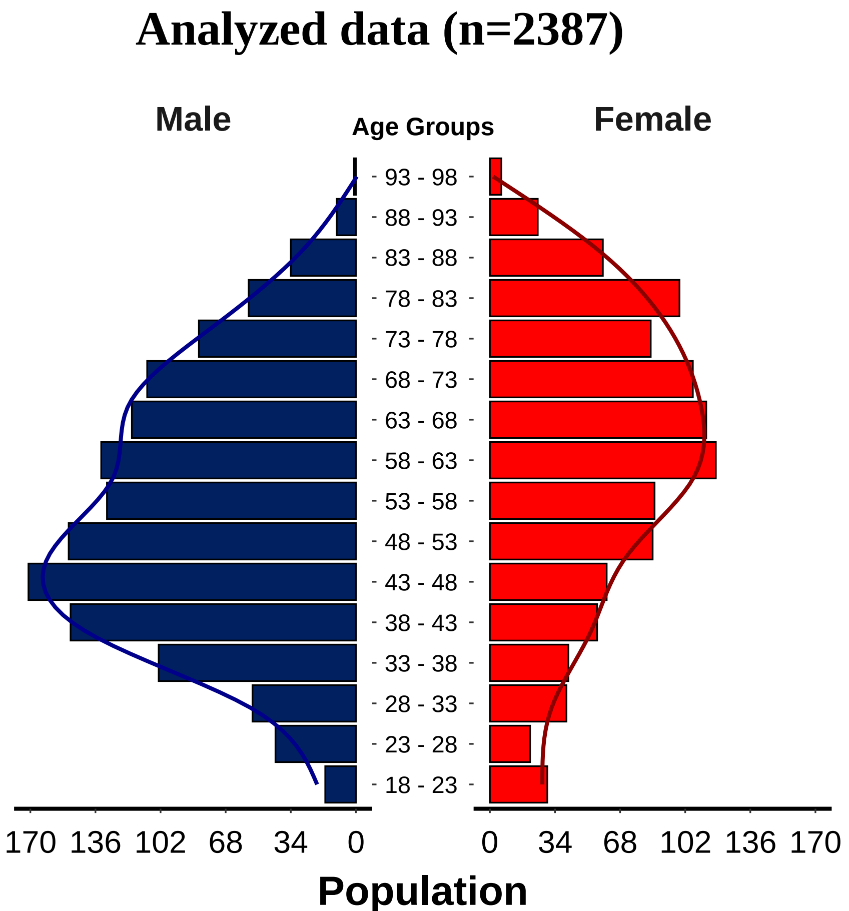

Supplementary Figure 3: Representativity analysis regarding gender distribution showed no difference between the whole cohort and the analyzed population (Chi^2 test, p=0.750)

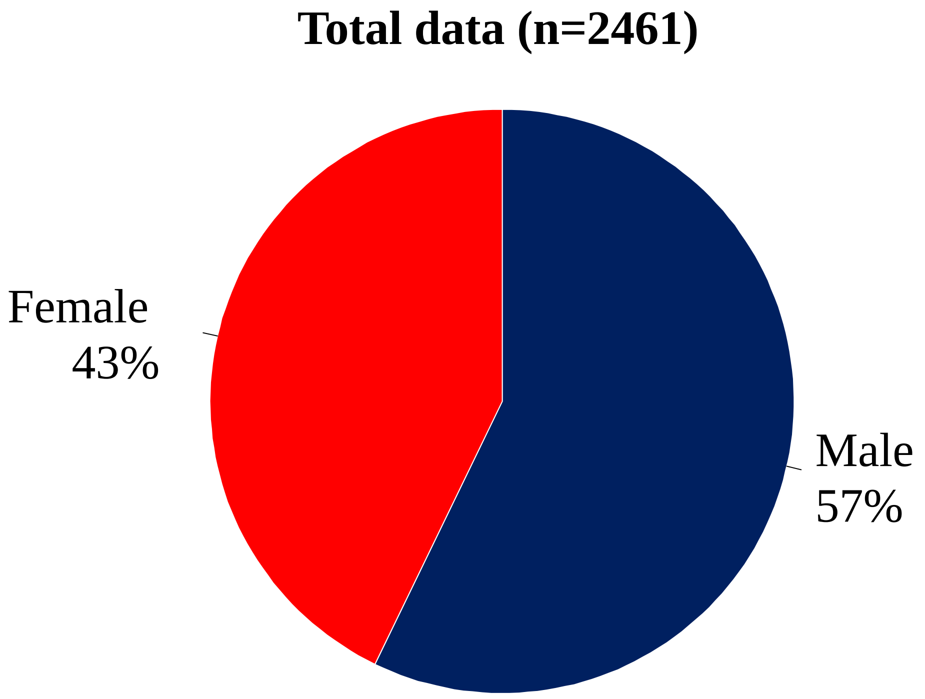

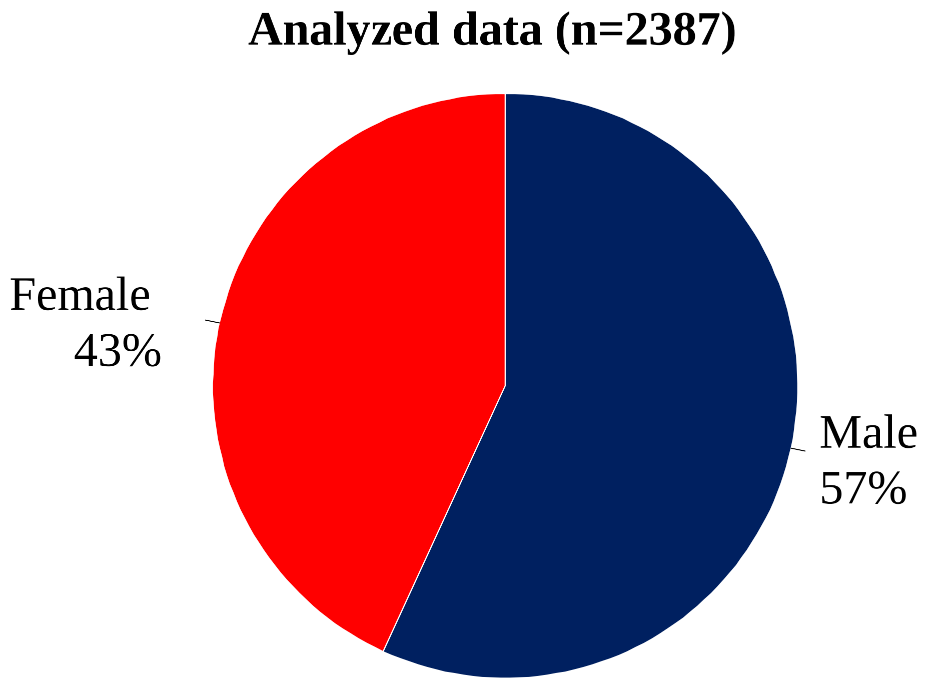

Supplementary Figure 4: Representativity analysis regarding disease severity showed no difference between the whole cohort and the analyzed population (Chi^2 test, p=0.305)

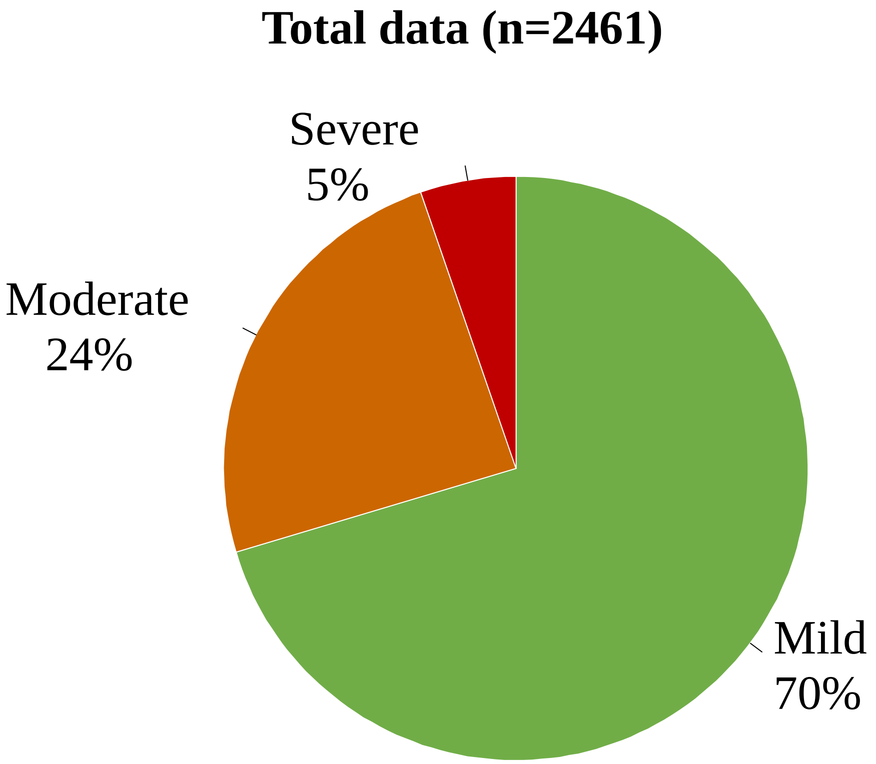

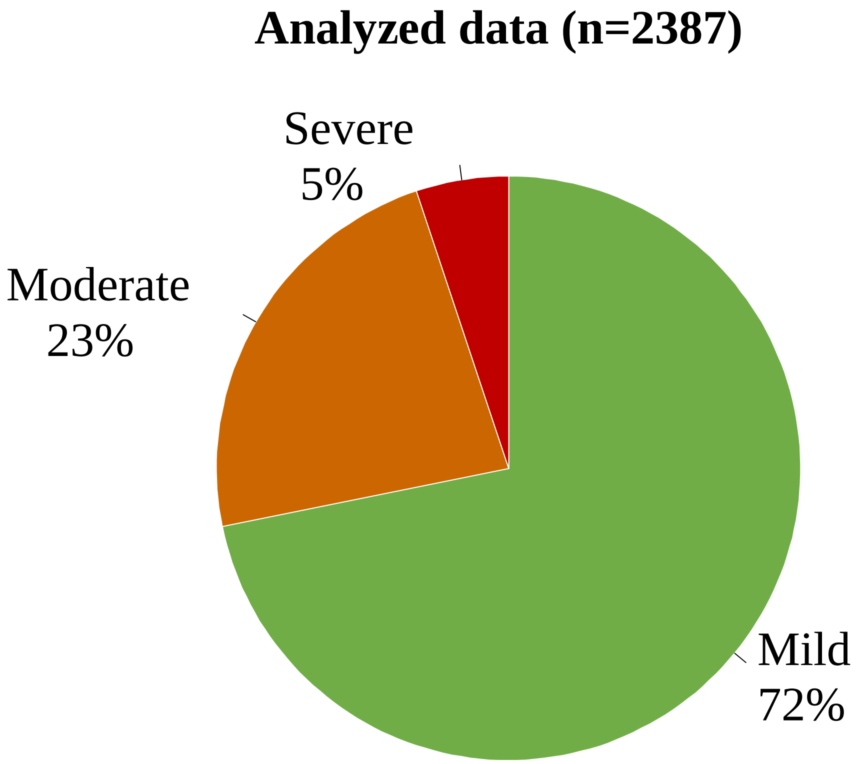

Supplementary Figure 5: Representativity analysis regarding mortality showed no difference between the whole cohort and the analyzed population (Chi^2 test, p=0.641)

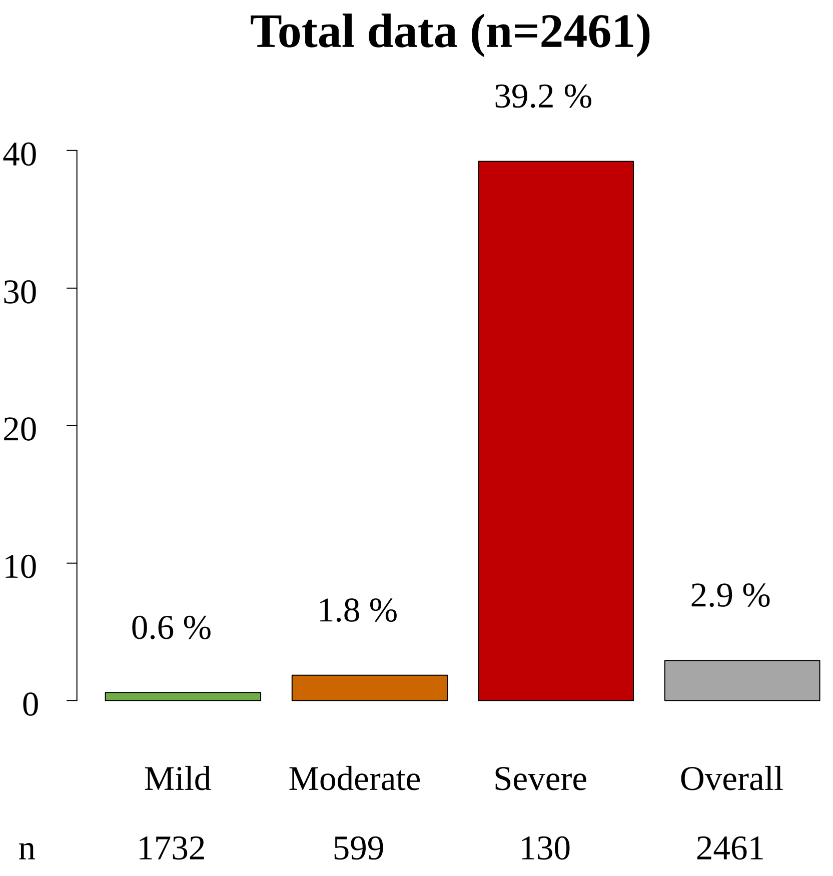

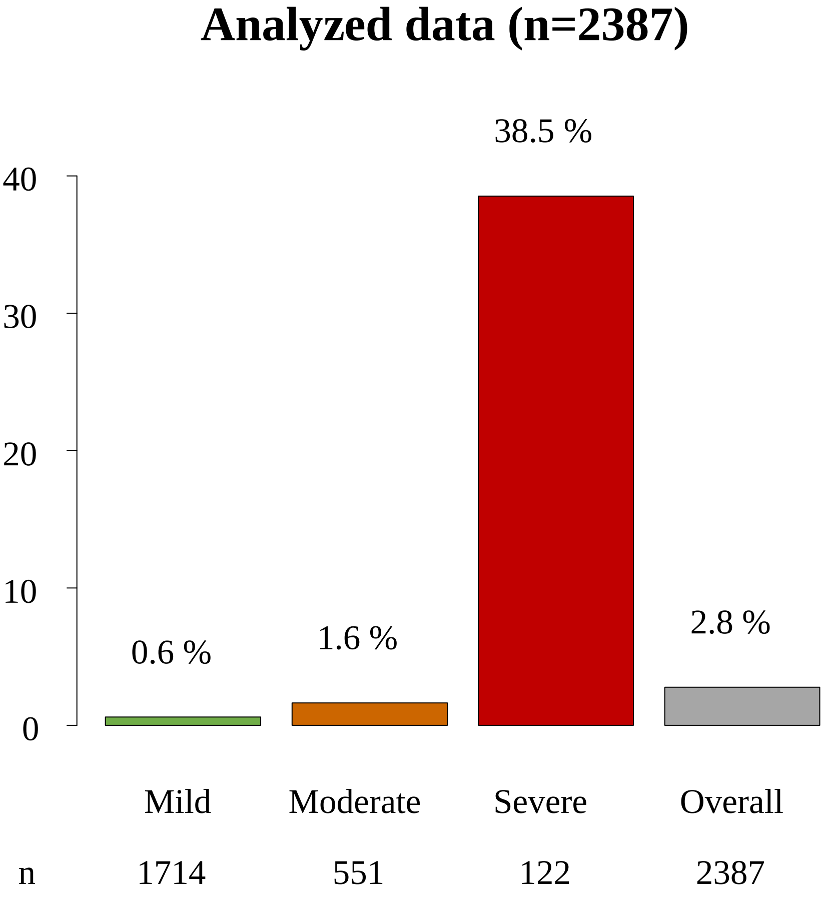

Supplementary Figure 6: Representativity analysis regarding length of hospitalization showed no difference between the whole cohort and the analyzed population (Mann-Whitney test, p=0.641)

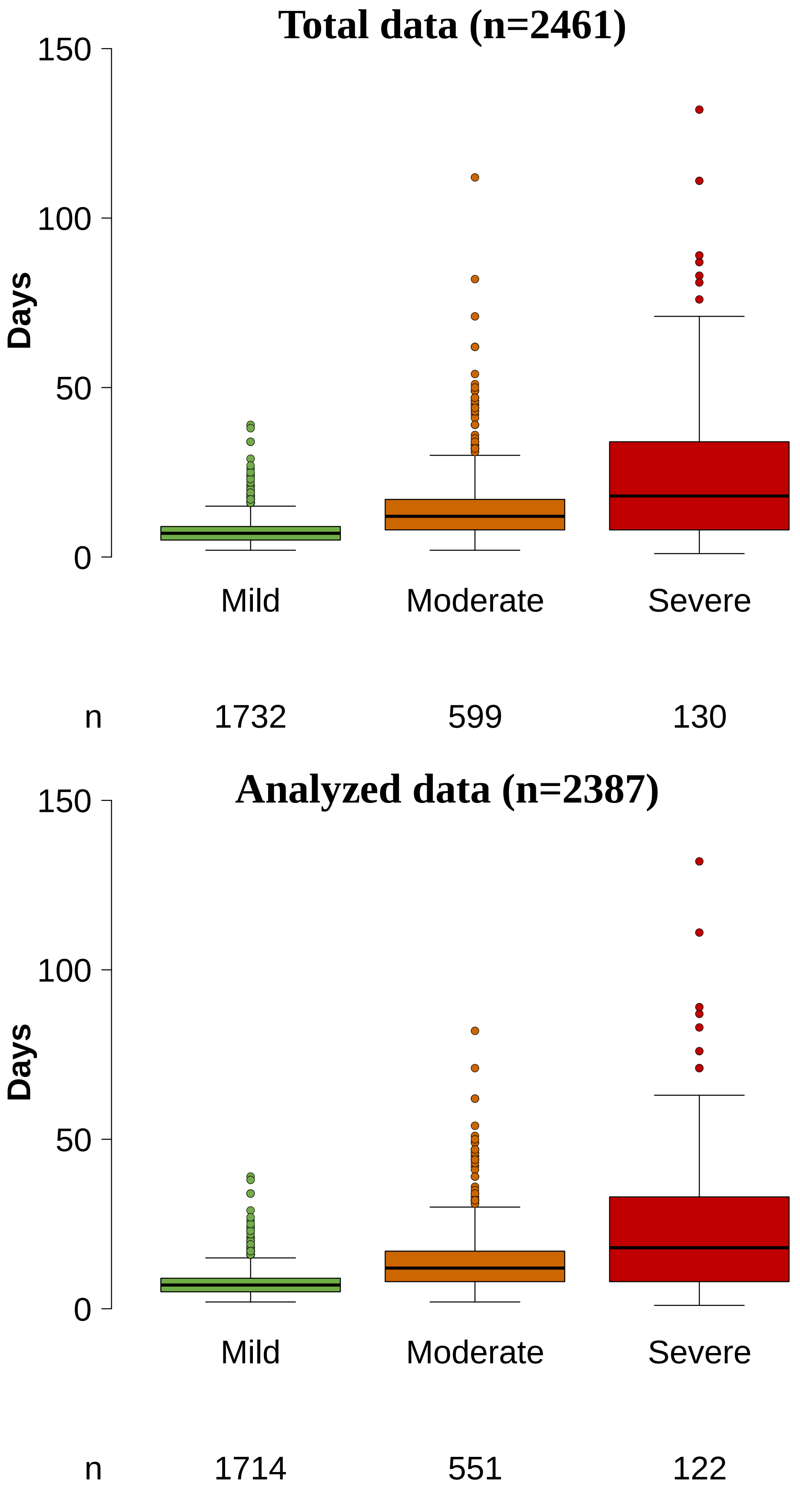

Supplementary Figure 7: Representativity analysis regarding local pancreatic complications showed no difference between the whole cohort and the analyzed population (Chi^2 test, p=0.096)

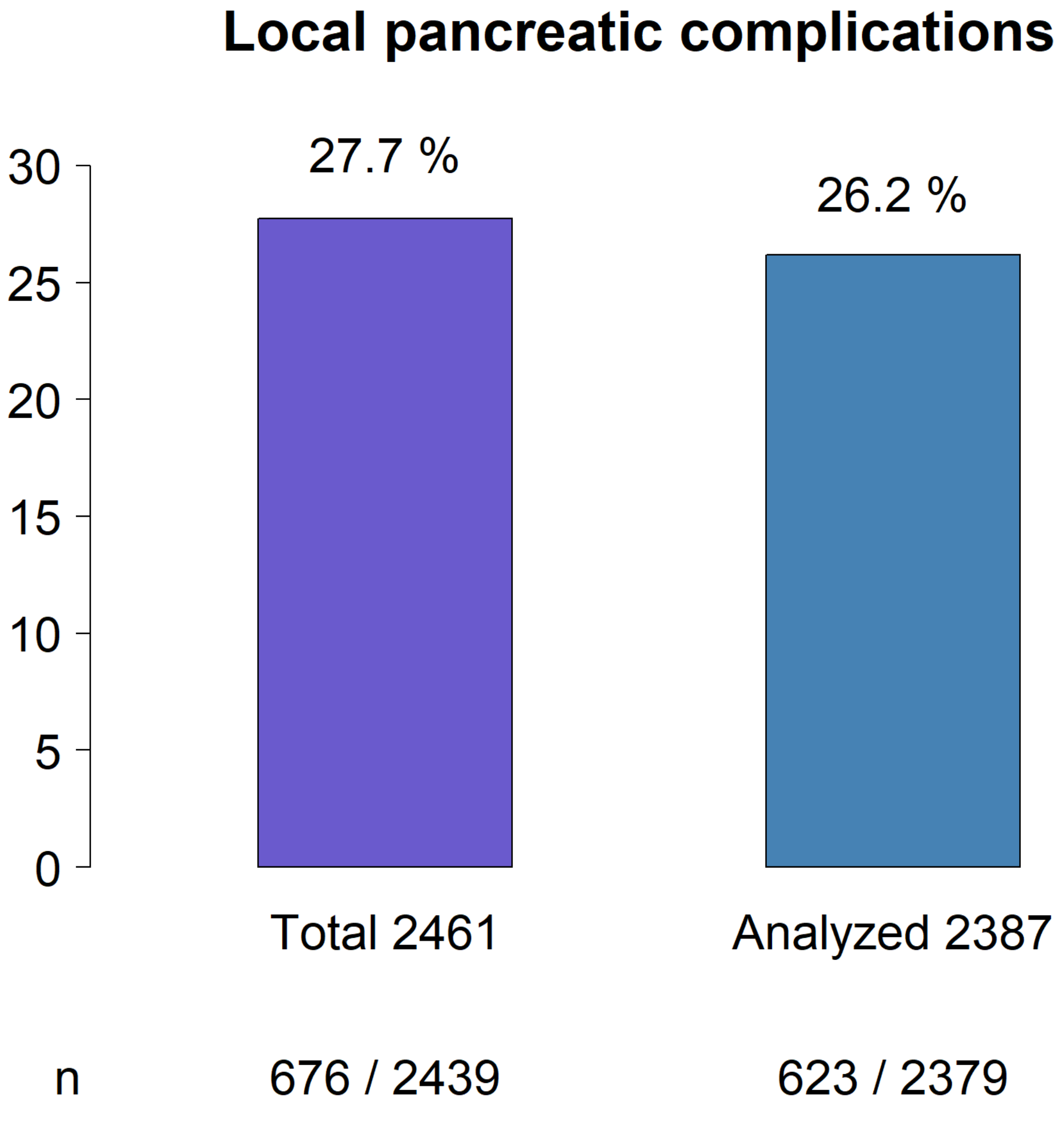

Supplementary Figure 8: Representativity analysis regarding acute peripancreatic fluid collection showed no difference between the whole cohort and the analyzed population (Chi^2 test, p=0.194)

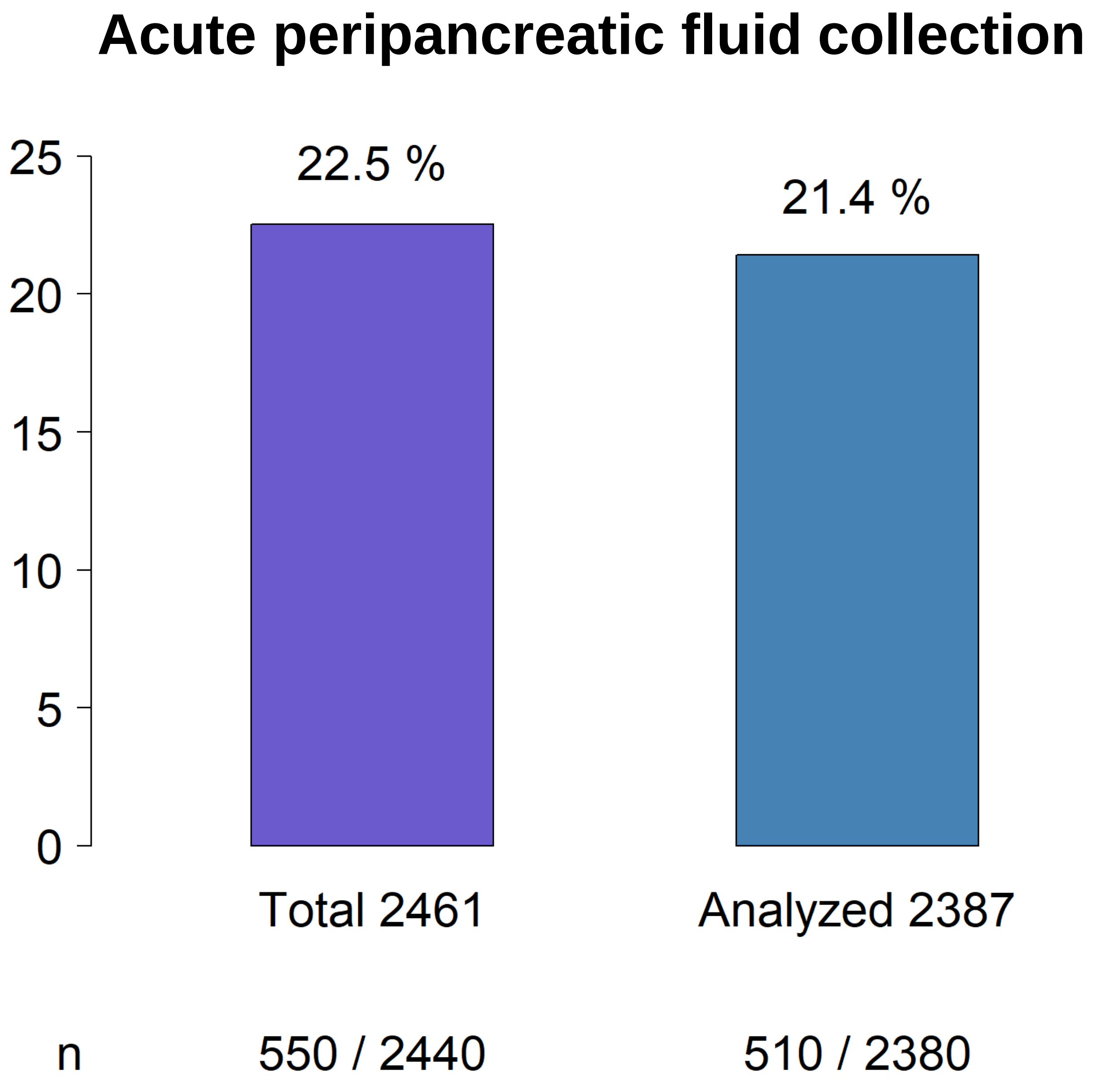

Supplementary Figure 9: Representativity analysis regarding pseudocyst showed no difference between the whole cohort and the analyzed population (Chi^2 test, p=0.397)

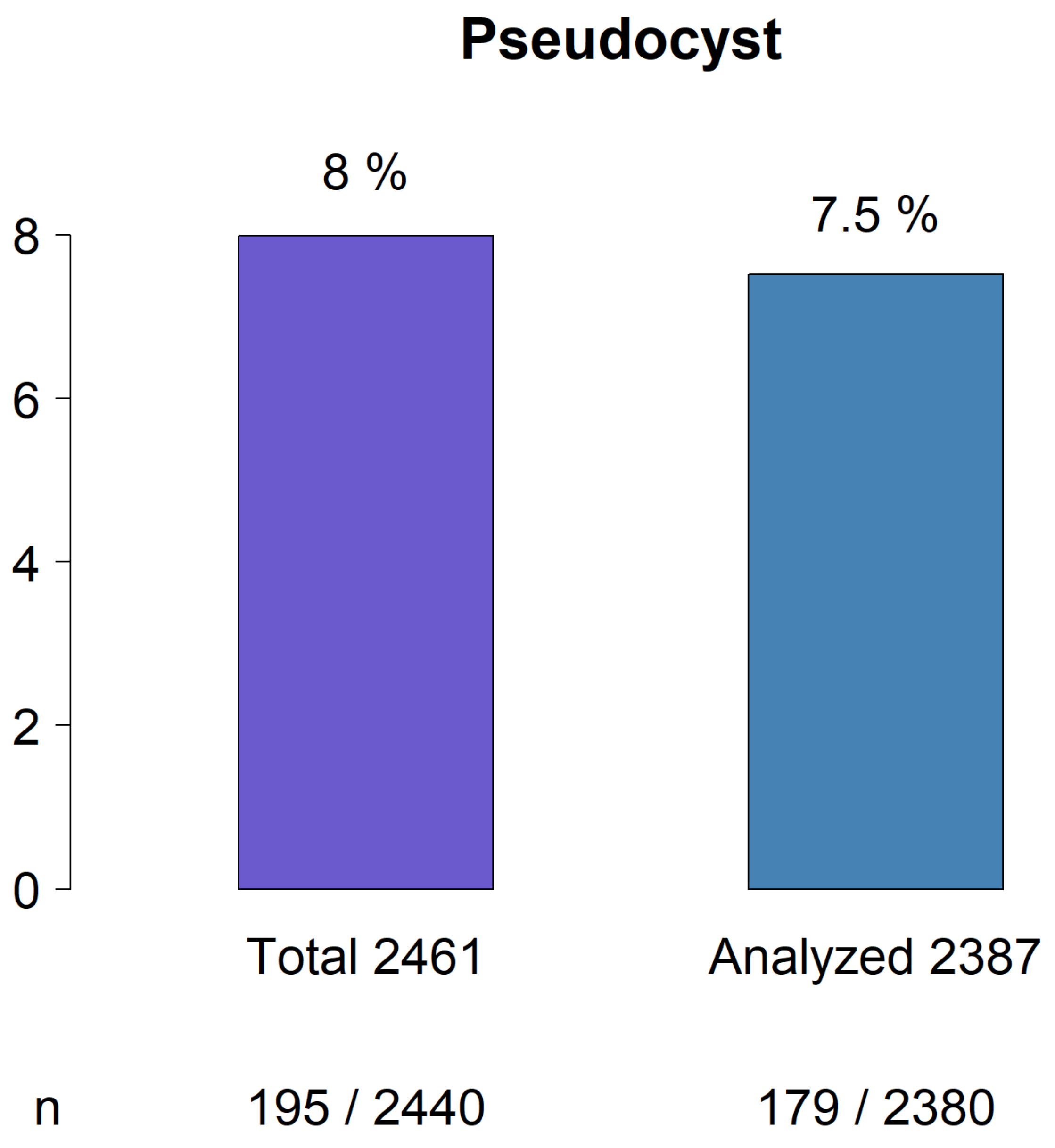

Supplementary Figure 10: Representativity analysis regarding new-onset diabetes showed no difference between the whole cohort and the analyzed population (Chi^2 test, p=0.970)

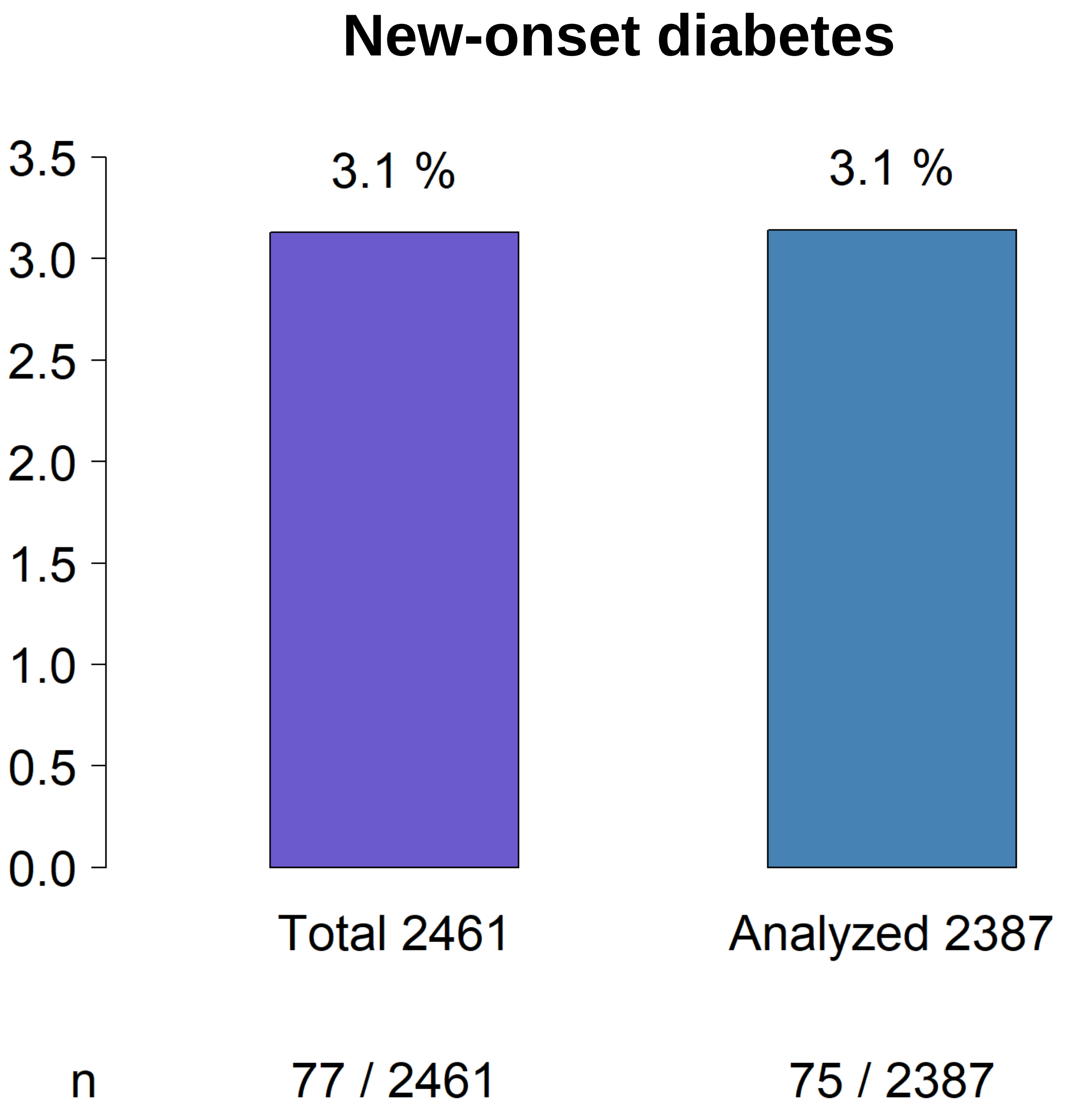

Supplementary Figure 11: Representativity analysis regarding systemic complications showed no difference between the whole cohort and the analyzed population (Chi^2 test, p=0.739)

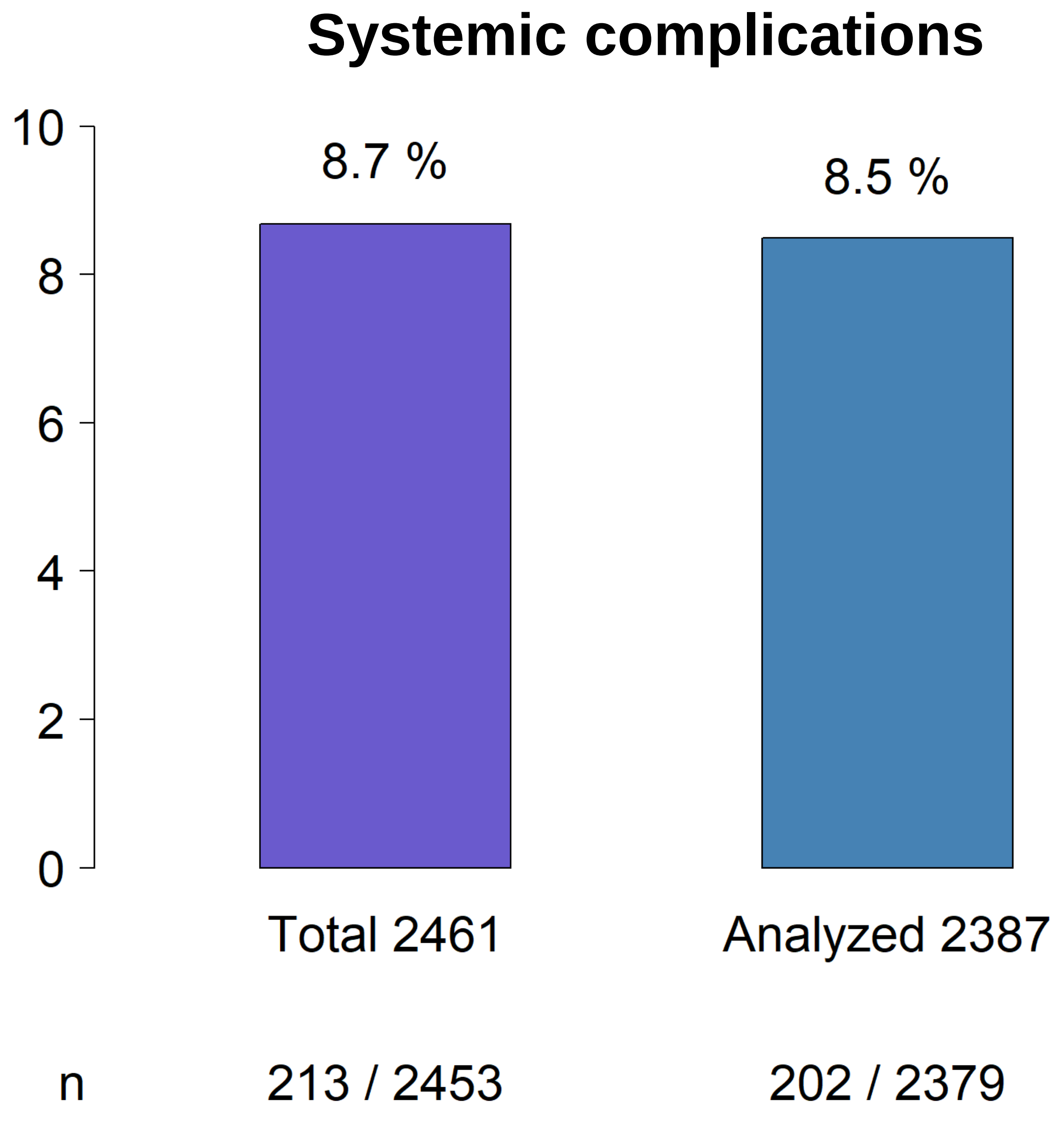

Supplementary Figure 12: Representativity analysis regarding respiratory failure showed no difference between the whole cohort and the analyzed population (Chi^2 test, p=0.684)

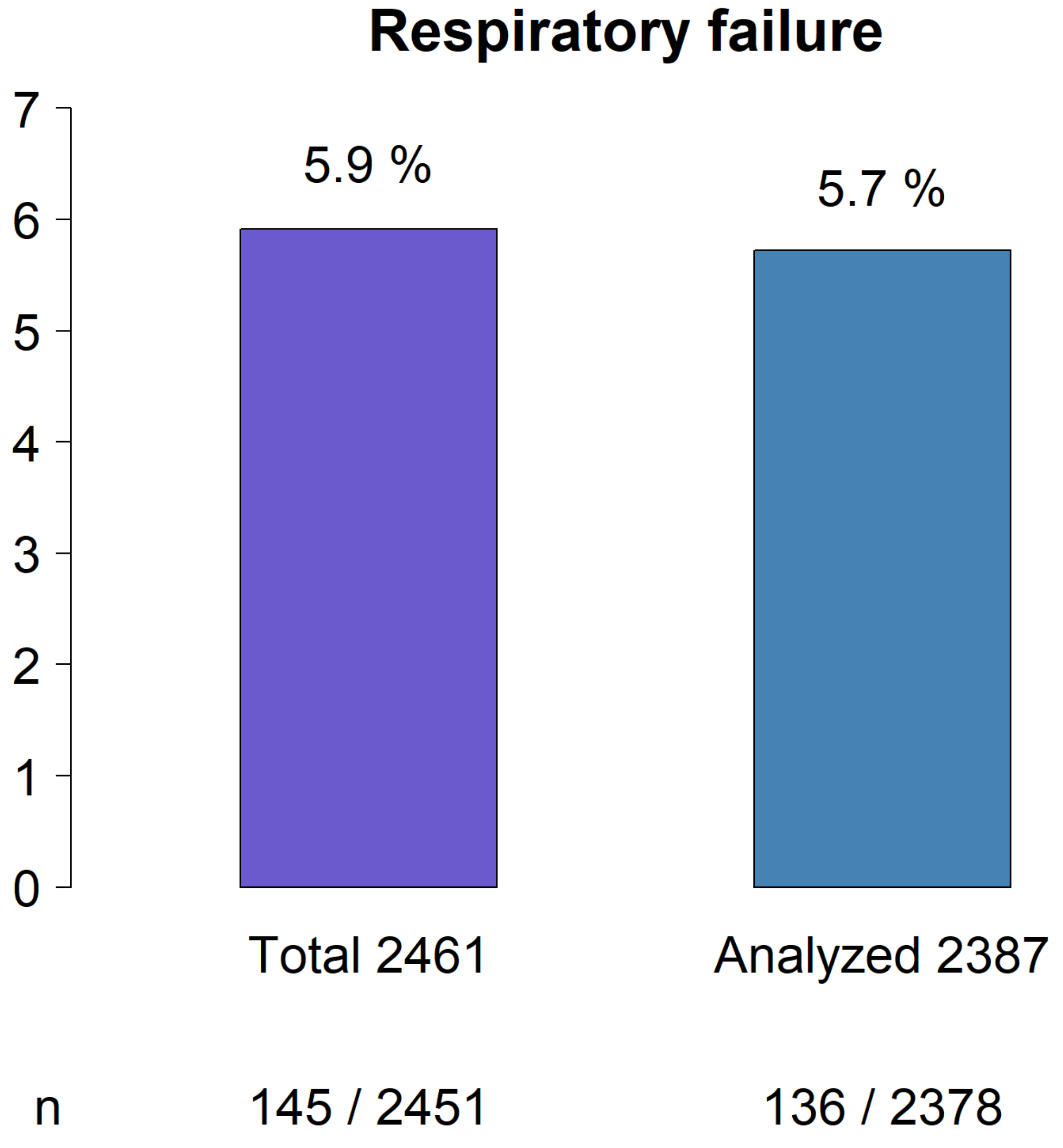

Supplementary Figure 13: Representativity analysis regarding heart failure showed no difference between the whole cohort and the analyzed population (Chi^2 test, p=0.653)

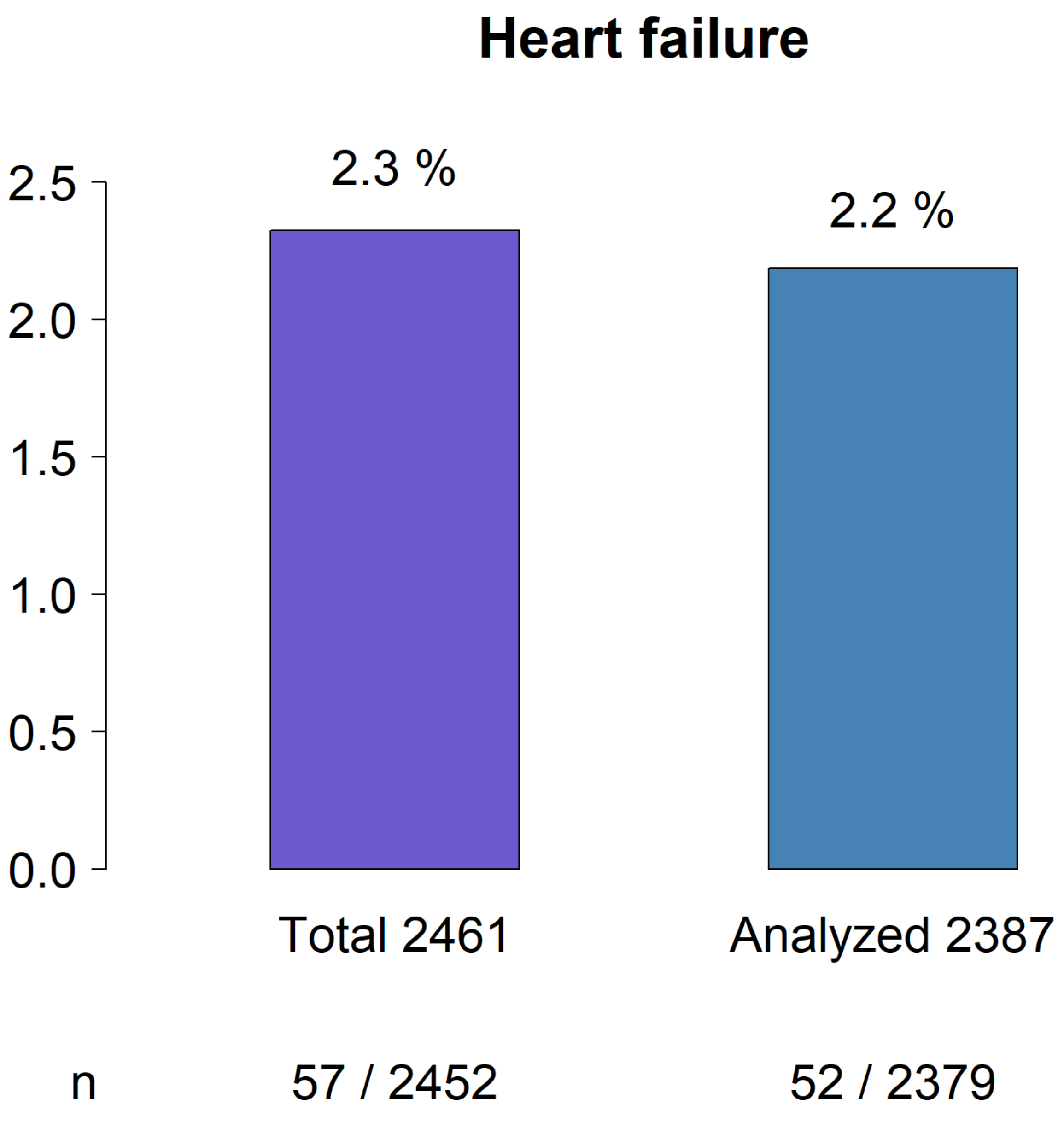

Supplementary Figure 14: Representativity analysis regarding renal failure showed no difference between the whole cohort and the analyzed population (Chi^2 test, p=0.638)

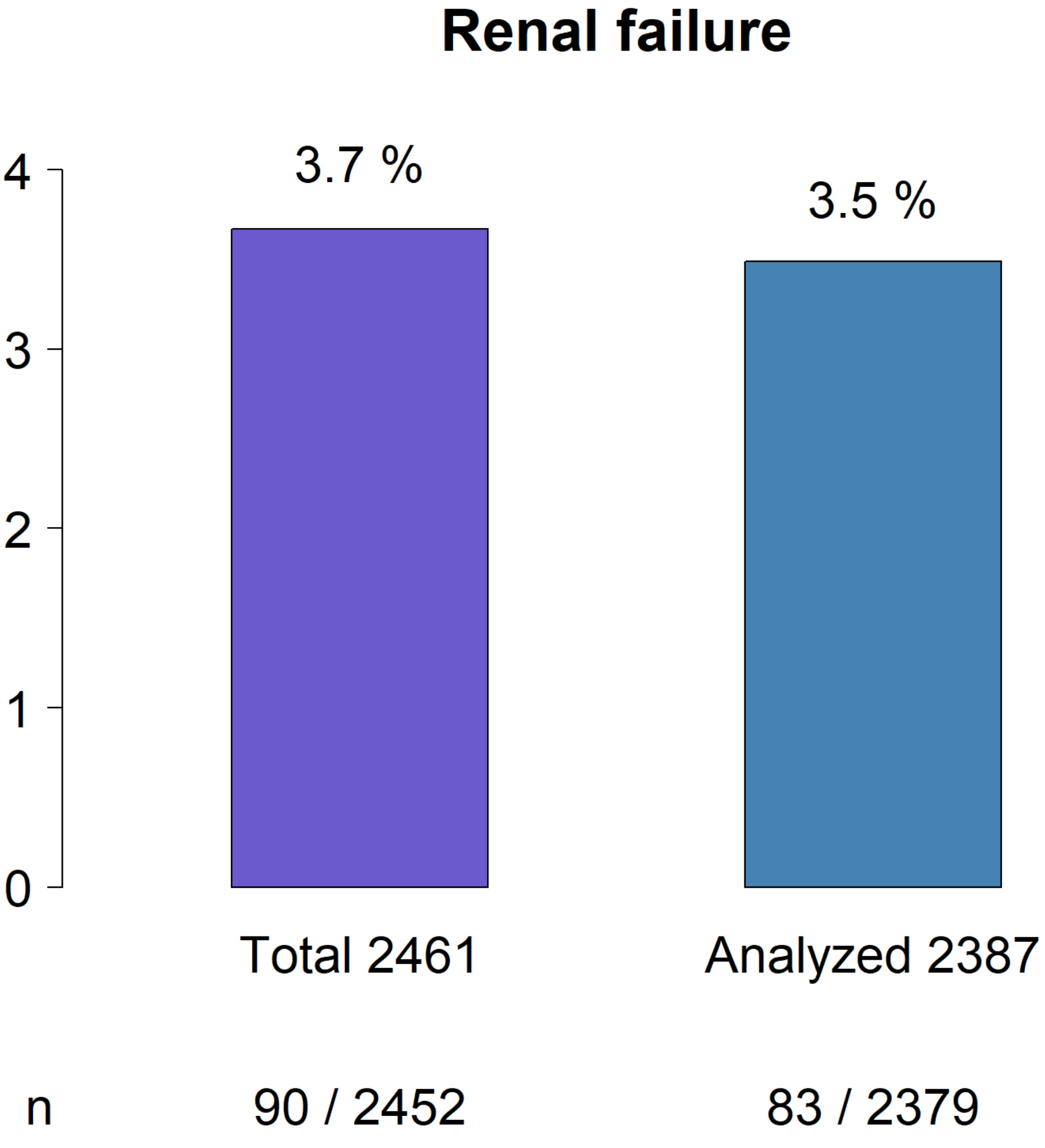

Supplementary Figure 15: Representativity analysis regarding etiology showed no difference between the whole cohort and the analyzed population (Chi^2 test, p=1.0)

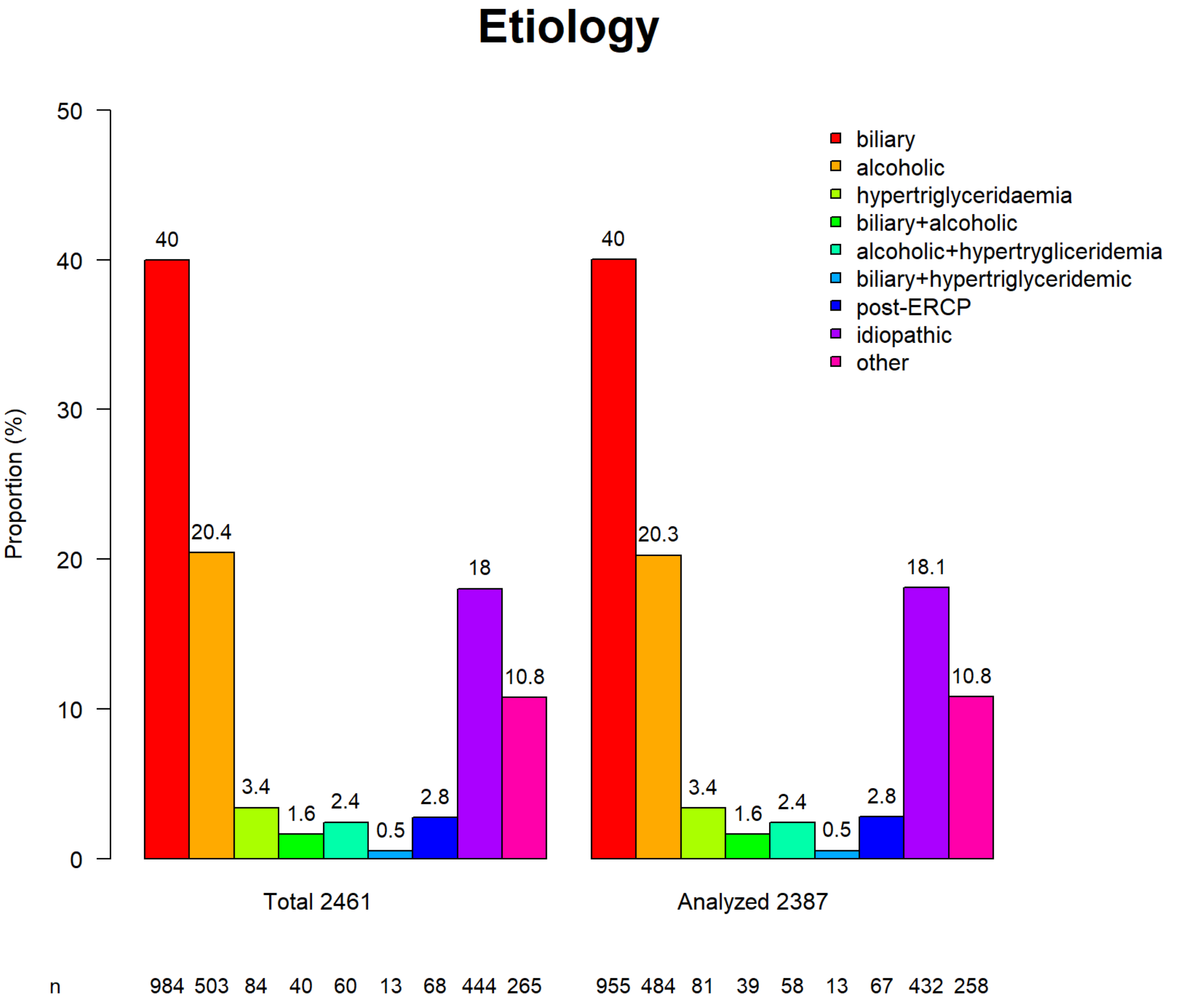

Supplement: Supplementary file 1 — Supplementary Information. [file 41598_2022_11517_MOESM1_ESM.pdf]
