# Supplementary material for: Early prediction of acute necrotizing pancreatitis by artificial intelligence: a prospective cohort-analysis of 2387 cases
Source: Sci Rep. 2022 May 12;12:7827. doi: 10.1038/s41598-022-11517-w (PMC9098474; doi:10.1038/s41598-022-11517-w)

Appendix B

Early prediction of acute necrotizing pancreatitis by artificial intelligence: A prospective cohort-analysis of 2387 cases

Szabolcs Kiss[1,2,3], József Pintér[4], Roland Molontay[4,5], Marcell Nagy[4], Nelli Farkas[2,6], Zoltán Sipos[2], Péter Fehérvári[2,7], László Pecze[2], Mária Földi[1,2,3], Áron Vincze[8], Tamás Takács[9], László Czakó[9], Ferenc Izbéki[10], Adrienn Halász[1,10], Eszter Boros[10], József Hamvas[11], Márta Varga[12], Artautas Mickevicius[13], Nándor Faluhelyi[14], Orsolya Farkas[14], Szilárd Váncsa[2,15], Rita Nagy[2,3,15], Stefania Bunduc[15,16], Péter Jenő Hegyi[15,17], Katalin Márta[15,17], Katalin Borka[15,18], Attila Doros[15,19], Nóra Hosszúfalusi[15,20], László Zubek[15,21], Bálint Erőss[15,17], Zsolt Molnár[15,21,22], Andrea Párniczky[2,3], Péter Hegyi[2,15,17 #], Andrea Szentesi[1,2,15 #, *], Hungarian Pancreatic Study Group[17,§]

[1]Doctoral School of Clinical Medicine, Faculty of Medicine, University of Szeged, Szeged,
[2]Institute for Translational Medicine, Szentágothai Research Centre, Medical School, University of Pécs, Pécs, Hungary,
[3]Heim Pál National Pediatric Institute, Budapest, Hungary
[4]Human and Social Data Science Lab, Budapest University of Technology and Economics, Budapest, Hungary,
[5]Stochastics Research Group, Hungarian Academy of Sciences - Budapest University of Technology and Economics, Budapest, Hungary,
[6]Institute of Bioanalysis, Medical School, University of Pécs, Pécs, Hungary,
[7]Department of Biomathematics and Informatics, University of Veterinary Medicine, Budapest, Hungary
[8]Division of Gastroenterology, First Department of Medicine, Medical School, University of Pécs, Pécs, Hungary,
[9]Department of Medicine, University of Szeged, Szeged, Hungary,
[10]Department of Internal Medicine, Szent György Teaching Hospital of County Fejér, Székesfehérvár, Hungary,
[11]Bajcsy-Zsilinszky Hospital, Budapest, Hungary,
[12]Department of Gastroenterology, BMKK dr Rethy Pal Hospital, Békéscsaba, Hungary,
[13]Vilnius University Hospital Santaros Clinics, Clinics of Abdominal Surgery, Nephrourology and Gastroenterology, Faculty of Medicine, Vilnius University, Vilnius, Lithuania
[14]Department of Medical Imaging, Medical School, University of Pécs, Pécs, Hungary
[15]Centre for Translational Medicine, Semmelweis University, Budapest, Hungary
[16]Doctoral school, Carol Davila University of Medicine and Pharmacy, Bucharest, Romania
Address: RO-050474 Bulevardul Eroii Sanitari 8., București, Romania
[17]Division of Pancreatic Diseases, Heart and Vascular Center, Semmelweis University, Budapest, Hungary
[18]2nd Department of Pathology, Semmelweis University, Budapest, Hungary
[19]Department of Transplantation and Surgery, Semmelweis University, Budapest, Hungary
[20]Department of Internal Medicine and Hematology, Semmelweis University, Budapest, Hungary
Address: H-1088 Budapest, Szentkirályi u. 46., Hungary
[21]Department of Anaesthesiology and Intensive Therapy, Semmelweis University, Budapest, Hungary
[22]Department of Anaesthesiology and Intensive Therapy, Poznan University of Medical Sciences, Poznan, Poland

# equally contributed

*Correspondence: Andrea Szentesi, Address: Institute for Translational Medicine, Medical School, University of Pécs; 12 Ifjúság u., Pécs, 7624 Hungary; Mobile: +36 (30) 342 1481; E-mail: szentesiai@gmail.com.

§A list of authors and their affiliations appears at the end of the paper. A full list of members and contributors of the Hungarian Pancreatic Study Group can be found in Appendix A.

Supplementary Figure 16: The comparison in terms of age showed statistically significant difference between acute pancreatitis patients with and without necrosis development (Kolmogorov–Smirnov test, p<0.001).

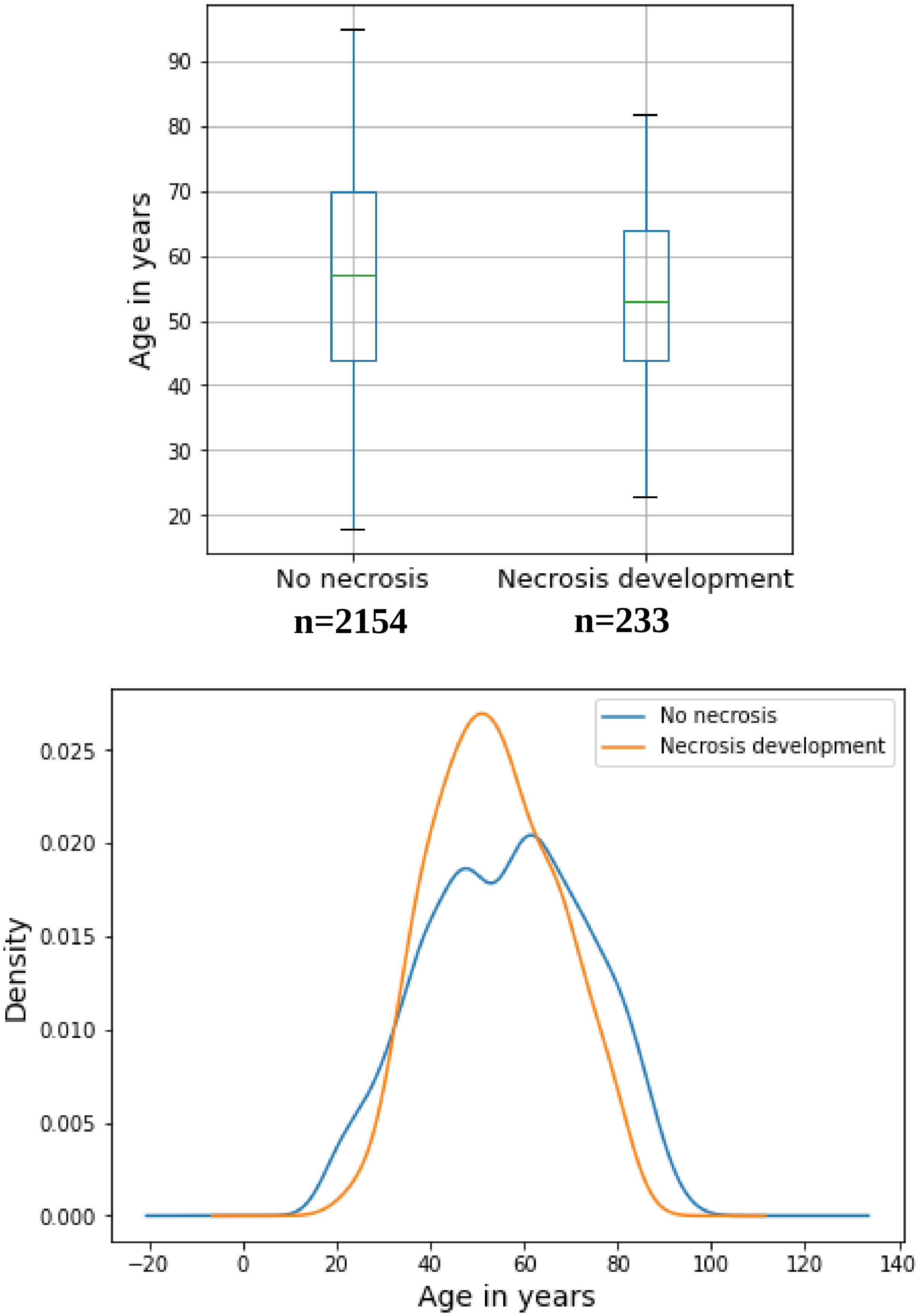

Supplementary Figure 17: The comparison in terms of gender distribution showed statistically significant difference between acute pancreatitis patients with and without necrosis development (Chi2 test, p=0.018).

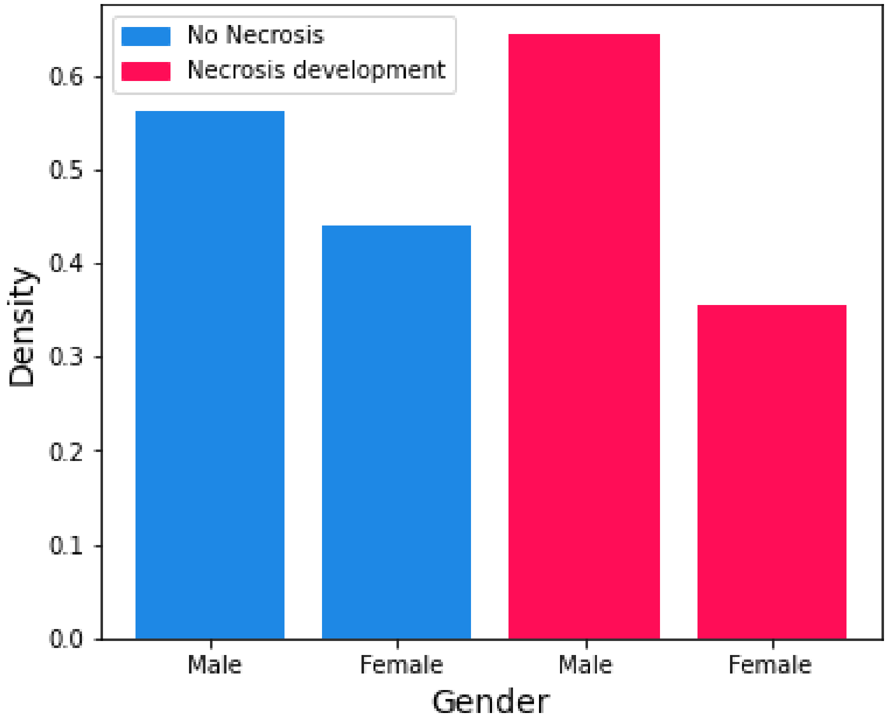

n=2154 **n=233**

Supplementary Figure 18: The comparison in terms of body mass index showed statistically significant difference between acute pancreatitis patients with and without necrosis development (Kolmogorov–Smirnov test, p<0.001).

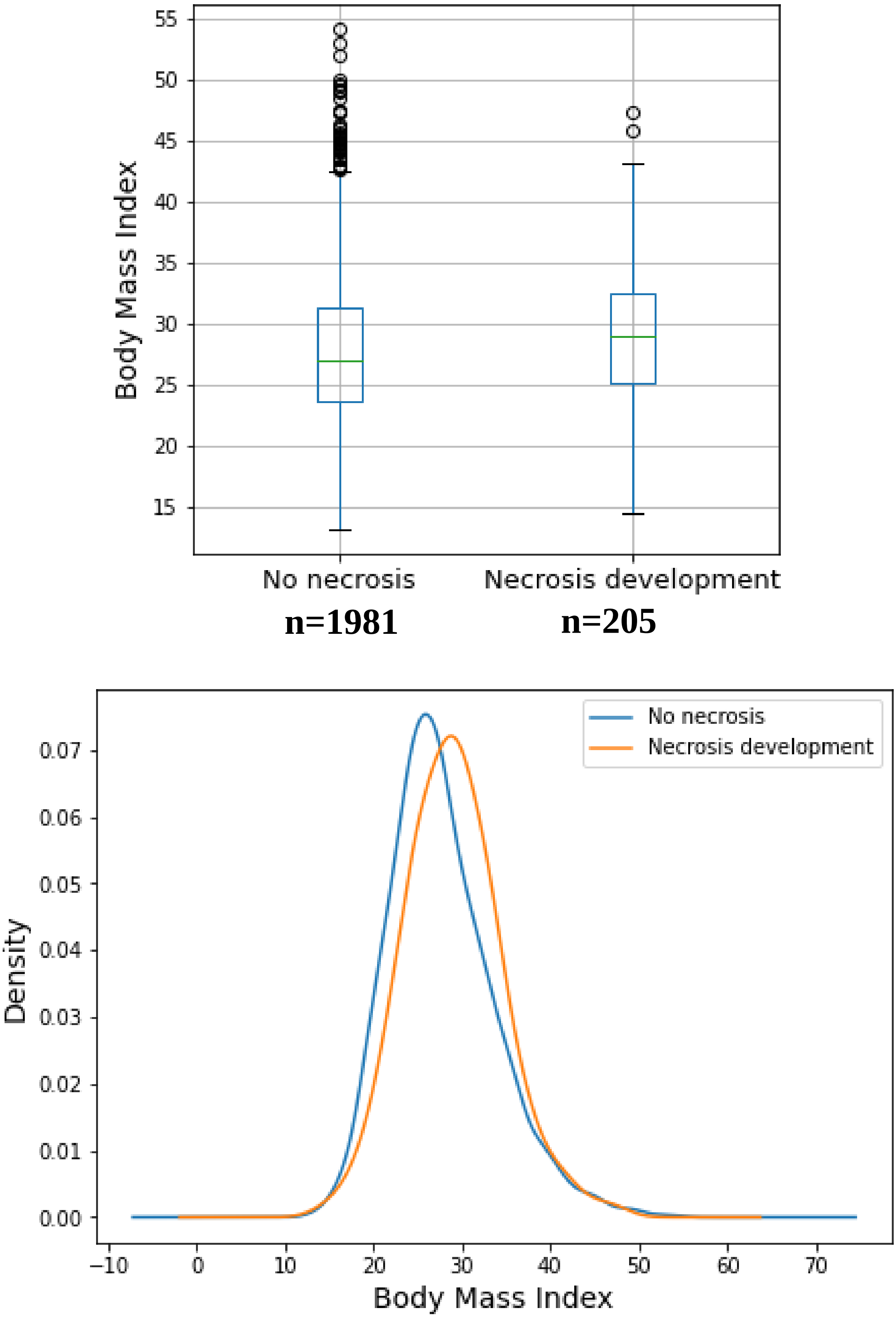

Supplementary Figure 19: The comparison in terms of amylase did not show statistically significant difference between acute pancreatitis patients with and without necrosis development (Kolmogorov–Smirnov test, p=0.053).

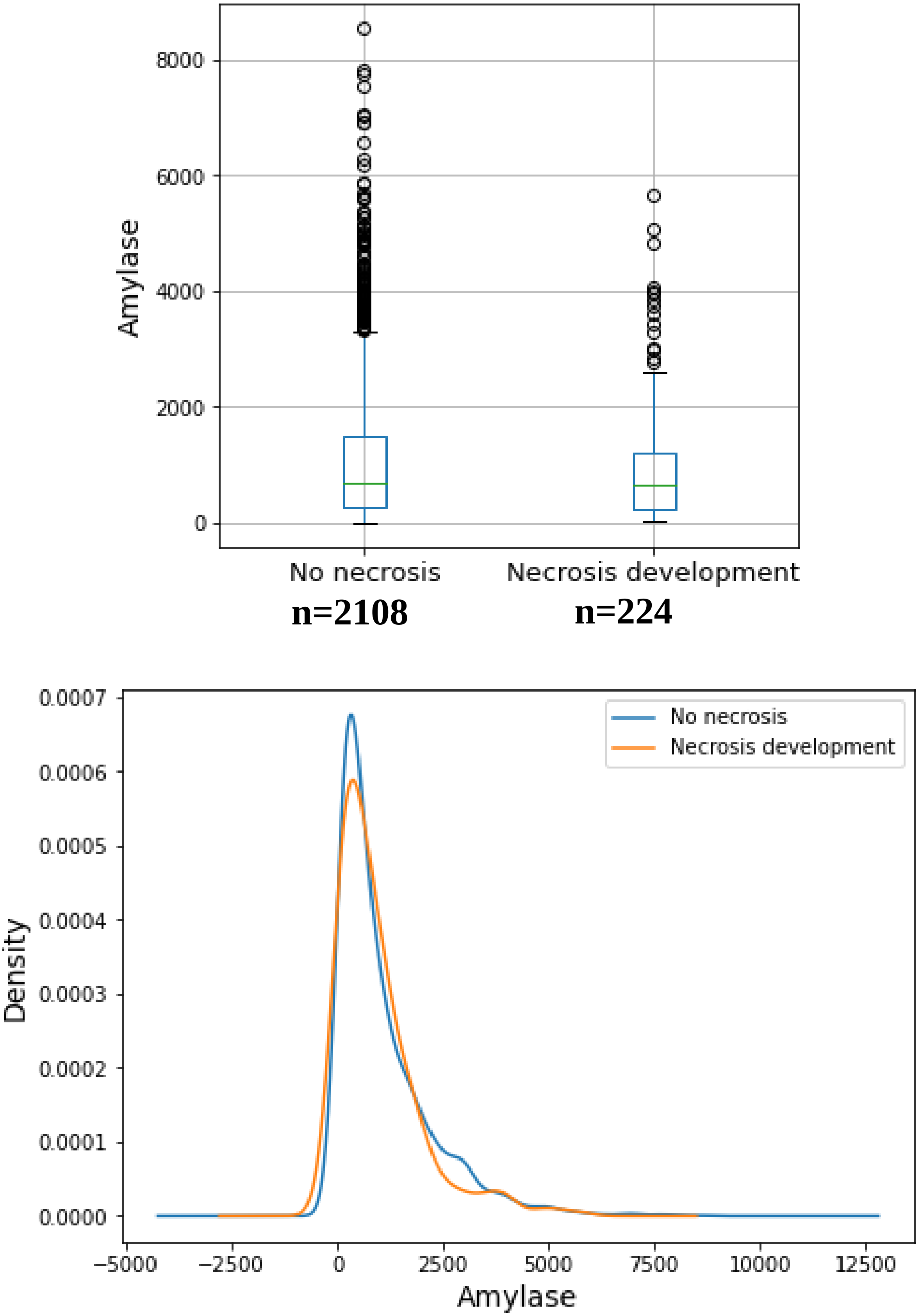

Supplementary Figure 20: The comparison in terms of lipase did not show statistically significant difference between acute pancreatitis patients with and without necrosis development (Kolmogorov–Smirnov test, p=0.380).

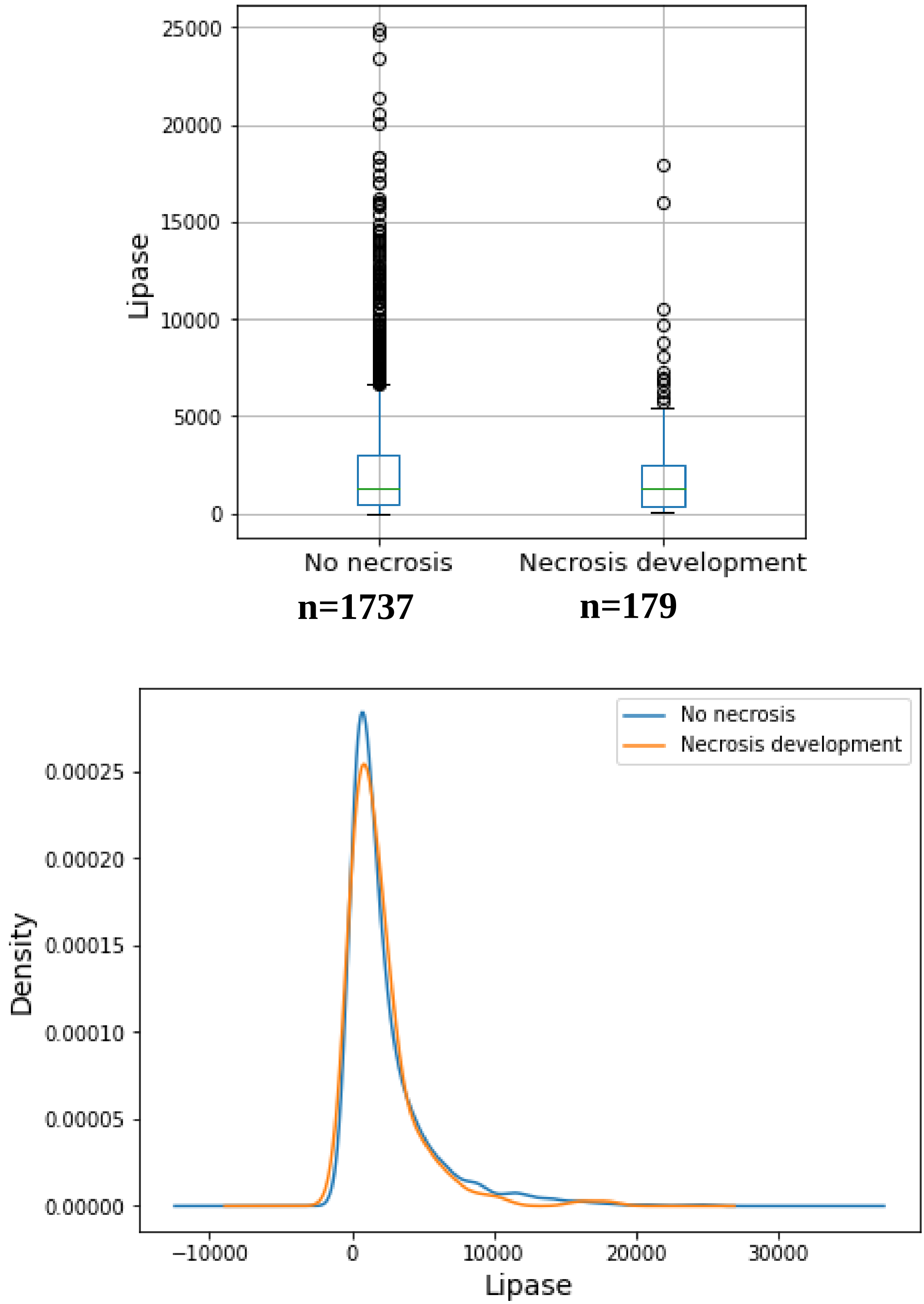

Supplementary Figure 21: The comparison in terms of total white blood cell count showed statistically significant difference between acute pancreatitis patients with and without necrosis development (Kolmogorov–Smirnov test, p<0.001).

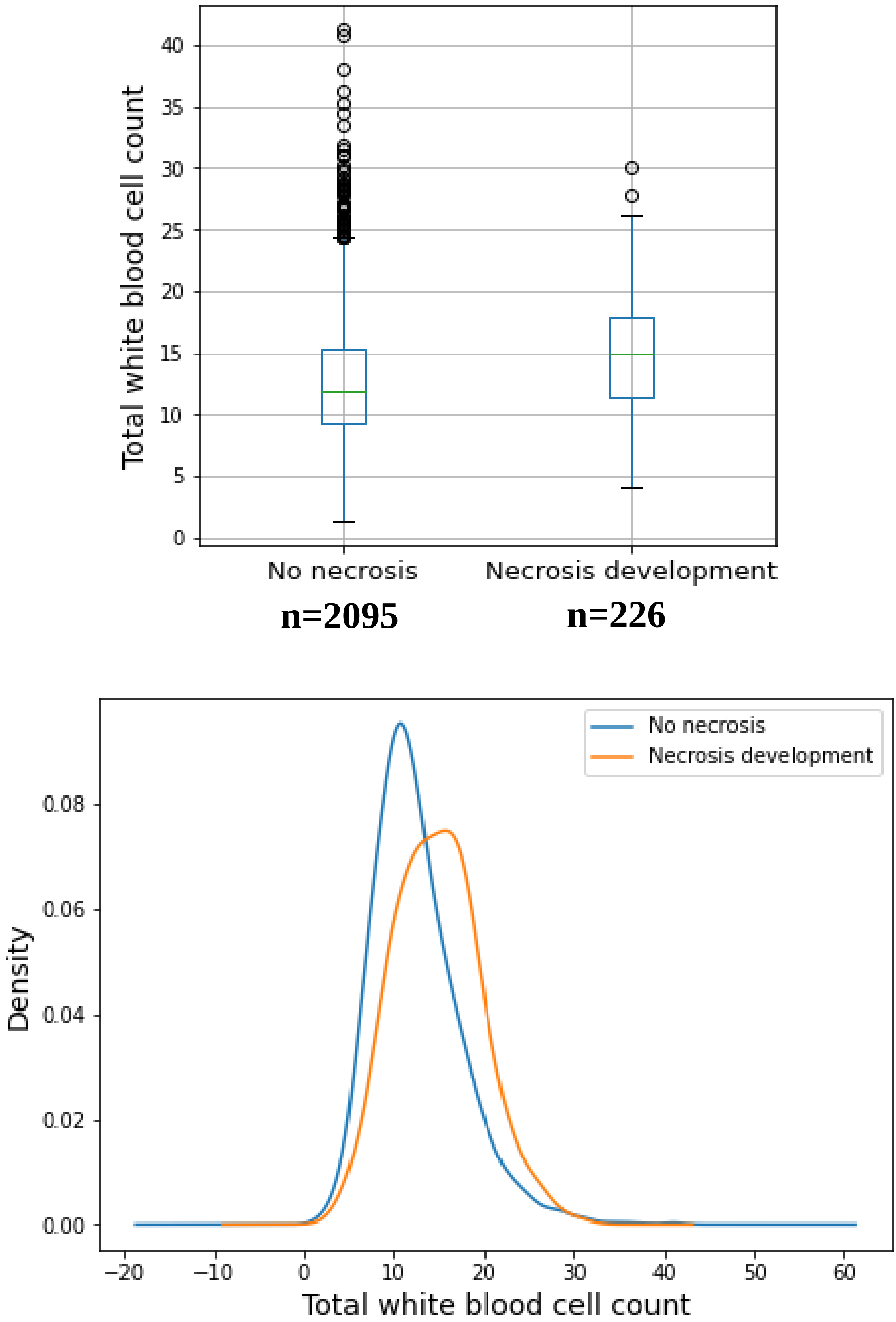

Supplementary Figure 22: The comparison in terms of red blood cell count showed statistically significant difference between acute pancreatitis patients with and without necrosis development (Kolmogorov–Smirnov test, p<0.001).

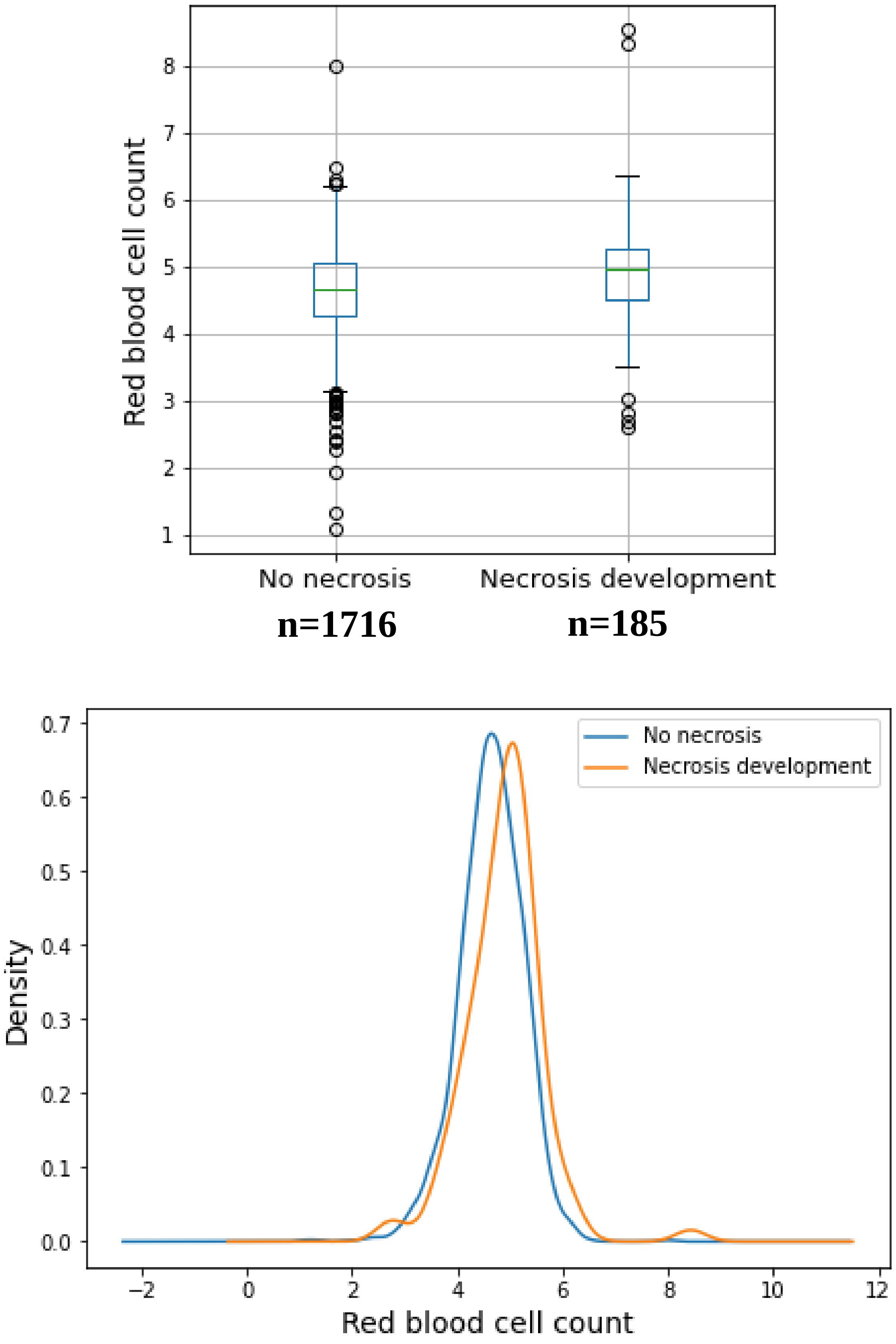

Supplementary Figure 23: The comparison in terms of hemoglobin showed statistically significant difference between acute pancreatitis patients with and without necrosis development (Kolmogorov–Smirnov test, p<0.001).

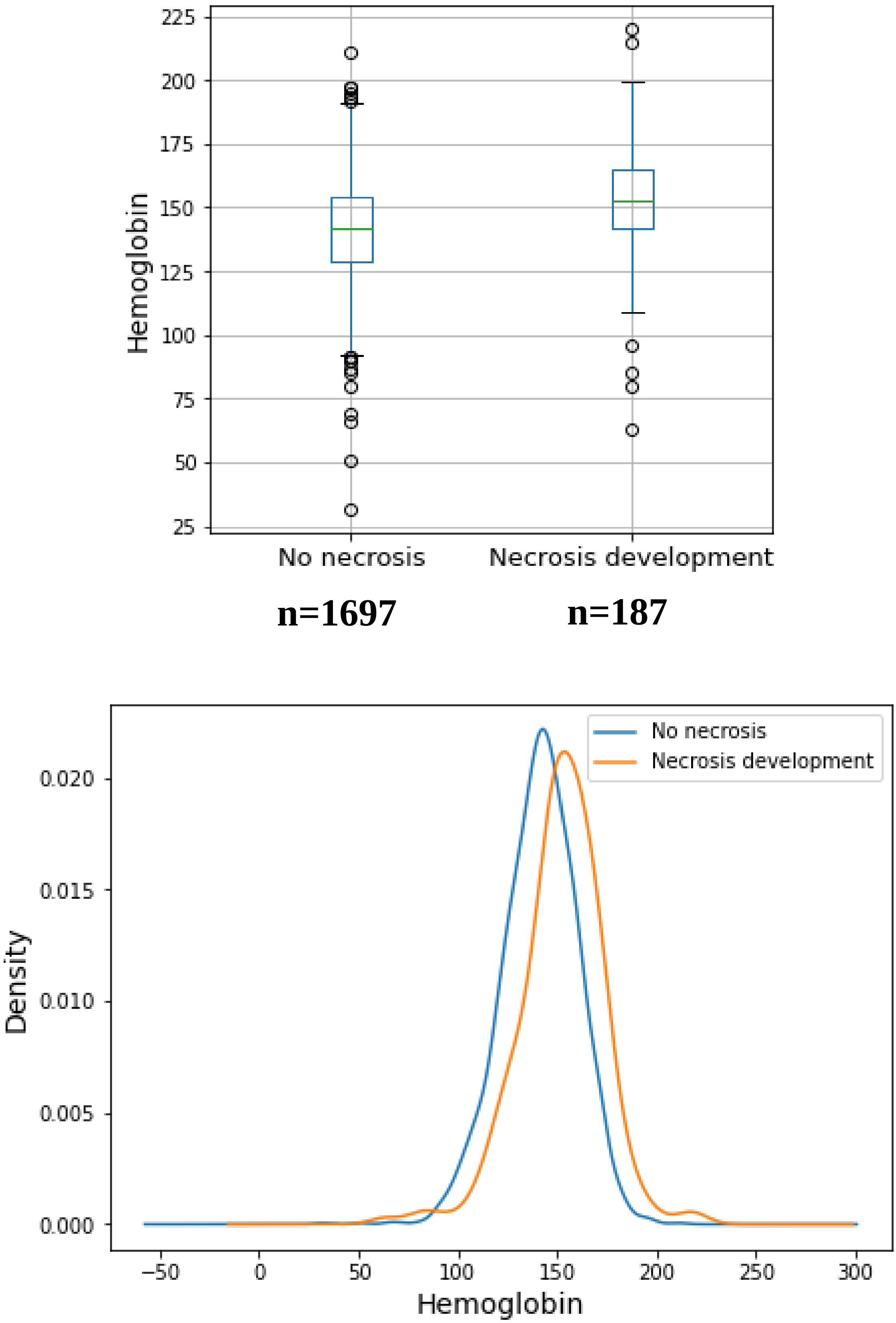

Supplementary Figure 24: The comparison in terms of hematocrit showed statistically significant difference between acute pancreatitis patients with and without necrosis development (Kolmogorov–Smirnov test, p<0.001).

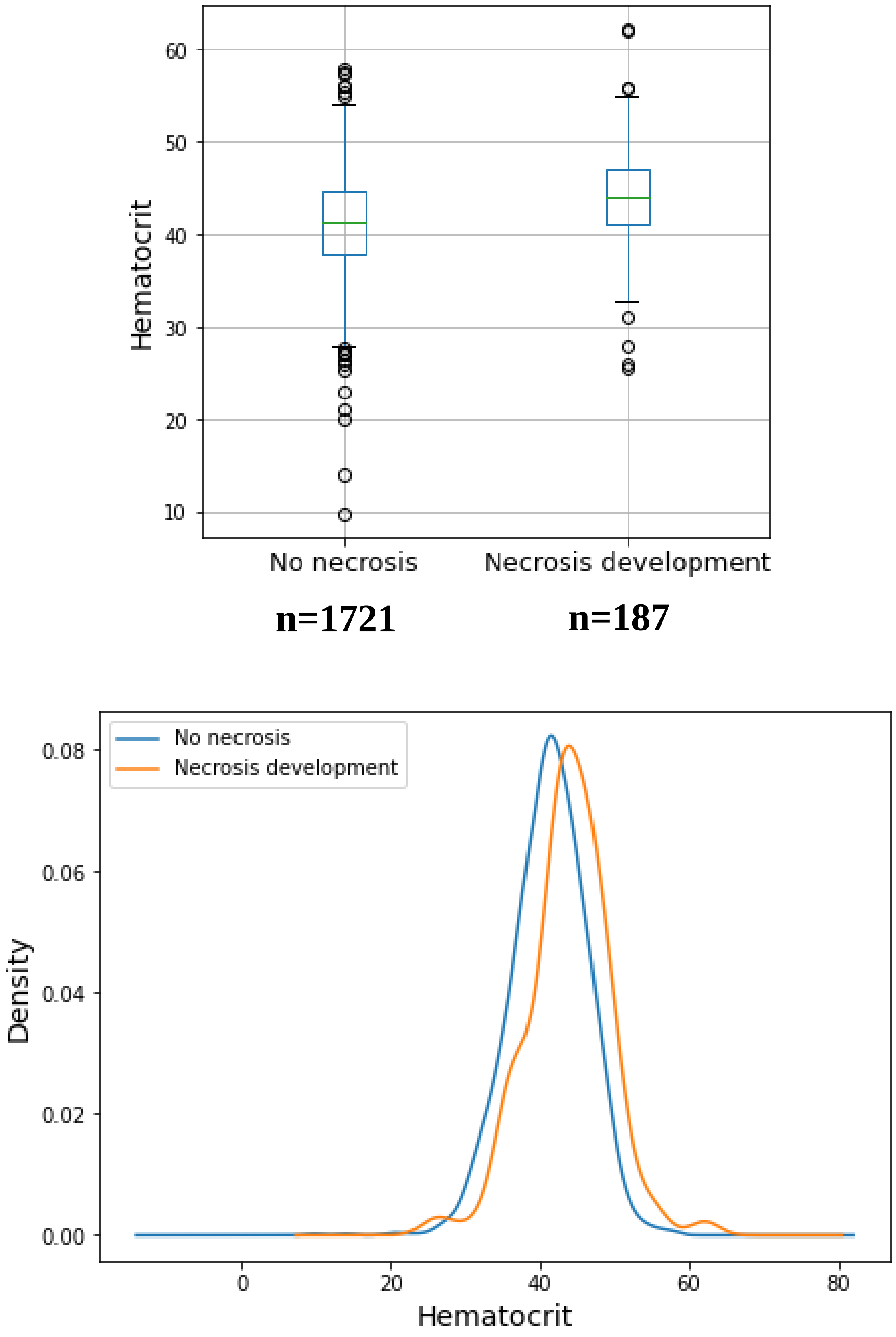

Supplementary Figure 25: The comparison in terms of thrombocyte count did not show statistically significant difference between acute pancreatitis patients with and without necrosis development (Kolmogorov–Smirnov test, p=0.524).

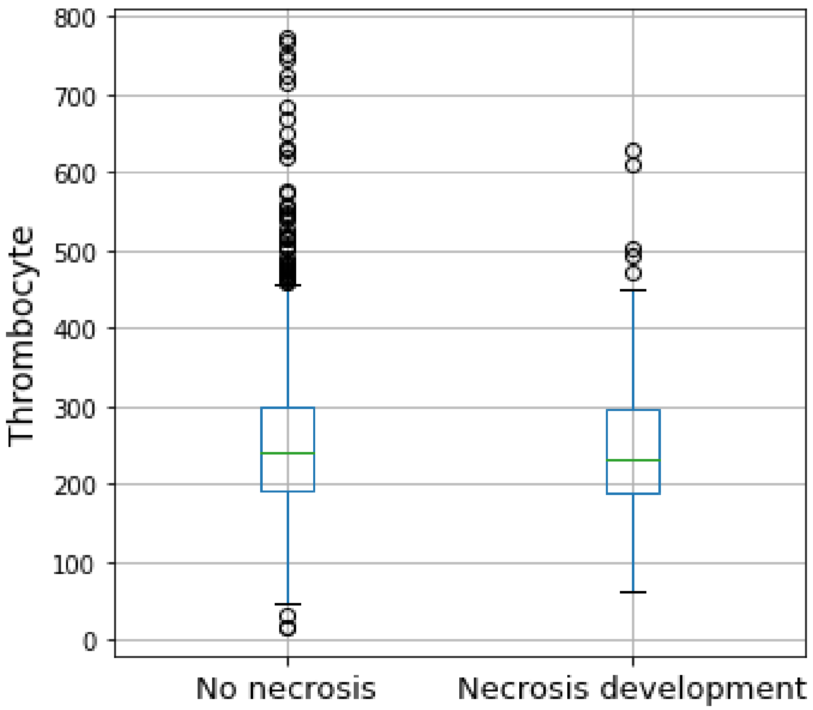

n=1715 **n=187**

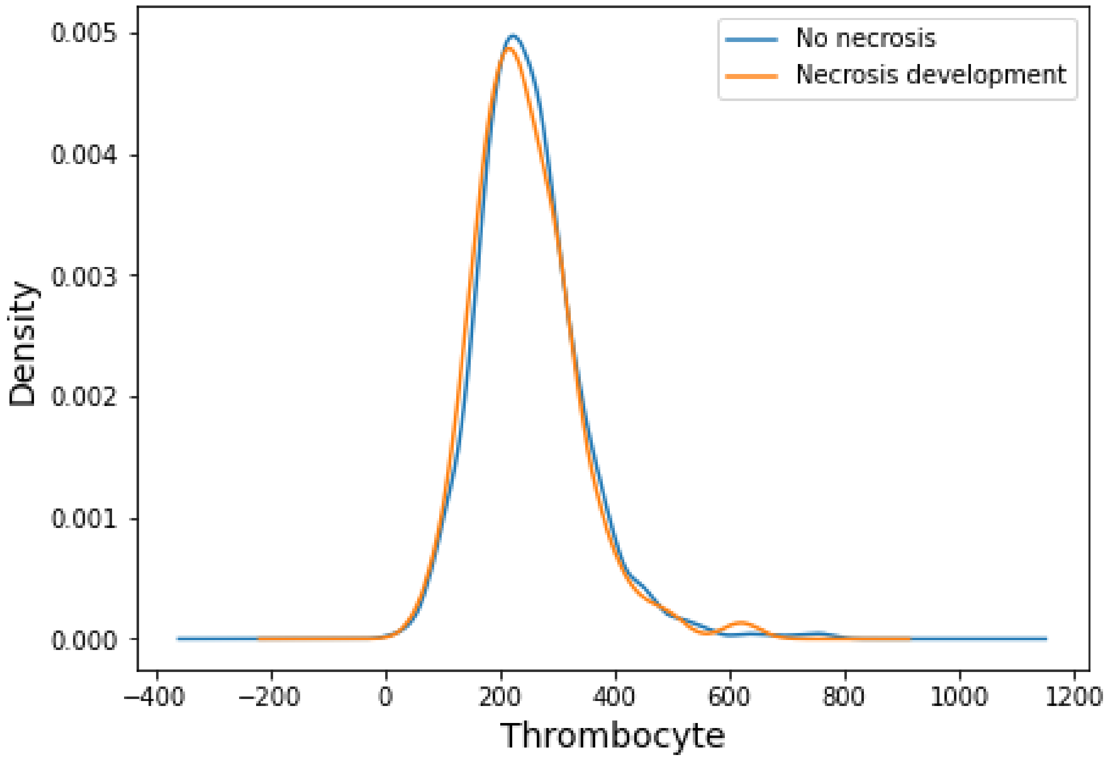

Supplementary Figure 26: The comparison in terms of glucose showed statistically significant difference between acute pancreatitis patients with and without necrosis development (Kolmogorov–Smirnov test, p<0.001).

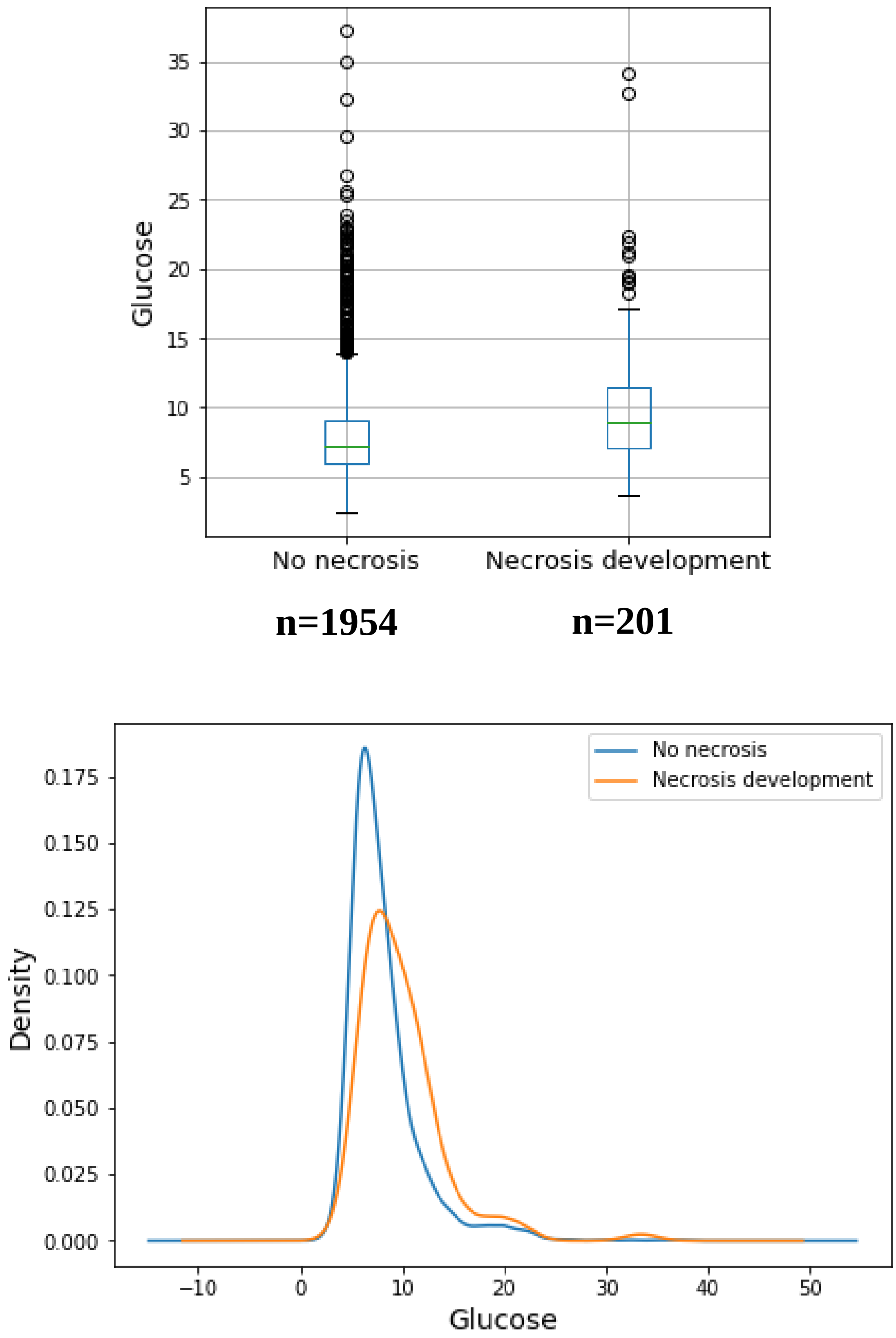

Supplementary Figure 27: The comparison in terms of glycated hemoglobin did not show statistically significant difference between acute pancreatitis patients with and without necrosis development (Kolmogorov–Smirnov test, p=0.567).

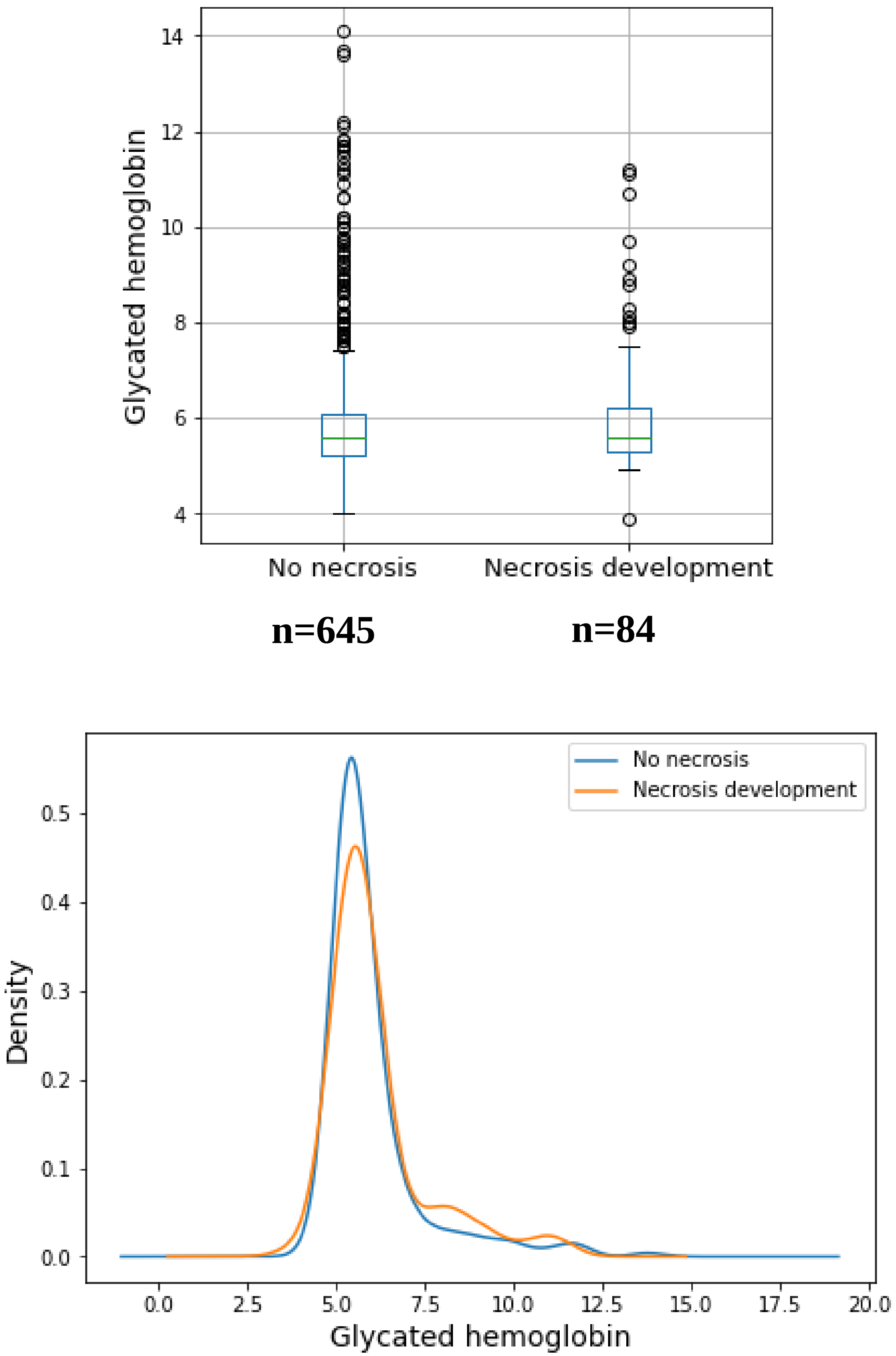

Supplementary Figure 28: The comparison in terms of blood urea nitrogen did not show statistically significant difference between acute pancreatitis patients with and without necrosis development (Kolmogorov–Smirnov test, p=0.168).

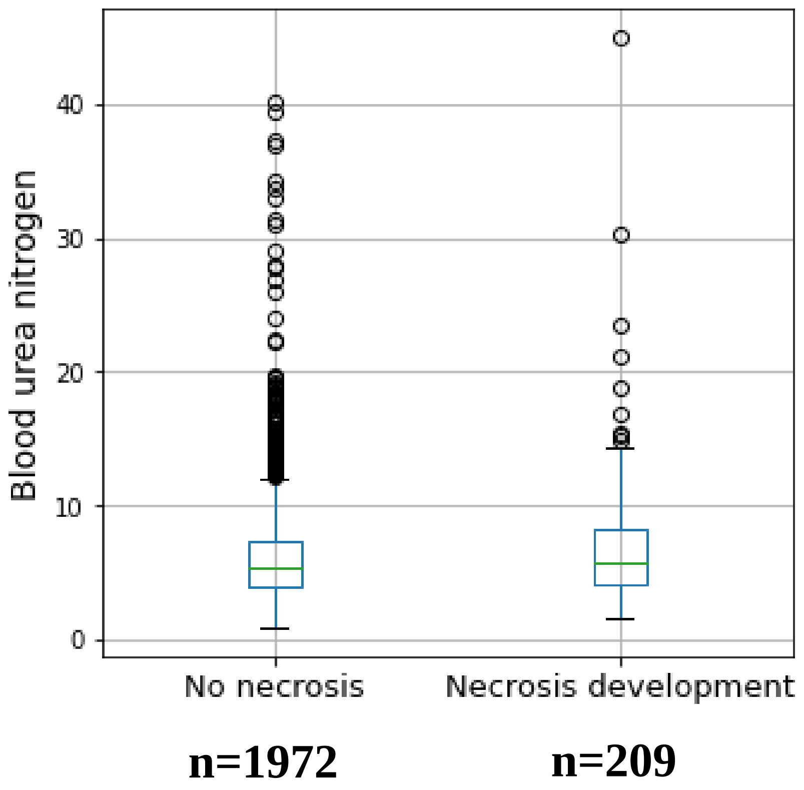

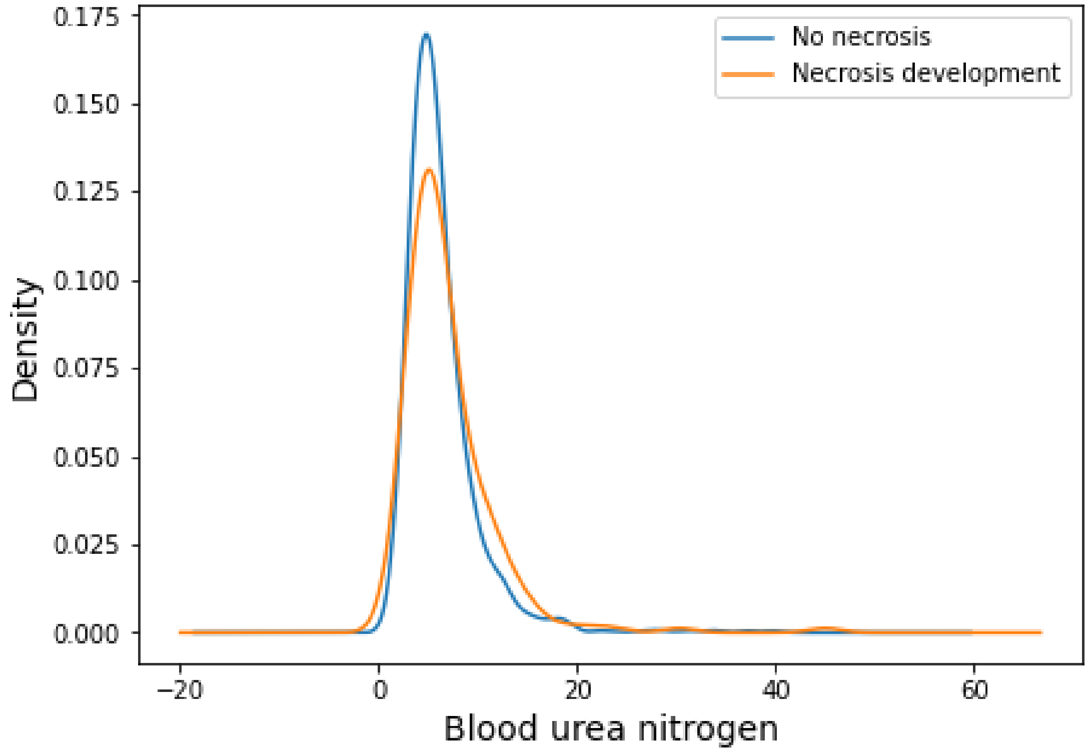

Supplementary Figure 29: The comparison in terms of creatinin did not show statistically significant difference between acute pancreatitis patients with and without necrosis development (Kolmogorov–Smirnov test, p=0.292).

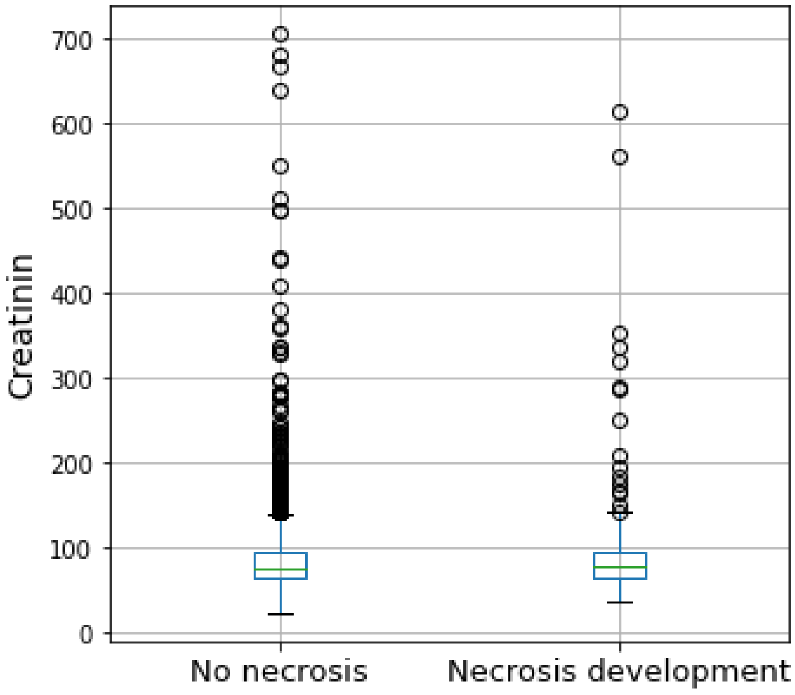

n=2019 **n=215**

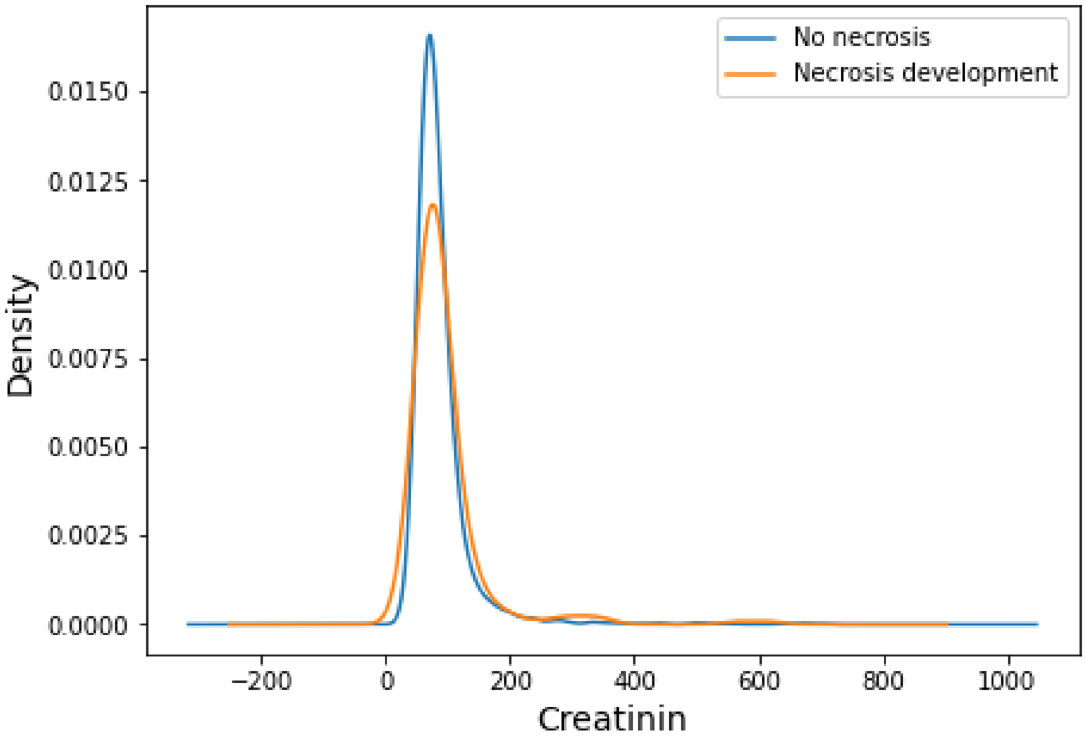

Supplementary Figure 30: The comparison in terms of glomerular filtration rate did not show statistically significant difference between acute pancreatitis patients with and without necrosis development (Kolmogorov–Smirnov test, p=0.598).

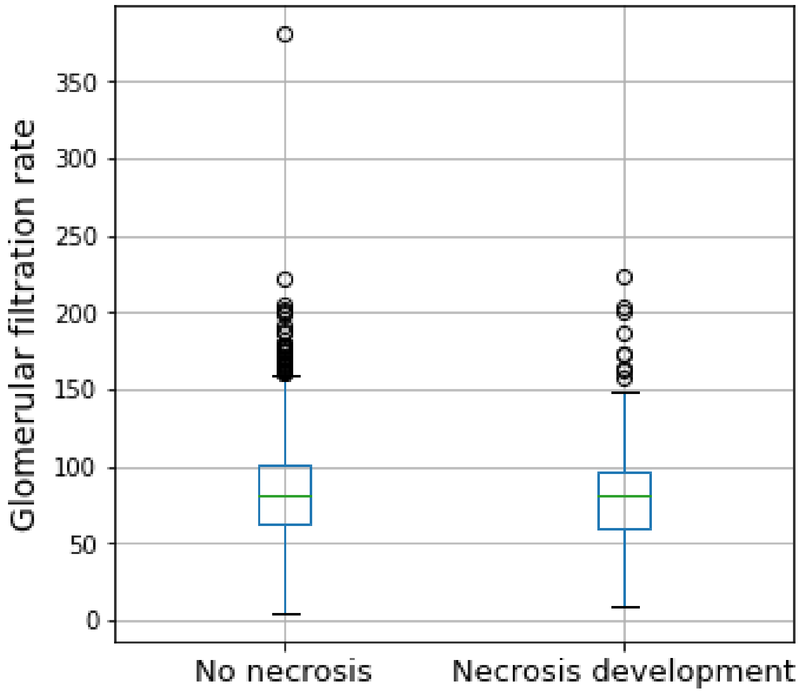

n=1982 **n=208**

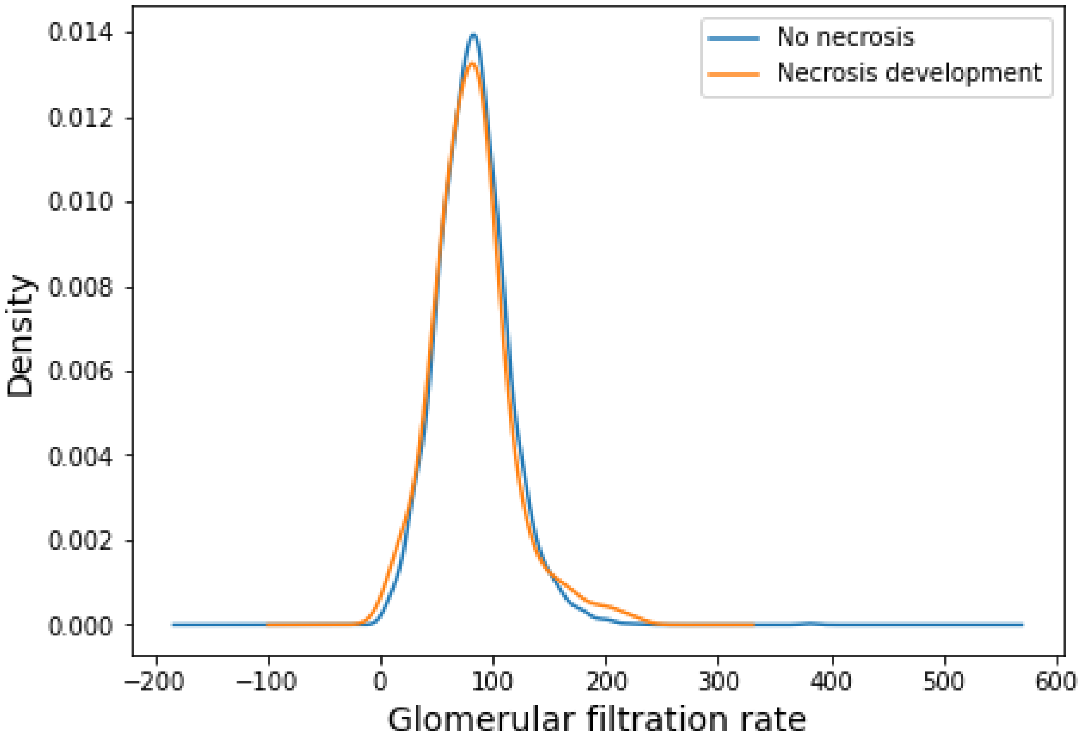

Supplementary Figure 31: The comparison in terms of C-reactive protein showed statistically significant difference between acute pancreatitis patients with and without necrosis development (Kolmogorov–Smirnov test, p<0.001).

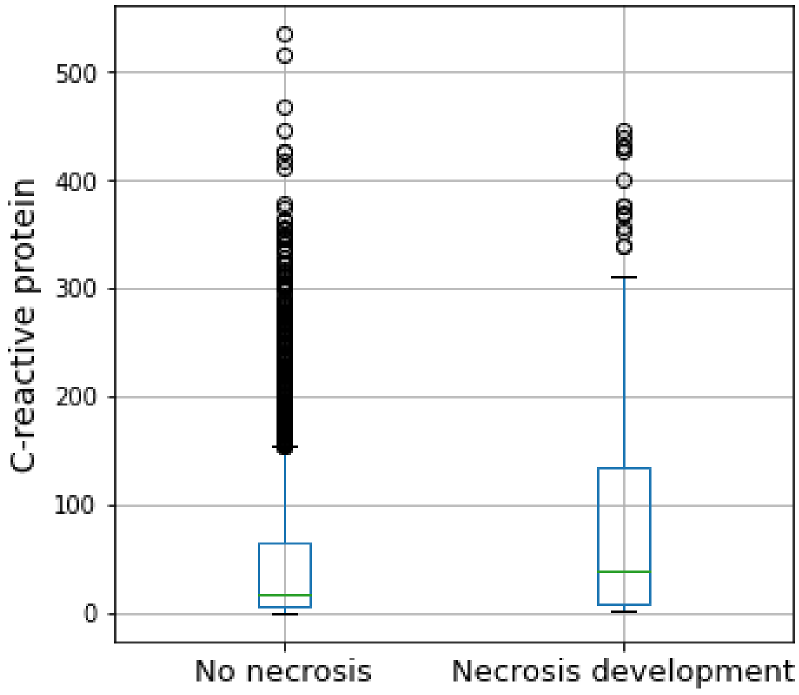

n=2014 **n=209**

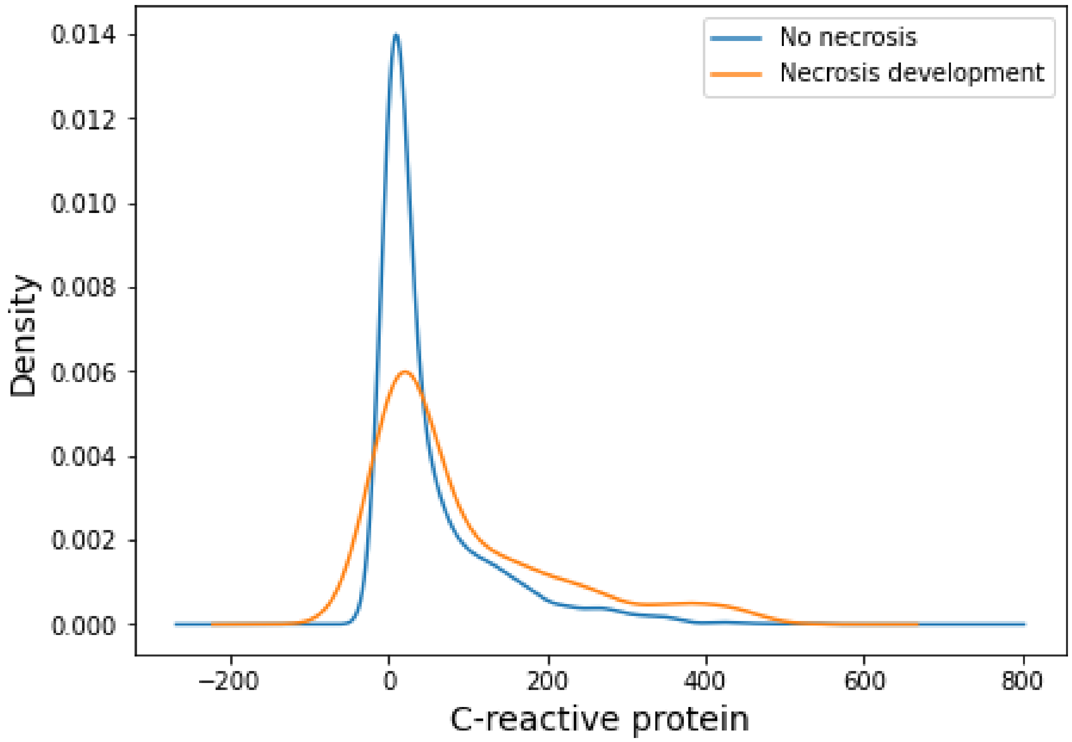

Supplementary Figure 32: The comparison in terms of procalcitonin showed statistically significant difference between acute pancreatitis patients with and without necrosis development (Kolmogorov–Smirnov test, p=0.047).

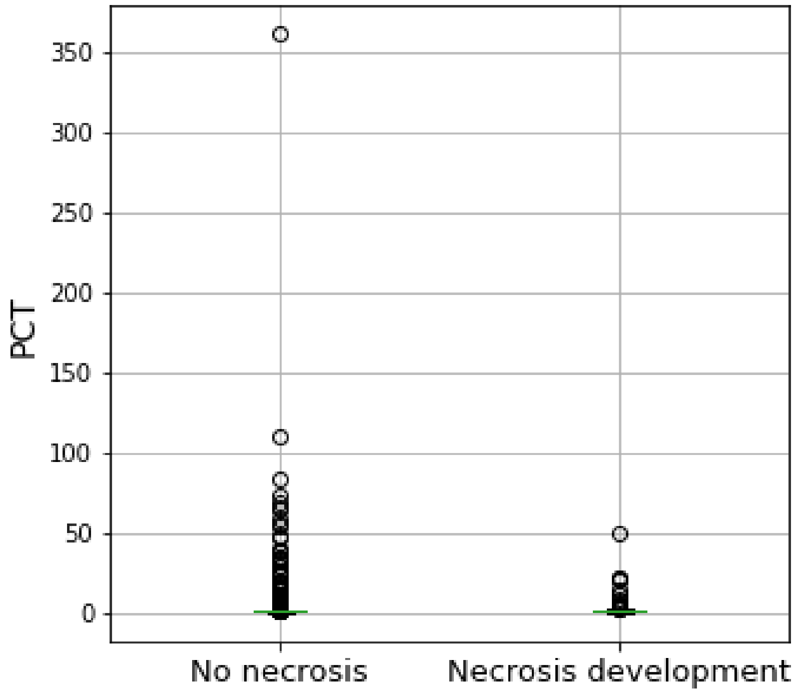

n=776 **n=113**

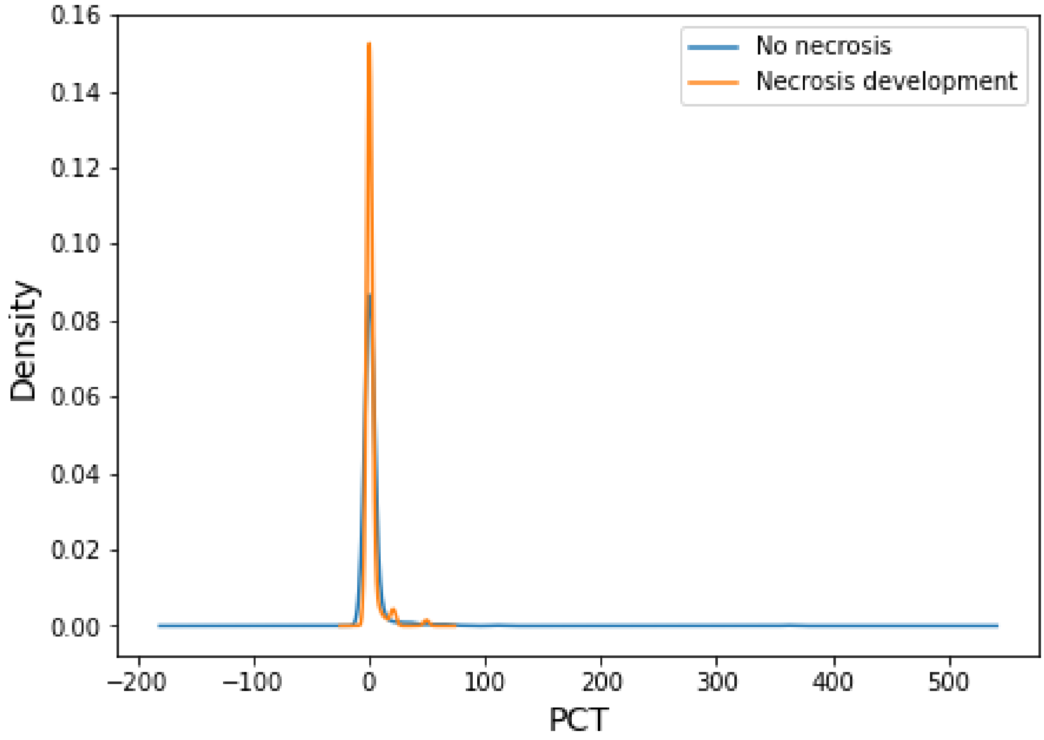

Supplementary Figure 33: The comparison in terms of lactate dehydrogenase showed statistically significant difference between acute pancreatitis patients with and without necrosis development (Kolmogorov–Smirnov test, p=0.002).

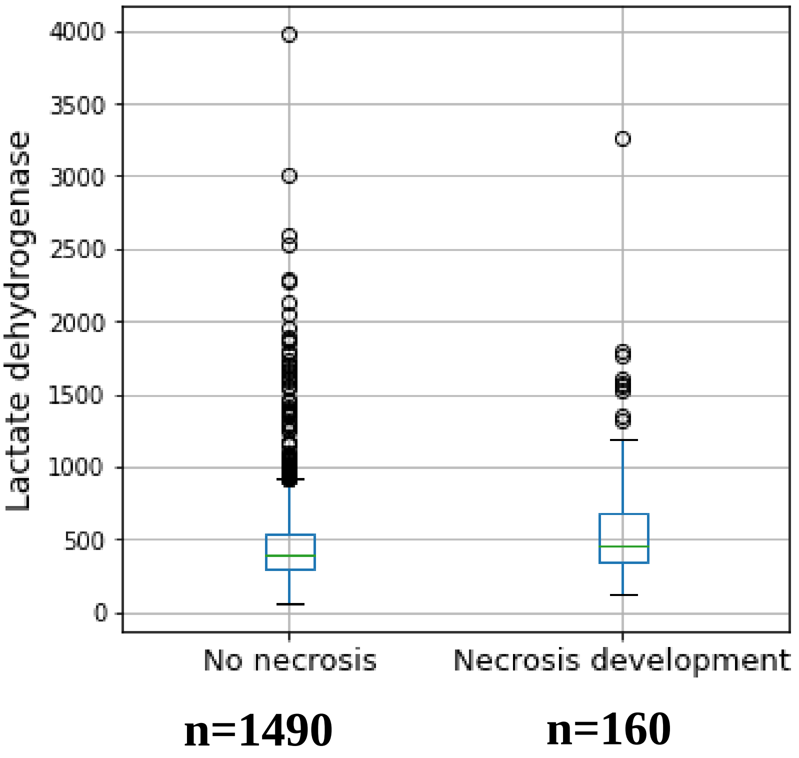

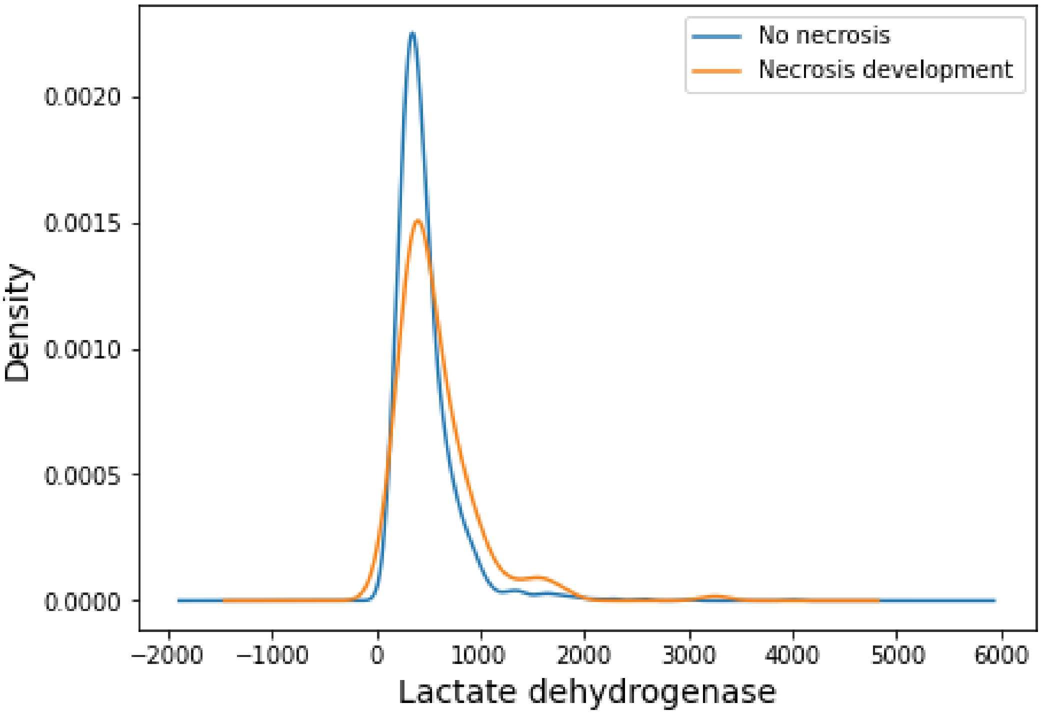

Supplementary Figure 34: The comparison in terms of calcium showed statistically significant difference between acute pancreatitis patients with and without necrosis development (Kolmogorov–Smirnov test, p<0.001).

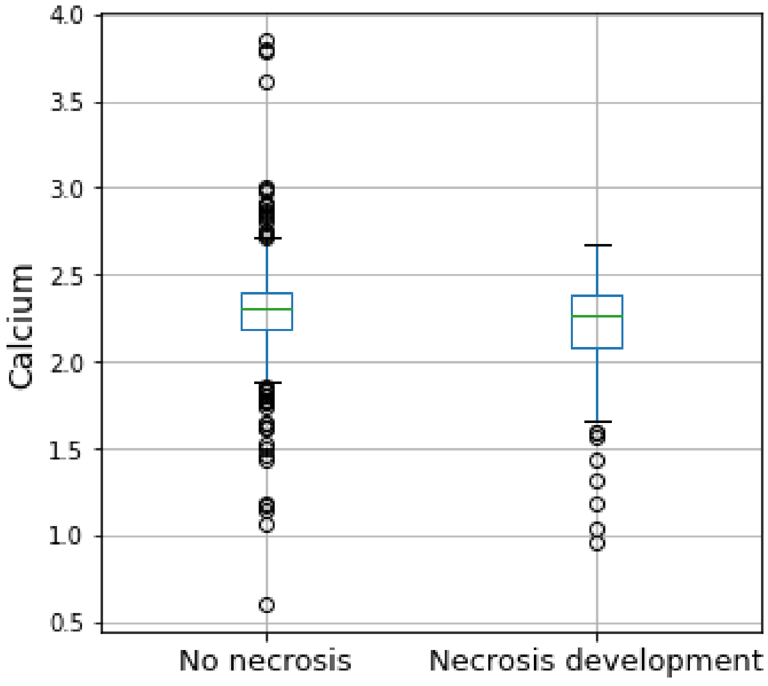

n=1392 **n=160**

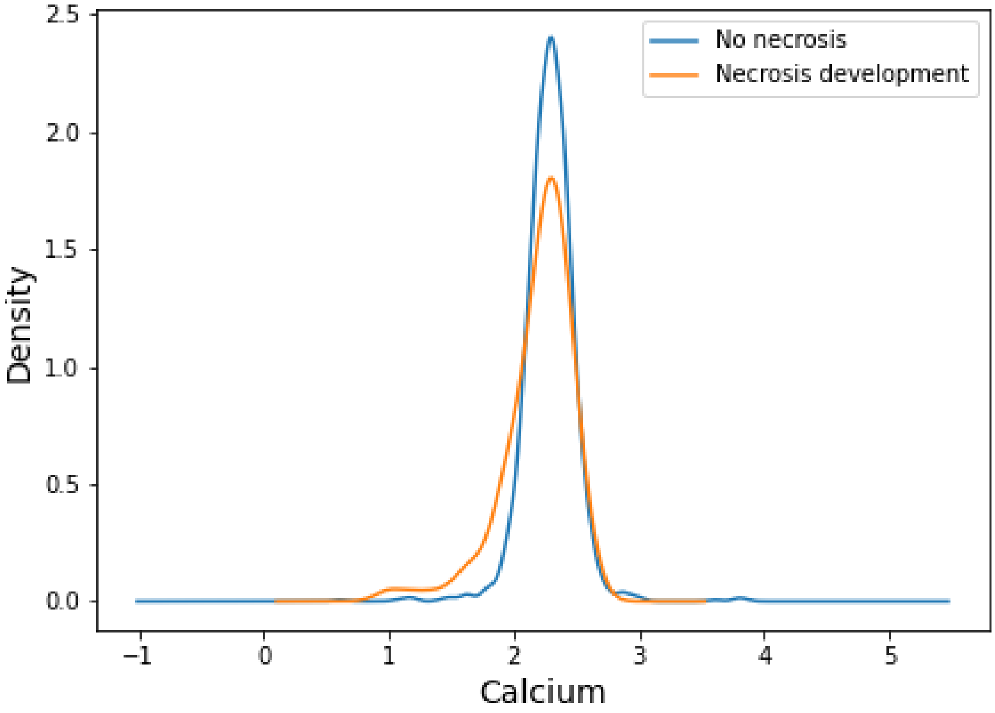

Supplementary Figure 35: The comparison in terms of sodium did not show statistically significant difference between acute pancreatitis patients with and without necrosis development (Kolmogorov–Smirnov test, p=0.567).

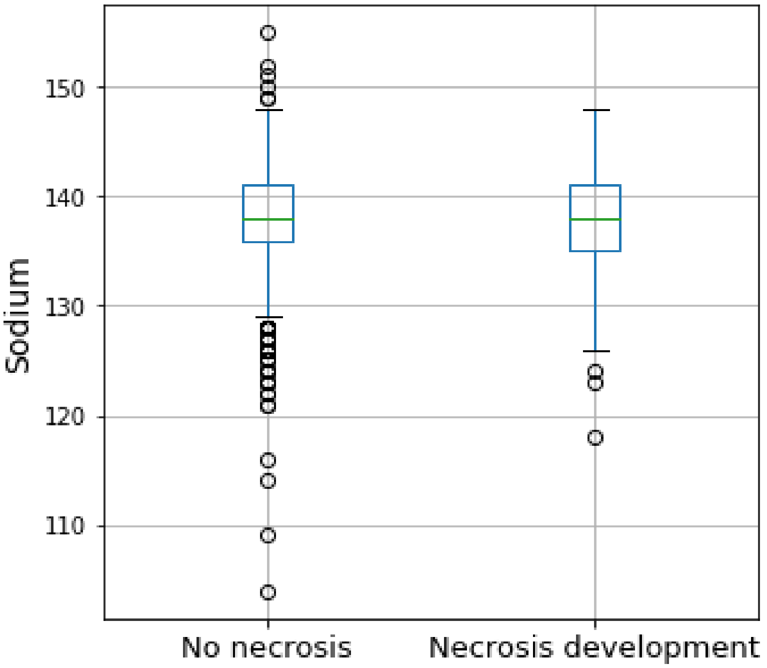

n=1643 **n=174**

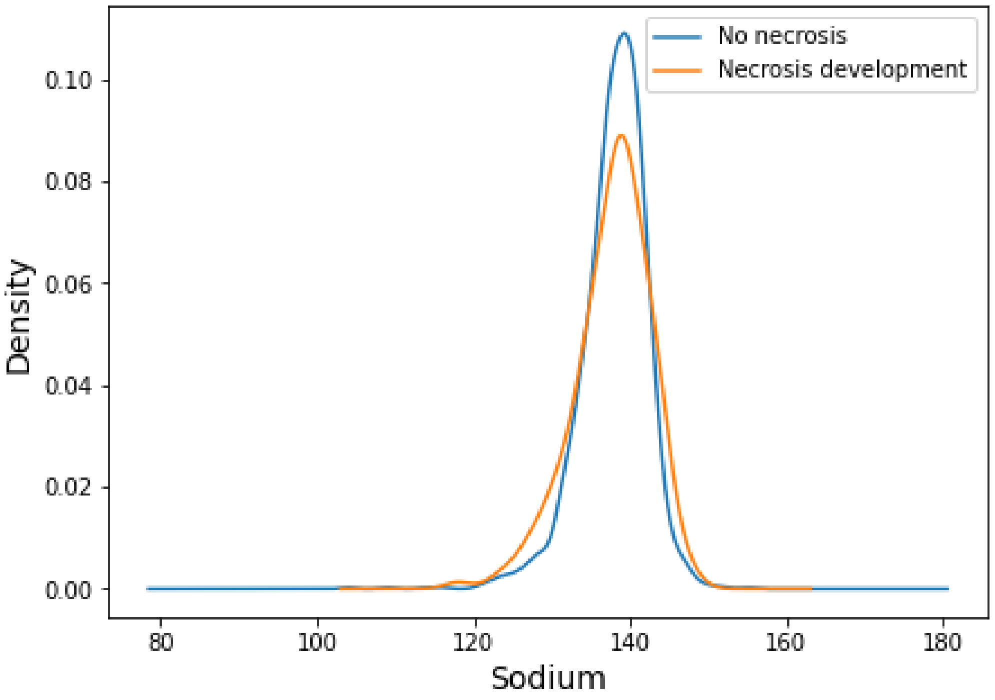

Supplementary Figure 36: The comparison in terms of potassium did not show statistically significant difference between acute pancreatitis patients with and without necrosis development (Kolmogorov–Smirnov test, p=0.576).

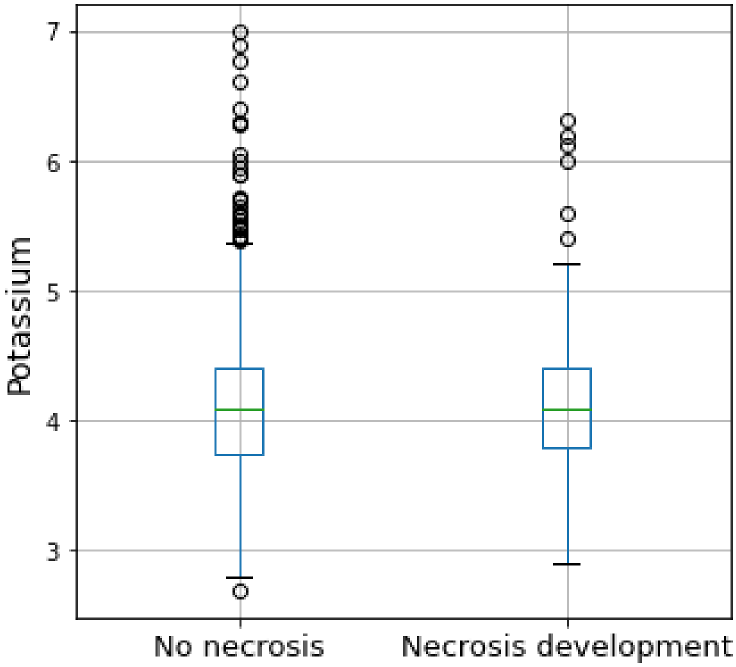

n=1649 **n=176**

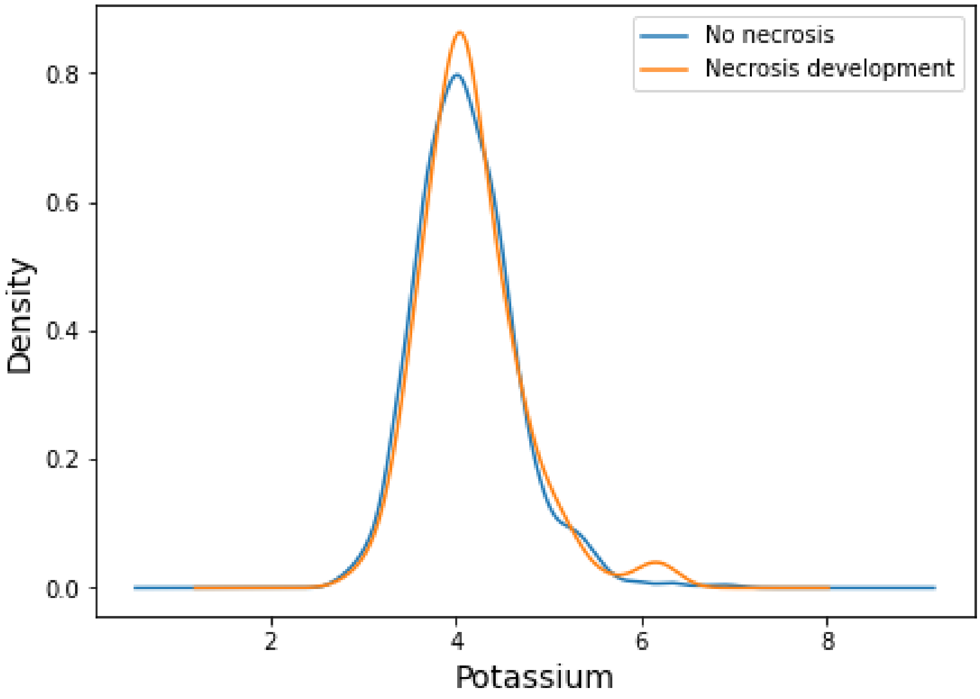

Supplementary Figure 37: The comparison in terms of total protein did not show statistically significant difference between acute pancreatitis patients with and without necrosis development (Kolmogorov–Smirnov test, p=0.598).

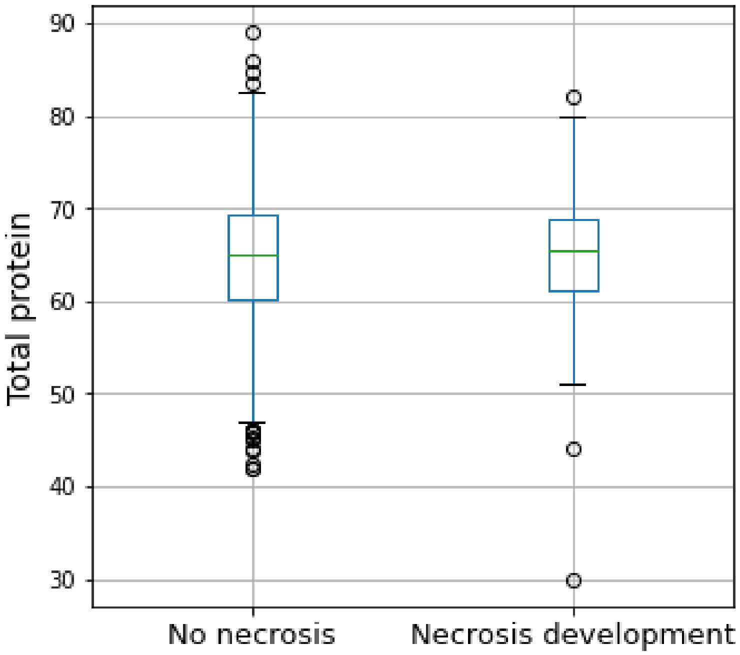

n=800 **n=100**

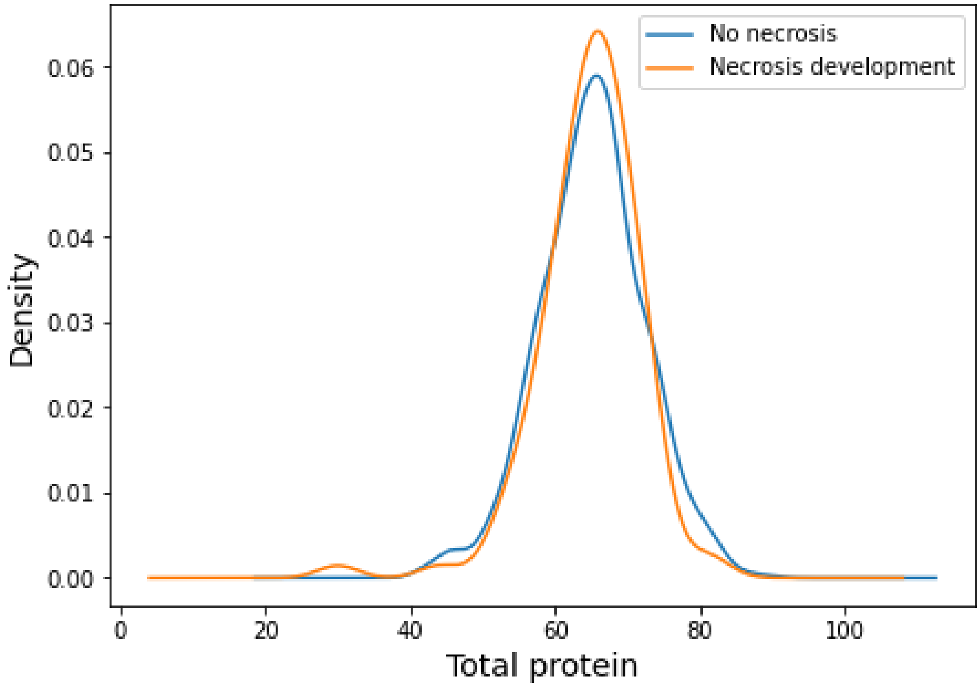

Supplementary Figure 38: The comparison in terms of albumin did not show statistically significant difference between acute pancreatitis patients with and without necrosis development (Kolmogorov–Smirnov test, p=0.822).

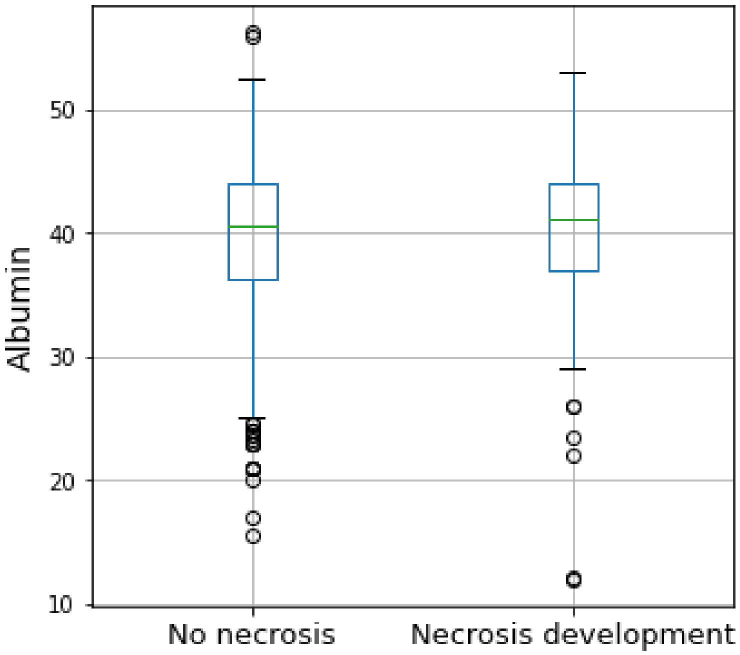

n=940 **n=119**

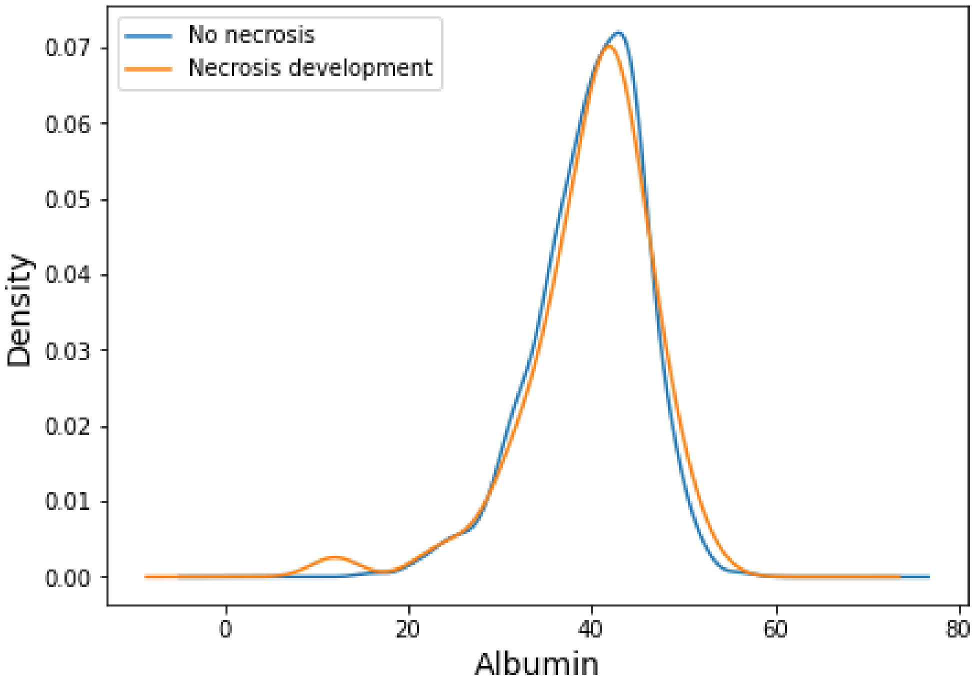

Supplementary Figure 39: The comparison in terms of cholesterol showed statistically significant difference between acute pancreatitis patients with and without necrosis development (Kolmogorov–Smirnov test, p=0.002).

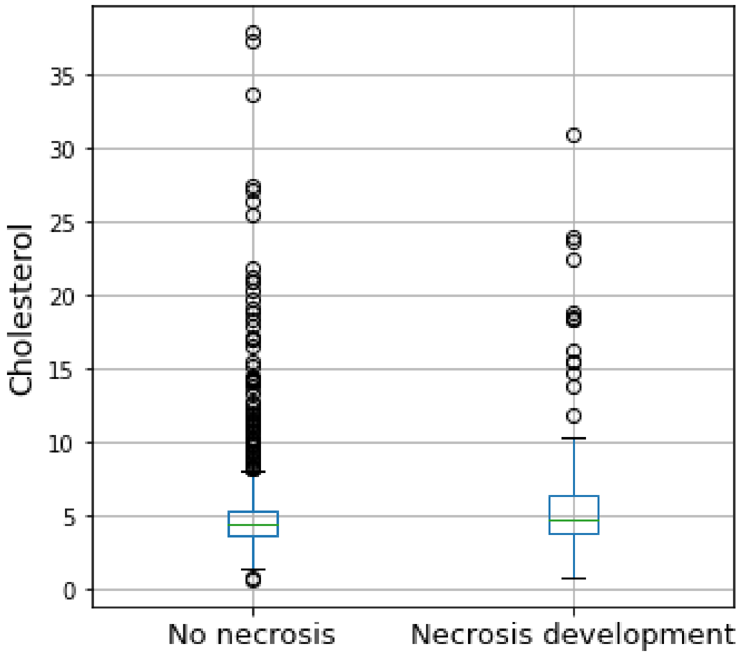

n=1124 **n=133**

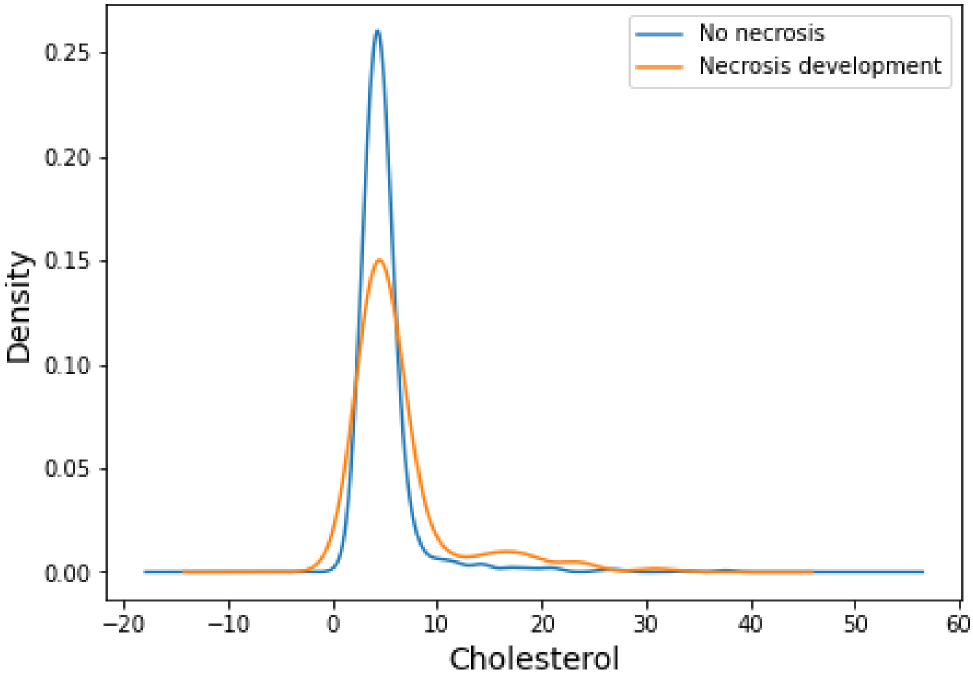

Supplementary Figure 40: The comparison in terms of triglyceride showed statistically significant difference between acute pancreatitis patients with and without necrosis development (Kolmogorov–Smirnov test, p=0.003).

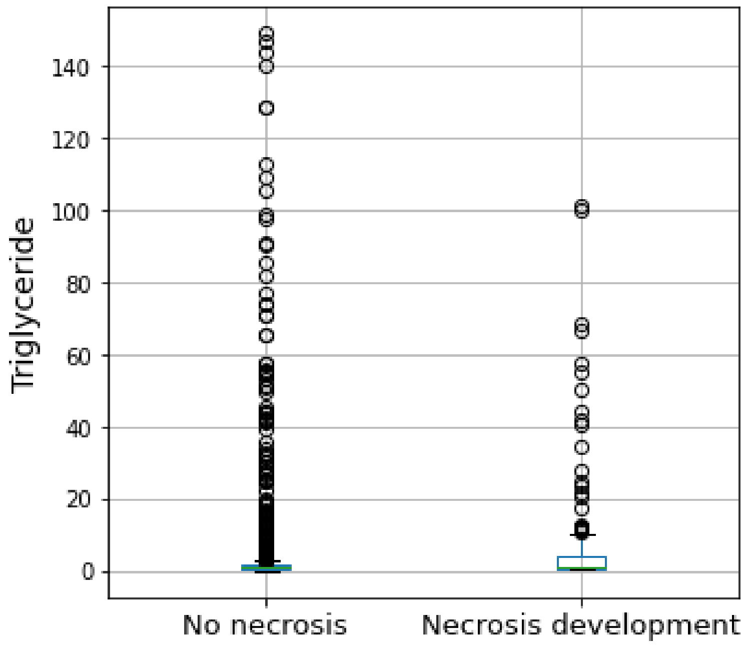

n=1233 **n=144**

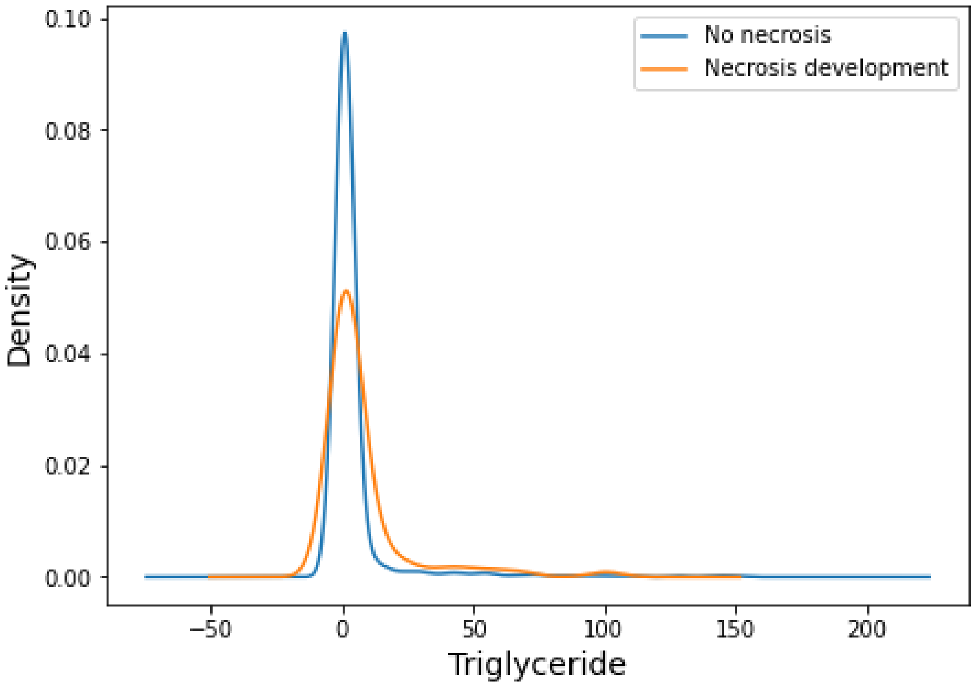

Supplementary Figure 41: The comparison in terms of aspartate transaminase showed statistically significant difference between acute pancreatitis patients with and without necrosis development (Kolmogorov–Smirnov test, p=0.008).

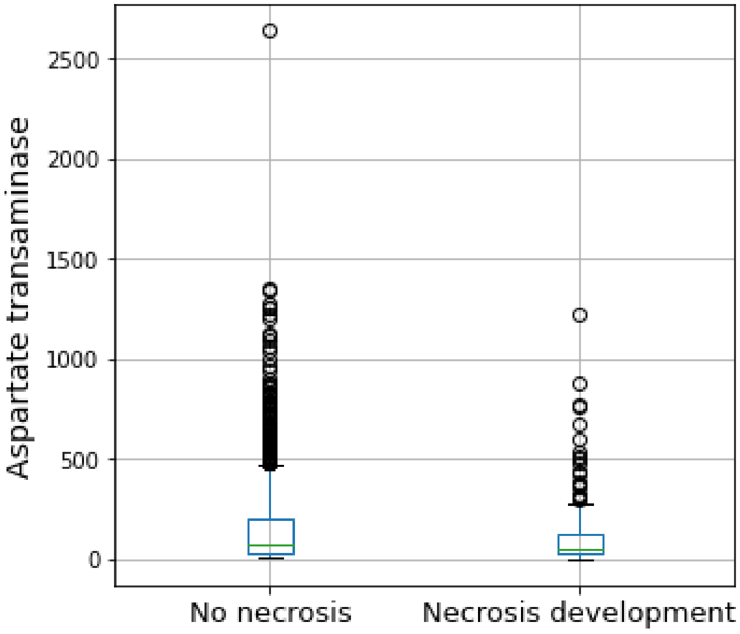

n=1562 **n=168**

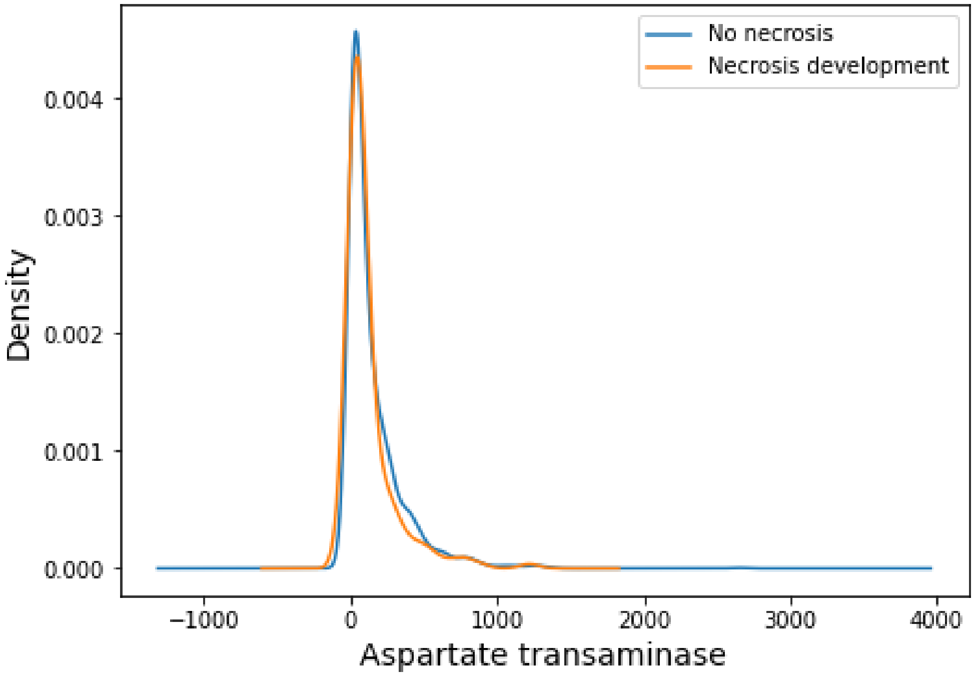

Supplementary Figure 42: The comparison in terms of alanine transaminase showed statistically significant difference between acute pancreatitis patients with and without necrosis development (Kolmogorov–Smirnov test, p=0.018).

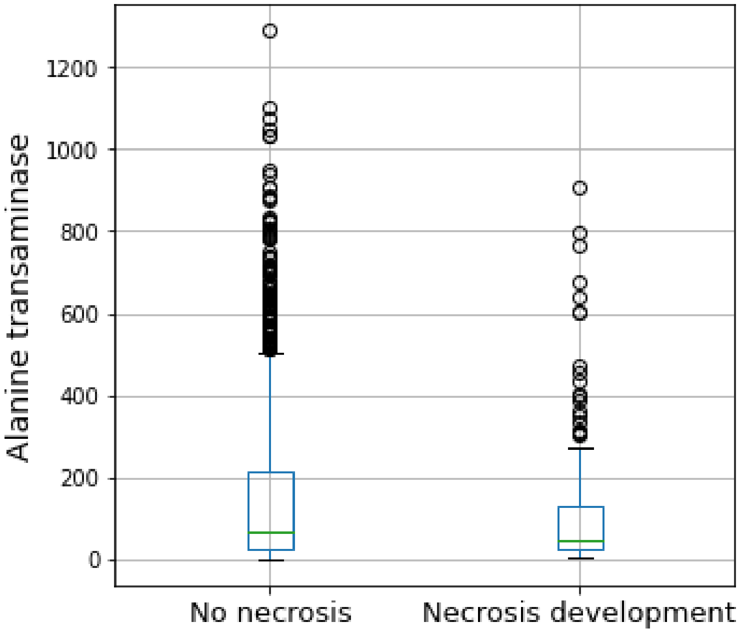

n=1525 **n=167**

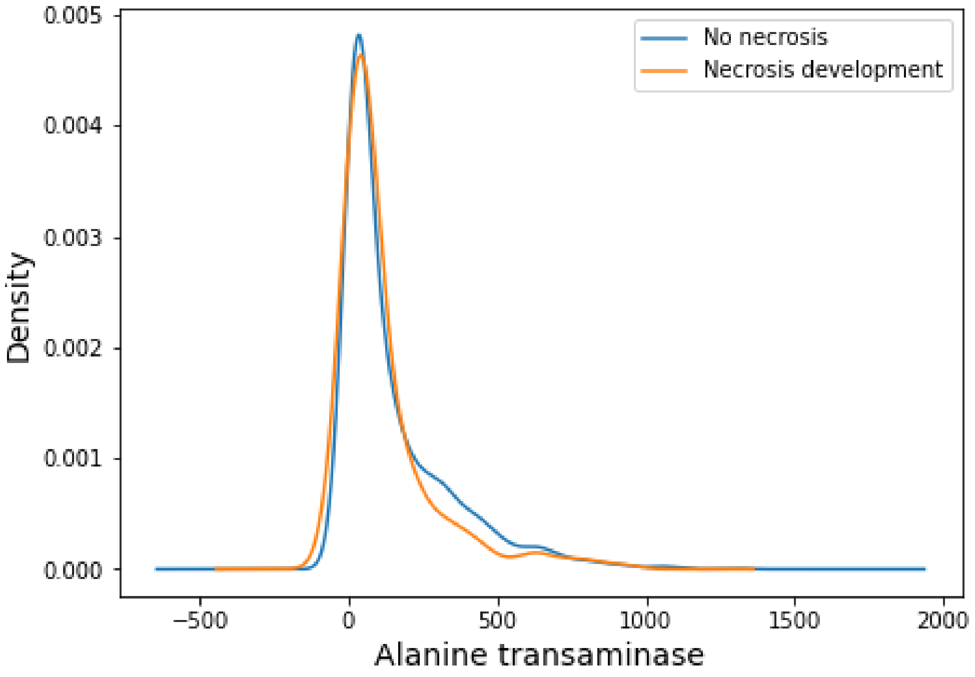

Supplementary Figure 42: The comparison in terms of gamma-glutamyl transferase did not show statistically significant difference between acute pancreatitis patients with and without necrosis development (Kolmogorov–Smirnov test, p=0.060).

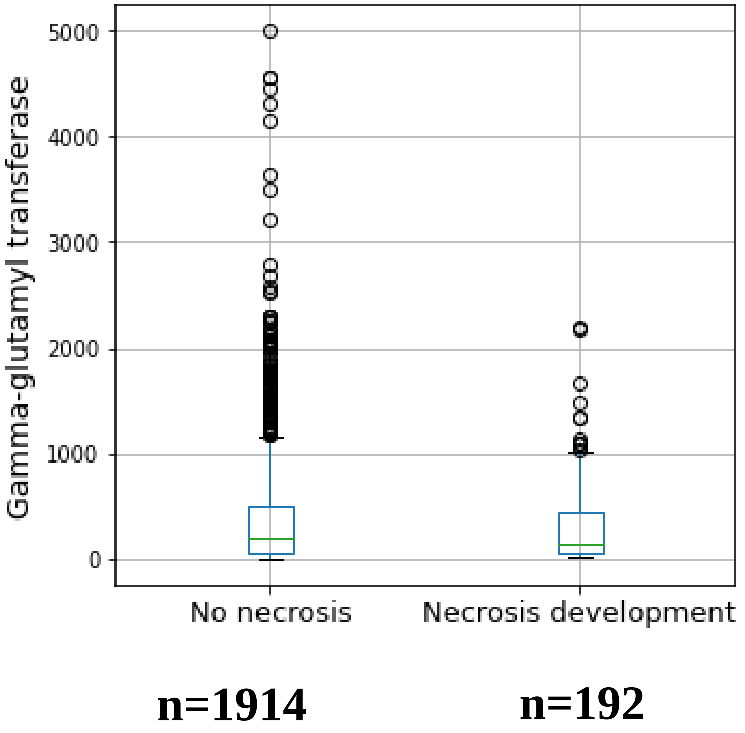

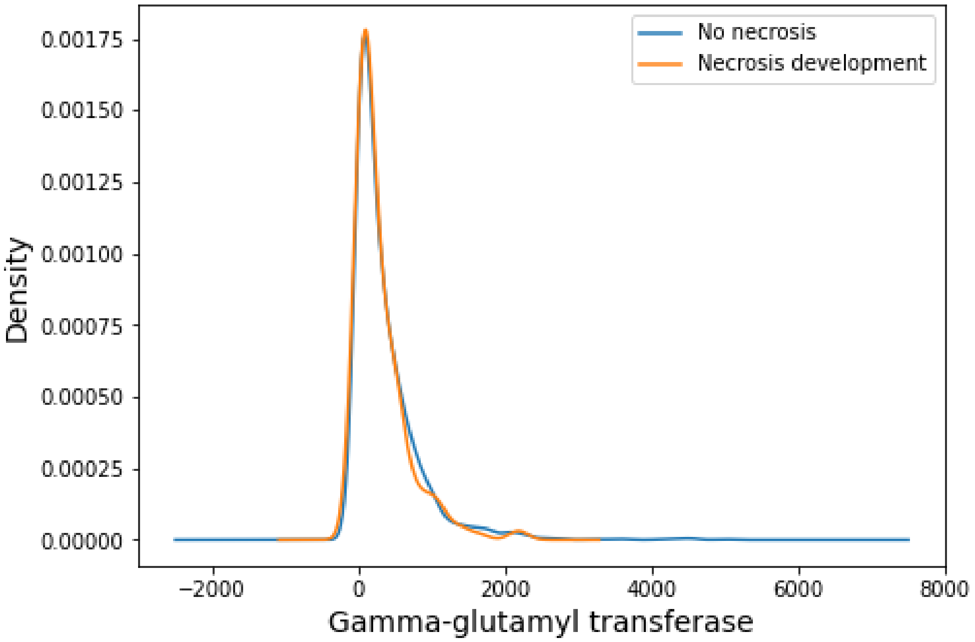

Supplementary Figure 43: The comparison in terms of total bilirubin showed statistically significant difference between acute pancreatitis patients with and without necrosis development (Kolmogorov–Smirnov test, p<0.001).

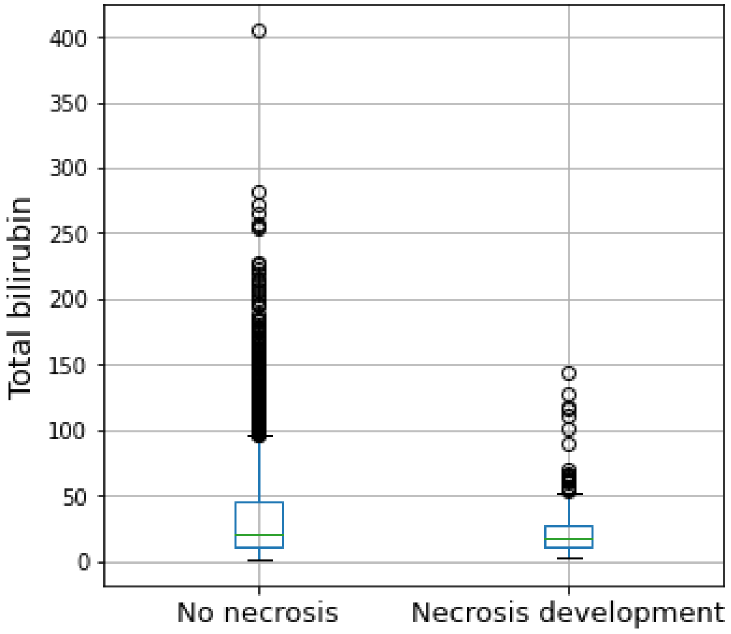

n=1959 **n=200**

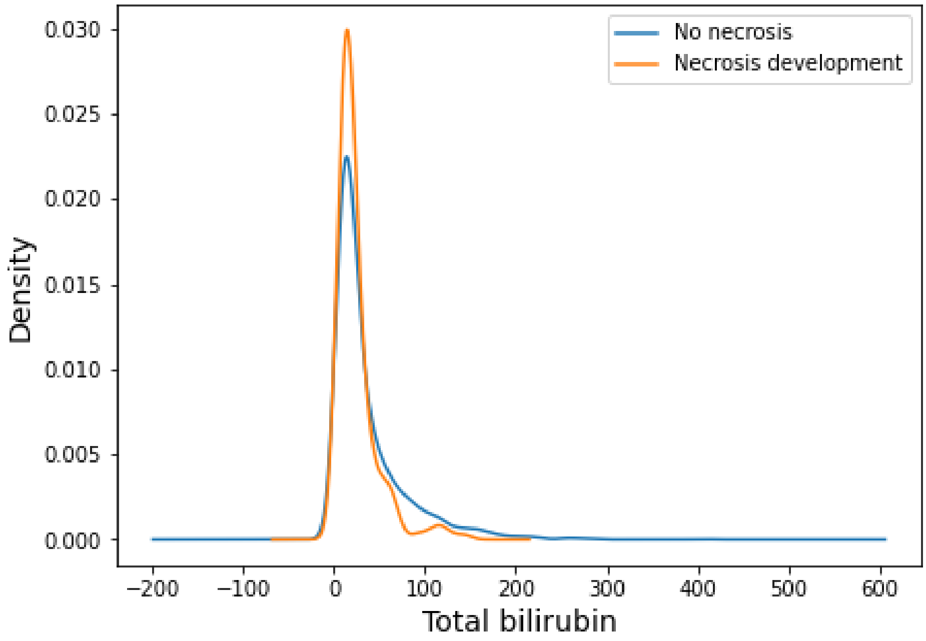

Supplementary Figure 44: The comparison in terms of direct bilirubin showed statistically significant difference between acute pancreatitis patients with and without necrosis development (Kolmogorov–Smirnov test, p=0.004).

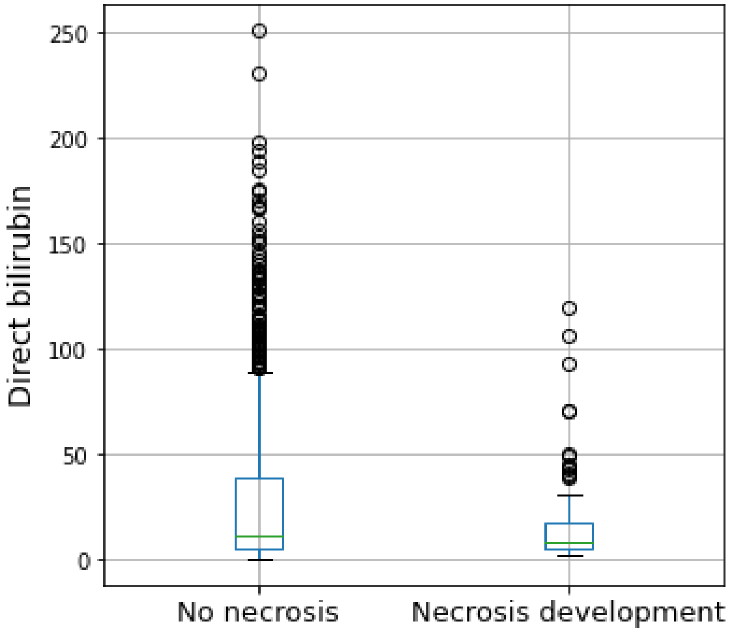

n=1098 **n=107**

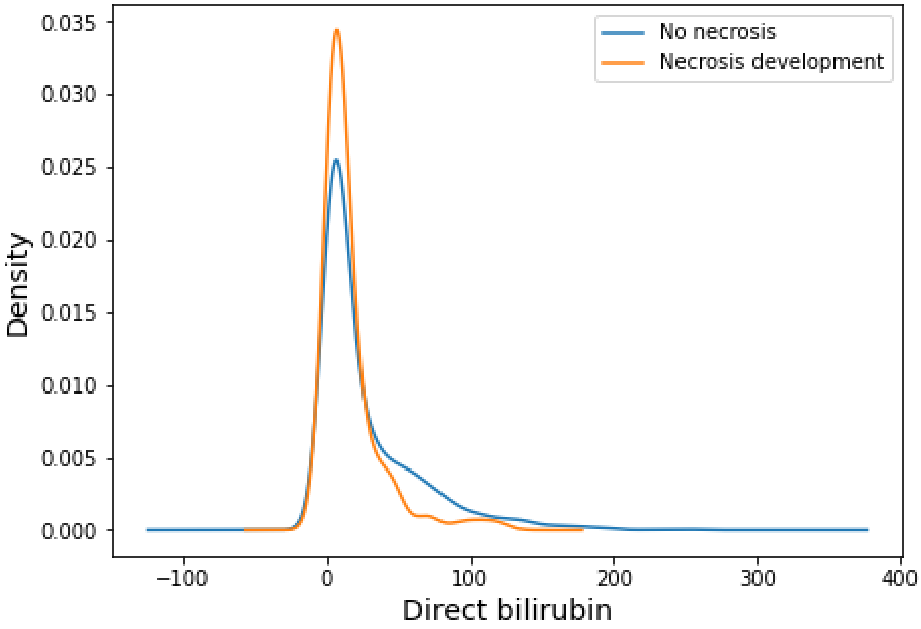

Supplementary Figure 45: The comparison in terms of alkaline phosphatase showed statistically significant difference between acute pancreatitis patients with and without necrosis development (Kolmogorov–Smirnov test, p<0.001).

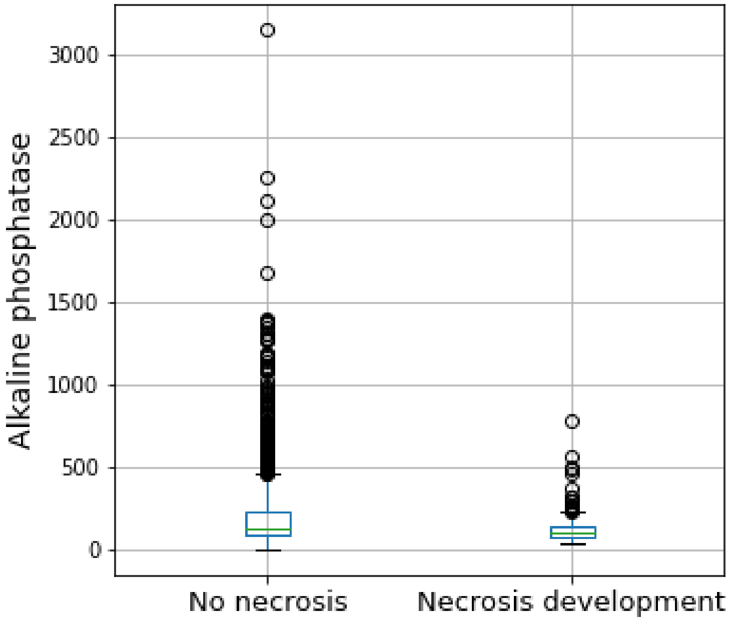

n=1915 **n=194**

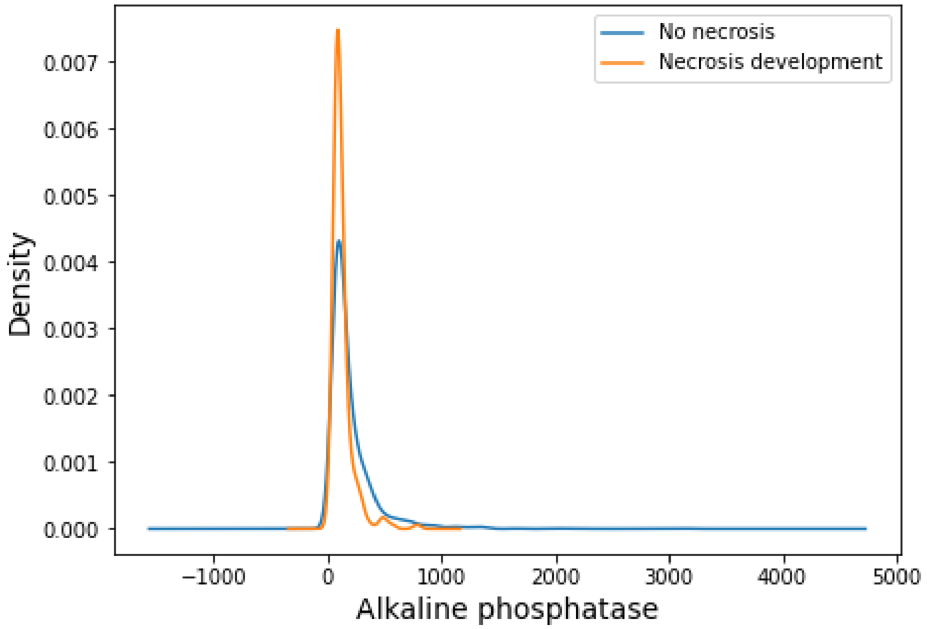

Supplement: Supplementary file 2 — Supplementary Figures. [file 41598_2022_11517_MOESM2_ESM.pdf]
